# Neotropical *Copestylum* Macquart (Diptera: Syrphidae) Breeding in Fruits and Flowers, Including 7 New Species

**DOI:** 10.1371/journal.pone.0142441

**Published:** 2015-11-18

**Authors:** Antonio Ricarte, M. Ángeles Marcos-García, E. Geoffrey Hancock, Graham E. Rotheray

**Affiliations:** 1 Department of Natural Sciences, National Museums Collection Centre, Edinburgh, Scotland, United Kingdom; 2 Centro Iberoamericano de la Biodiversidad (CIBIO), University of Alicante, San Vicente del Raspeig, Alicante, Spain; 3 The Hunterian (Zoology Museum), University of Glasgow, Glasgow, Scotland, United Kingdom; Nanjing Agricultural University, CHINA

## Abstract

Ten species of *Copestylum* (Diptera: Syrphidae) were reared from fruits and flowers in Costa Rica, Ecuador and Trinidad. Seven were new and in this paper, we describe them, their development sites and the third stage larva and/or the puparium of all ten species. One new synonym is proposed, *Copestylum pinkusi* (Curran) [= *Copestylum cinctiventre* (Curran)]. Similarities and differences between these new and other *Copestylum* species, suggest they separate into two groups, referred to as the Vagum and Cinctiventre species groups. Features characterising these groups for both adult and early stages are assessed. Each species was also distinguished using adult and early stage characters. Within the Vagum group, adults were more disparate morphologically than the larval stage; this was reversed in the Cinctiventre group. Adult colour patterns are probably cryptic in function and for disguise. Vagum species have disruptive marks, while the Cinctiventre species have reflective colours. Biologically, the groups are almost distinguished by larval development sites. Vagum species use predominantly fruits and have a larval stage that is relatively generalised in form and habit. Cinctiventre species are confined to developing in flowers and the larva is more specialised. A key to both adult and early stages of all ten species is provided.

## Introduction

The chiefly Neotropical genus, *Copestylum* Macquart, 1846 (Diptera: Syrphidae), is exceptionally large with 400+ species and many more undescribed [1, 2]. Adults are distinguished by a plumose arista, straight or recessive apical crossvein and pleuron with bare anterior anepisternum and posterior anepimeron [3]. Larval stages are saprophagous in a huge array of decaying plant materials and in the third (= final) stage, are distinguished from other syrphid larvae by mandibles that are inverted into the thorax and anal segment divided into two sections, the basal section of which has two pairs of lappets of unequal length [2]. Enabling reference to species sharing distinctive biological or morphological features, 26 heuristic, yet putatively monophyletic, species groups are recognised within *Copestylum* [2]. All species considered here, fall into one or other of two of these groups that are here referred to by the specific name of a common member species, the Vagum and Cinctiventre groups.

Within *Copestylum* many taxonomic and nomenclatural problems exist with interspecific variation negligible between some species and greater in others [4]. Intraspecific variation also occurs, but is poorly understood and documented [1]. In this paper, the third of a series dealing with reared *Copestylum* [2, 5], three named and seven new species obtained mainly from fruits and flowers are investigated that have necessitated dealing with these problems.

## Materials and Methods

Adults were obtained mainly by finding and rearing larvae and puparia in areas of decay in live fruits or in wholly decayed fruits, as well as in live and decaying flowers of understorey plants in Costa Rica, Ecuador and Trinidad at various times from 1998 to 2011. In Costa Rica, specimens were collected from national parks under the INBio-MINAE (‘Ministerio de Ambiente, Energía y Mares’, Costa Rica) agreement for the Costa Rican National Biodiversity Inventory organised by the Costa Rican National Institute of Biodiversity. In Ecuador, permission to collect was obtained from Professor Giovanni Onore, Director of the ‘Fundación Otonga’ who owns and manages this cloud forest reserve. In Trinidad, specimens were collected under permission from the Ministry of the Environment and Water Resources, Government of Trinidad to the University of Glasgow Expedition Society. The fieldwork did not involve endangered or threatened species.

Larvae were reared by placing them in plastic bags or tubs with small quantities of decay from the material they were collected from or whole, infested fruits or flowers were placed into containers. Pin holes were made to ensure air exchange and containers were stored in cool, shaded conditions. Every few days, they were examined for puparia. To ensure association with the correct adult, puparia were removed and reared individually in separate containers. After allowing 1–2 days for hardening, emerged adults were usually fixed by freezing. Adults were identified using keys, original descriptions and named specimens including holotypes, in various collections, particularly the Instituto Nacional de Biodiversidad, Costa Rica, United States National Museum, Washington, U.S.A. and the Natural History Museum, London, UK.

**Fig 1:**
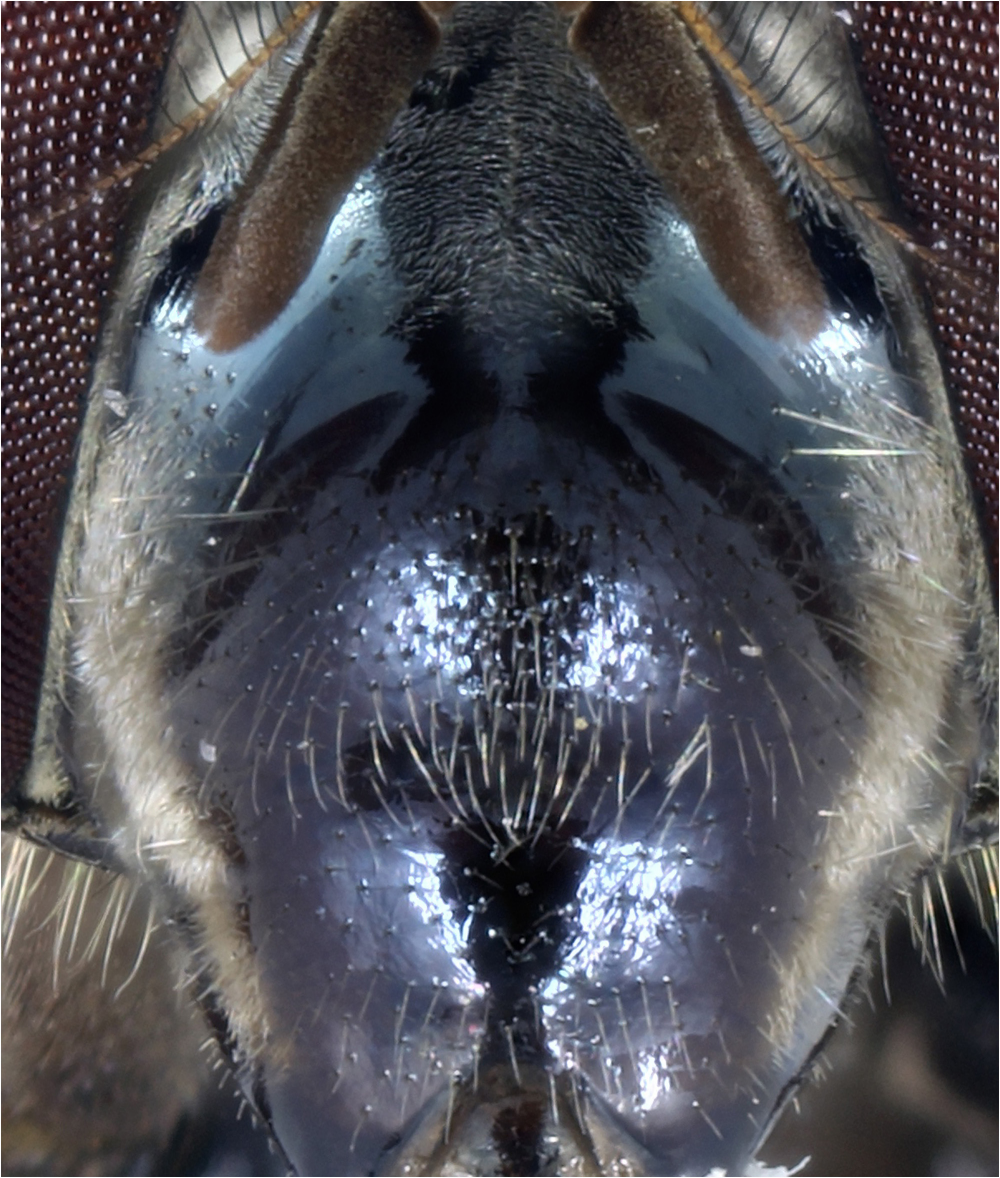
*Copestylum araceorum* sp. nov. Male holotype, face, anterior view, dust between the antennae and white tomentum along the sides.

**Fig 2:**
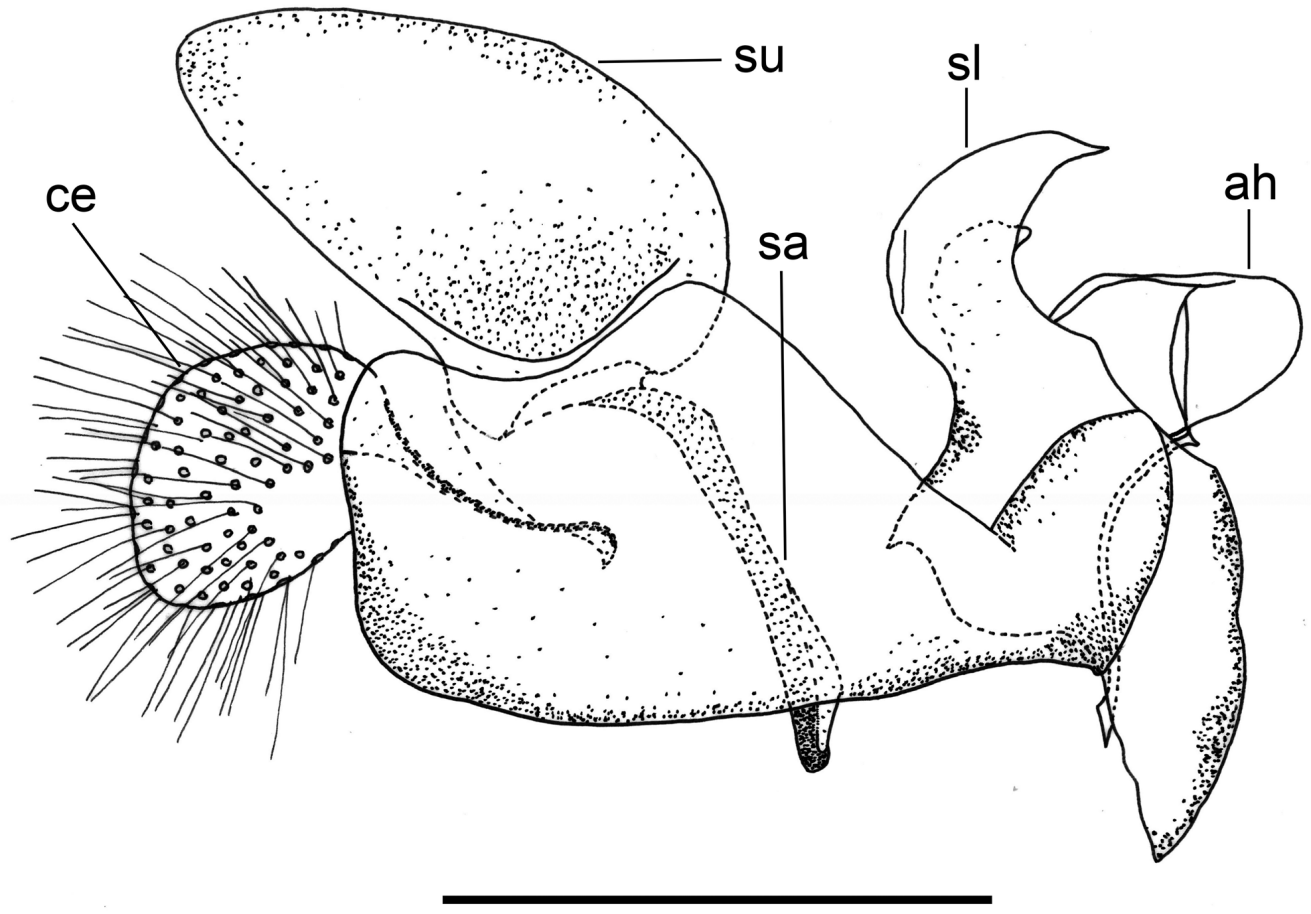
*Copestylum araceorum* sp. nov. Male holotype, genitalia, lateral view. Legend: ah, aedeagal hood; ce, cercus; sa, surstylar apodeme; sl, superior lobe; su, surstylus. Scale line = 0.5mm.

For morphological descriptions, adult body length was measured from the apex of the frontal prominence on the head to the apex of abdominal tergite 5, thorax width from tegula to tegula and wing length from the tegula at the wing base to the apex. In the text, T = tergite, S = sternites. Male genitalia were examined by relaxing specimens and removing genitalia with an entomological pin, clearing in a hot solution of KOH for up to 10 minutes, immersing in glacial acetic acid to remove excess KOH, washing in 70% alcohol and storing in microvials containing glycerol. Drawings were made with a FSA 25 PE drawing tube attached to a stereo microscope. Adult images were made using a Canon 7d Mark ii camera with a Canon MP-E 65mm f 2.8 lens and an automated focus stacker, Cognisys Stackshot 3X mounted on a Kaiser R1 copy stand, stacked using Zerene Stacker software and adjusted using Adobe Photoshop. Early stage images were made using a Canon Powershot S100 camera attached to a Wild M5 stereo microscope or with the built in camera of an Olympus BX51 compound microscope. Under sections headed ‘material examined’, L = date larva collected; P = date puparium formed; A = date adult emerged; other abbreviations are indicated in brackets (‘…’) when first mentioned. Terminology for the adult stage follows Thompson [6], except that we distinguish between two pollinose states referred to here as dust which is short microtrichia, such as appears under the antennae and tomentum which consists of longer, dense, planiform (flattened), silvery white to yellow microtrichia, such as appears on the sides of the face in certain Cinctiventre species (Fig 1). We also use the term, aedeagal hood, to refer to a morphologically variable structure at the apex of the hypandrium, a position shared with the hook-like, superior lobes which are dorsal relative to aedeagal hood (Fig 2). Terminology for the early stages follows Rotheray & Gilbert [7].

Species group diagnoses are based on shared characters from member species considered here and obtained from the external morphology of adults, including male genitalia and for early stages, the third stage larva and/or the puparium. Species diagnoses include specific states of variable characters at group level and unique or unique combinations of characters that distinguish species. These and other features are further compared and contrasted under taxonomic notes. Within species groups, the sequence of species is alphabetical by specific name. Original descriptions of three reared species that were named prior to this study lacked the detail required to distinguish them from other species in the same group. Original descriptions and types of these three species were assessed and descriptions amplified by additional character data, noting particularly, where reared material differed from type specimens. Material of the 10 species dealt with here is deposited in the following institutions: AMNH = American Museum of Natural History, New York, USA; CEUA = Colección Entomológica de la Universidad de Alicante, Alicante, Spain; HM = Hunterian Museum, University of Glasgow, Glasgow, UK; INBio = Instituto Nacional de Biodiversidad, Santo Domingo de Heredia, Costa Rica; NHM = Natural History Museum, London, UK; NMS = National Museums Scotland, Edinburgh, UK; NMW = Naturhistorisches Museum Wien, Austria.

### Nomenclatural Acts

The electronic edition of this article conforms to the requirements of the amended International Code of Zoological Nomenclature, and hence the new names contained herein are available under that Code from the electronic edition of this article. This published work and the nomenclatural acts it contains have been registered in ZooBank, the online registration system for the ICZN. The ZooBank LSIDs (Life Science Identifiers) can be resolved and the associated information viewed through any standard web browser by appending the LSID to the prefix “http://zoobank.org/”. The LSID for this publication is: urn:lsid:zoobank.org:pub: E7CA268F-CB57-4483-A0B8-6990D194FBB0. The electronic edition of this work was published in a journal with an ISSN, and has been archived and is available from the following digital repositories: PubMed Central, LOCKSS.

## Results

### Group and species systematics of *Copestylum* reared from live and dead fruits

#### Vagum species group

*Diagnosis—overall appearance*: adult relatively small and setulose, length 6–10mm, thoracic width 3–4mm, face vittate but not tomentose and with background colour shiny yellow, green or orange; face not particularly extended; scutum with mixed length pile, not forming distinct layers and most orientated posteriorly; no outstanding, long, thick setae along the rear margin of the scutum, although long pile may be present; apex of the scutellum with a linear depression; T1 and most of T2 with a pale mark contrasting with the colour of the scutum, scutellum and the rest of the abdomen; male with aedeagal hood projecting ventrally from the hypandrium, superior lobe with smooth margins. Early stages without specialised spicules; pupal spiracles with bands of spiracles almost reaching the base; prolegs and crochets on abdominal segments 1–4, rarely on 5 or 6.

#### Adult

**Fig 3:**
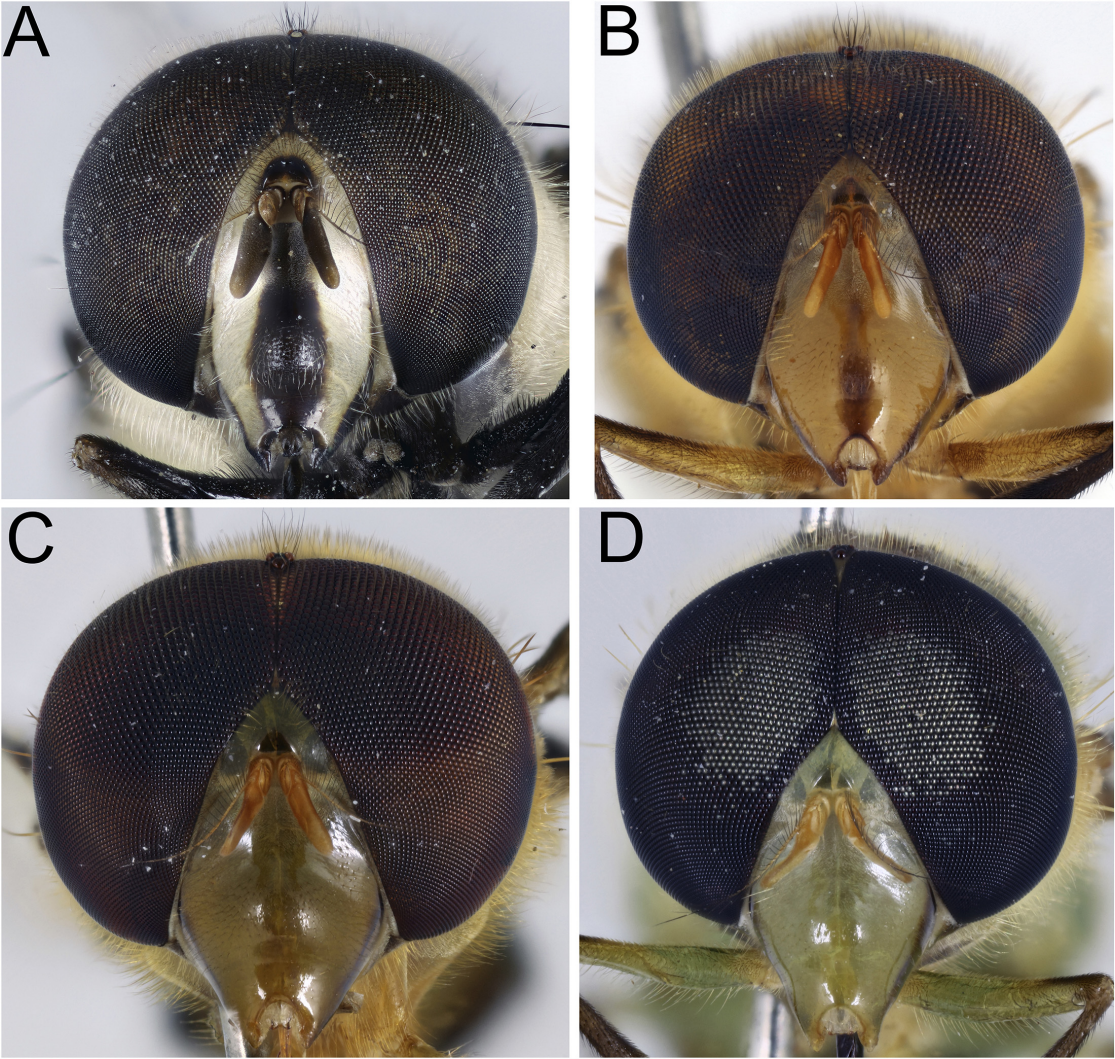
*Copestylum* species of the Vagum group, head, anterior view. A, *Copestylum araceorum* sp. nov., male holotype. B, *Copestylum cyclops* sp. nov., male holotype. C, *Copestylum tenorium* sp. nov., male holotype. D, *Copestylum vagum*, specimen from Costa Rica.

**Fig 4:**
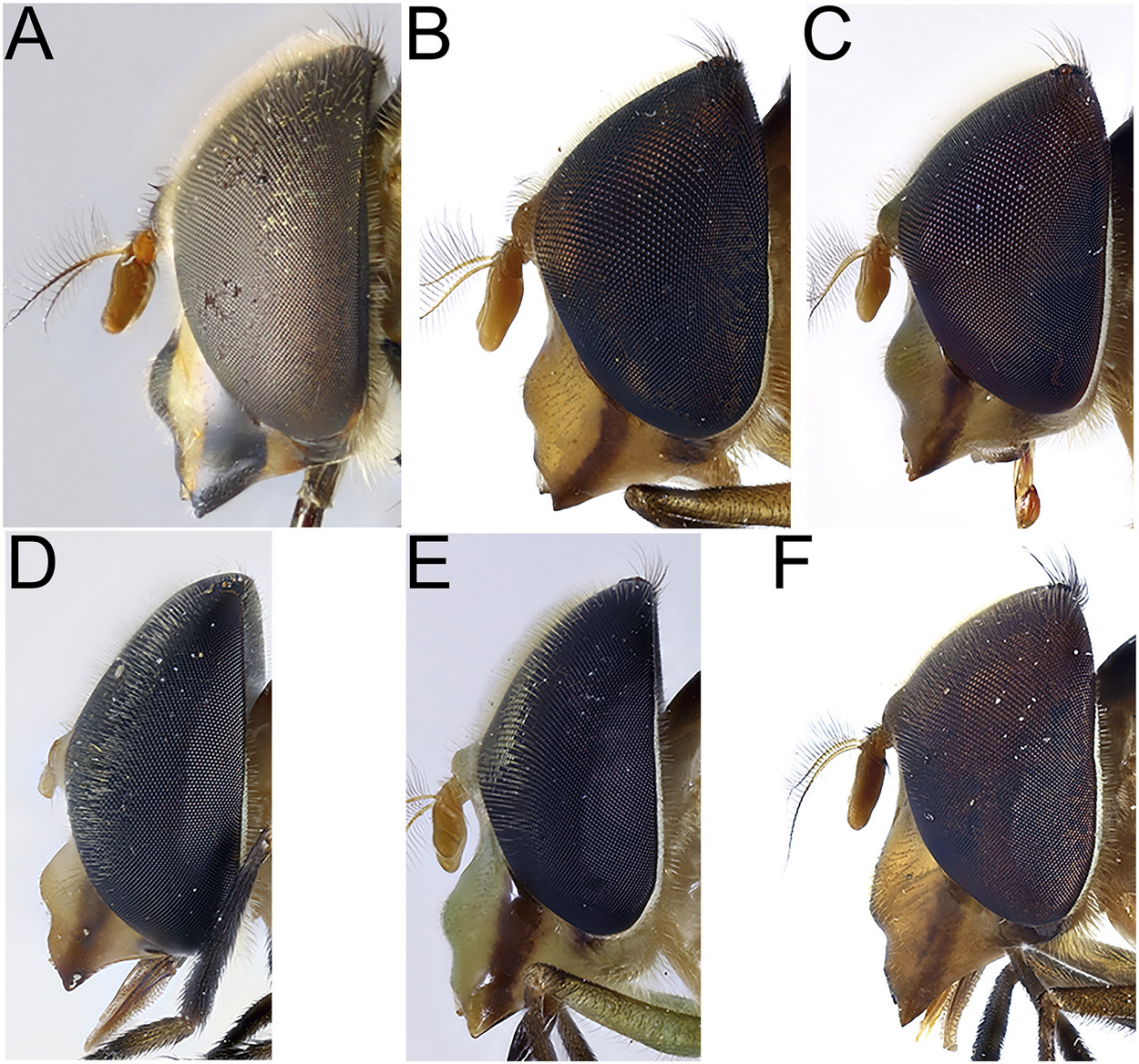
*Copestylum* species of the Vagum group, head, lateral view. A, *Copestylum araceorum* sp. nov., male holotype. B, *Copestylum cyclops* sp. nov., male holotype. C, *Copestylum tenorium* sp. nov., male holotype. D, *Copestylum tigrinum* sp. nov., male holotype. E, *Copestylum vagum* sp. nov., specimen from Costa Rica. F, *Copestylum willistoni* sp. nov., male holotype.

*Head*: face pale pilose with background colour shiny yellow, green or orange (Fig 3B–3D); face with or without a central black vitta from antennae to the mouth (Fig 3A and 3B), sides of the face and the rear corner of the gena with a brown to black vitta (Fig 4A–4F); dust confined to extreme eye margins up to the anterior sides of the frontal triangle and meeting in a blotch-like mark under the antennae; facial tubercle present and clearly defined; frontal triangle yellow, lunule either yellow or black; scape and pedicel with orange or black bristles; basoflagellomere yellow sometimes brownish, kidney to lozenge shaped, varying from 1.5–2.5× as long as height with a conspicuous pit on the inner side; arista with dorsal plumes longer than ventral plumes and arista yellow except for black, apical third to half; occiput white pollinose and yellow haired; male eyes with larger facets in the upper, front surface; this part of the eye can be flattened so that the eye in lateral view, appears relatively rounded (Fig 4B and 4C), and the eye front to back, is more than half the maximum height of the eye; this state contrasts with eyes that are more elongate (Fig 4D and 4E) where the eye is, in lateral view, about half as long from front to back than the maximum height of the eye; vertical triangle with length of black or pale setae correlated to flattened or sloping eye, such that setae 2× or more longer than the distance between the front and rear ocelli or setae about this distance respectively; large eye facets covered in pale yellow to orange pile longer than elsewhere; pile extending to about two thirds down the length of the eyes and becoming shorter and absent on the rear margins.

**Fig 5:**
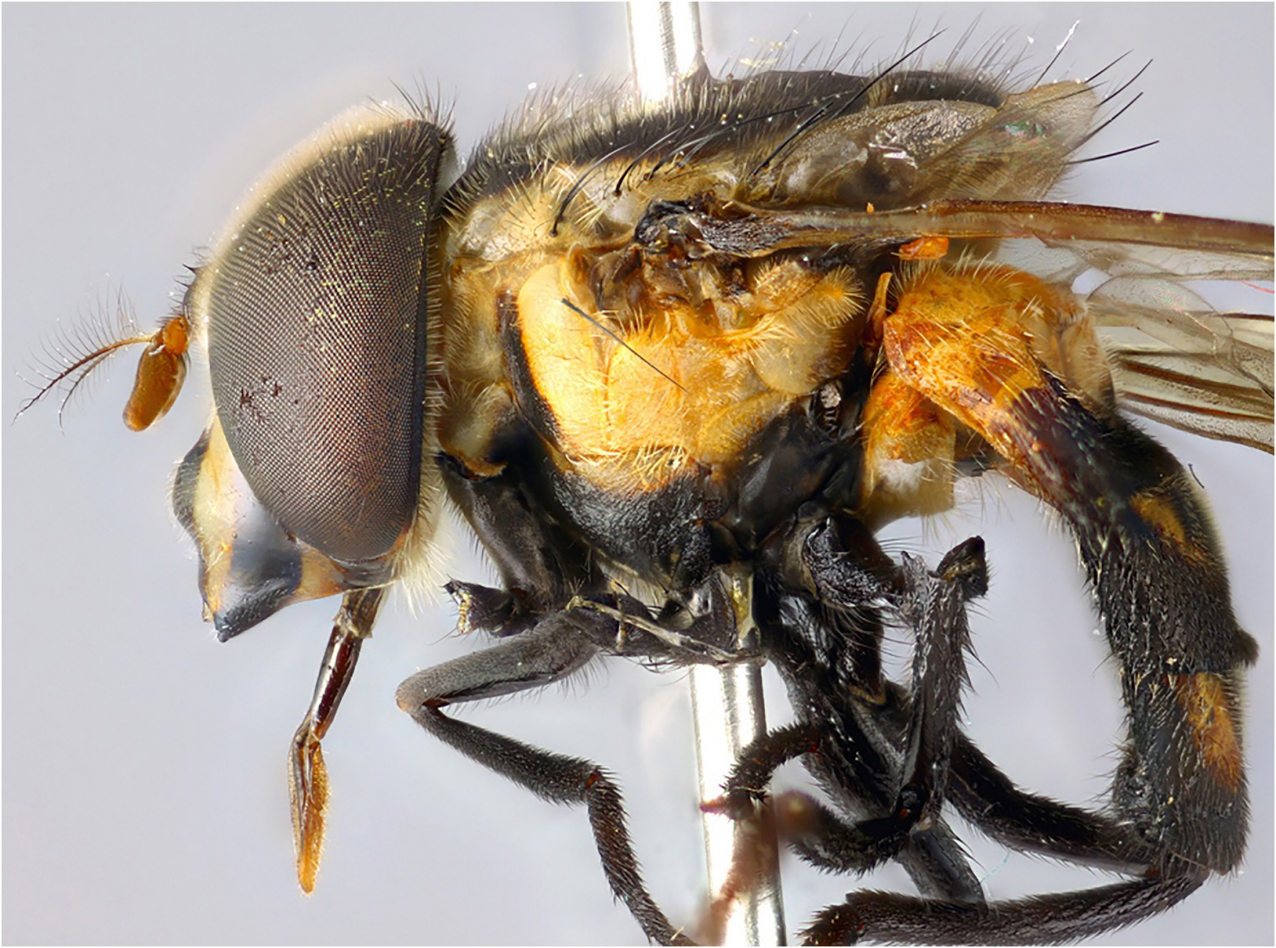
*Copestylum araceorum* sp. nov. Male holotype, side of body, head to the left.

**Fig 6:**
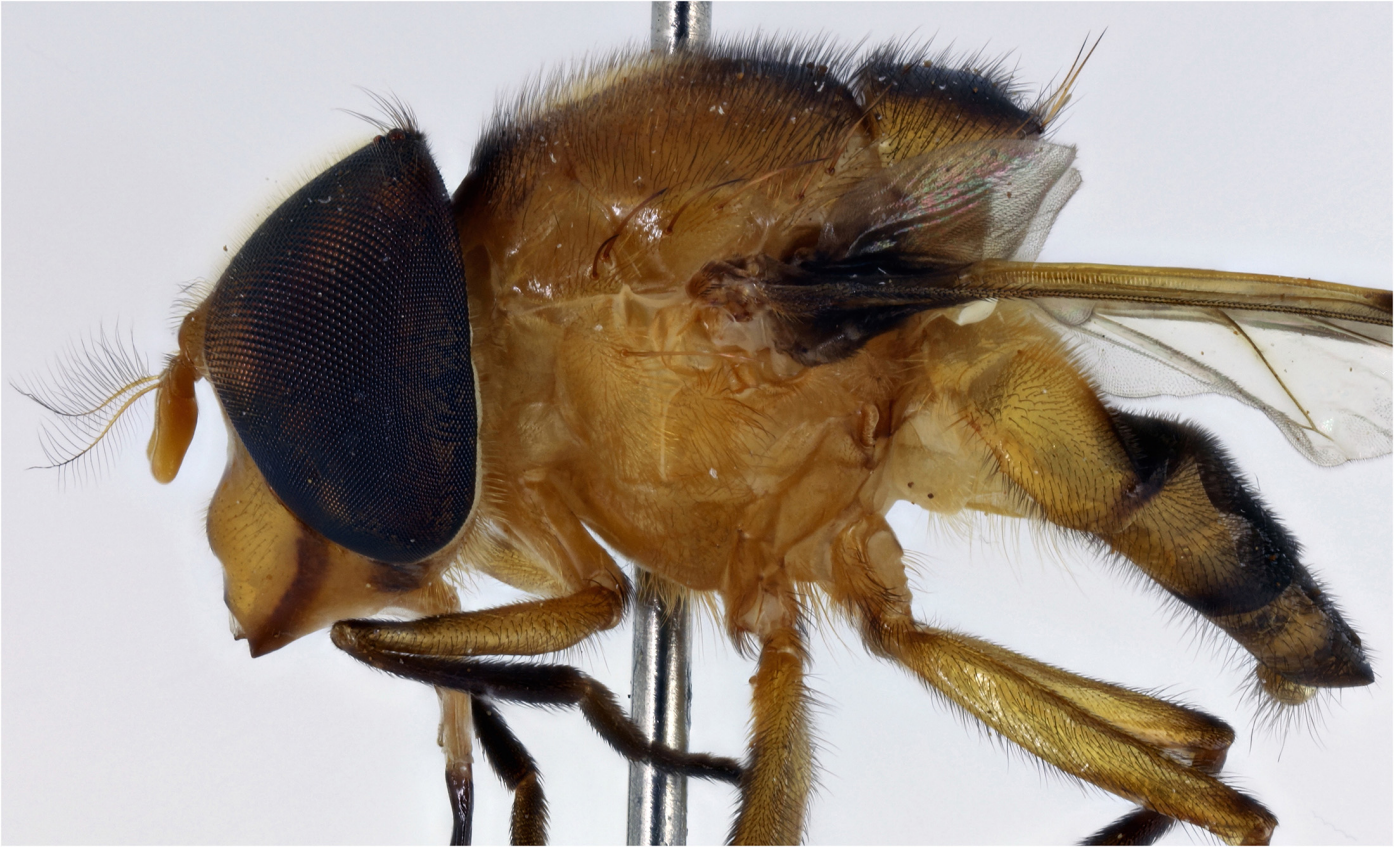
*Copestylum cyclops* sp. nov. Male holotype, side of body, head to the left.

**Fig 7:**
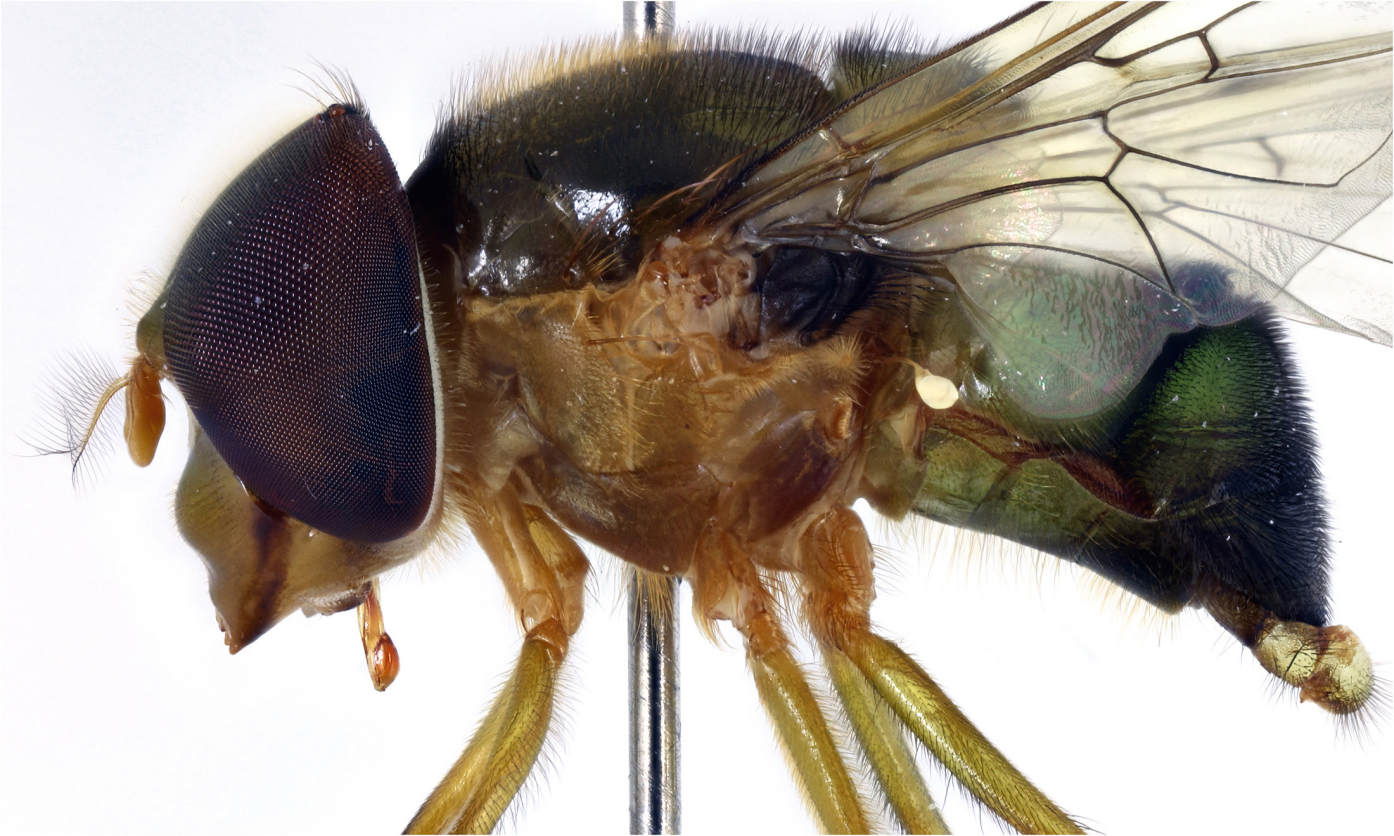
*Copestylum tenorium* sp. nov. Male holotype, side of body, head to the left.

**Fig 8:**
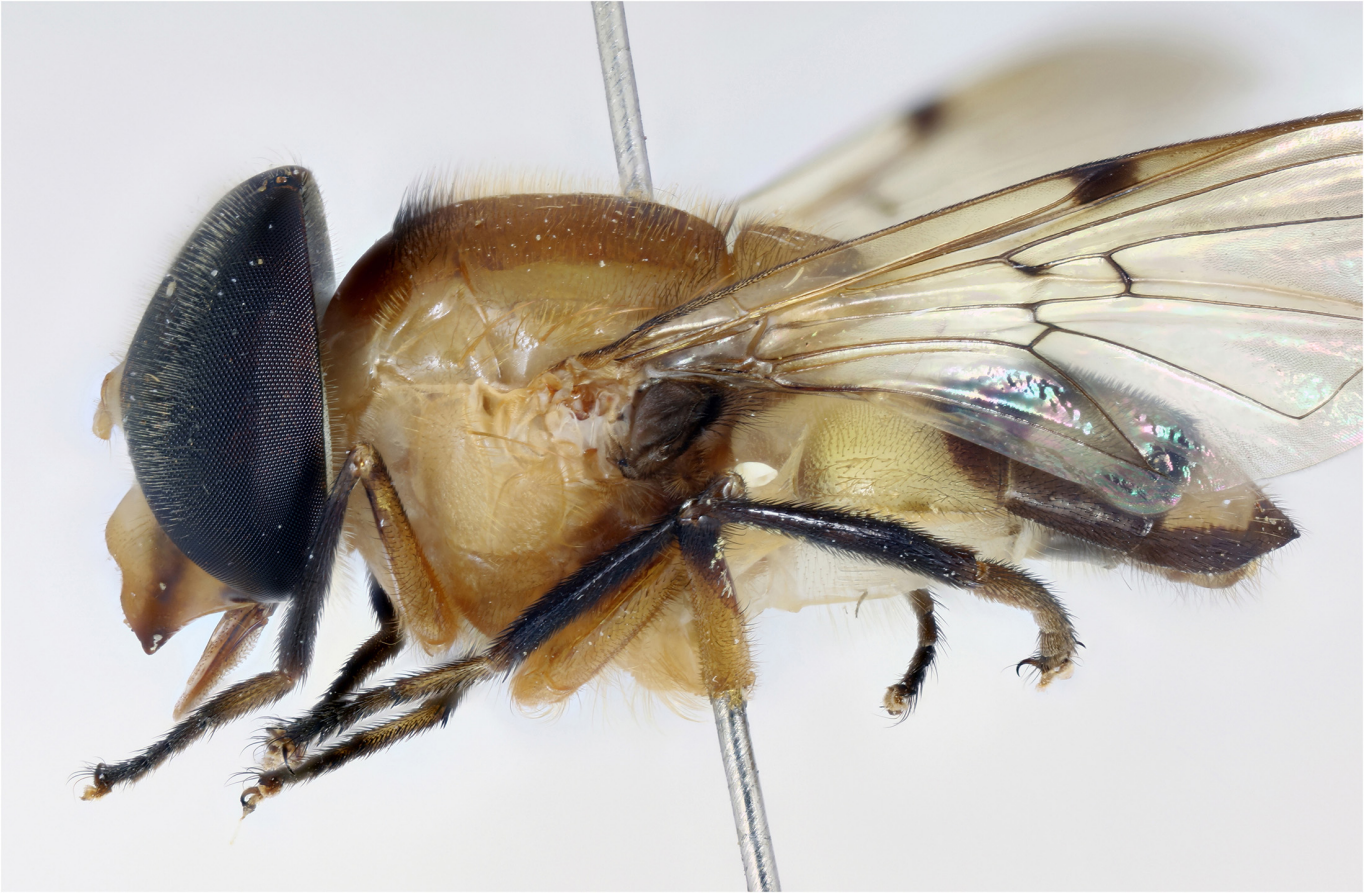
*Copestylum tigrinum* sp. nov. Male holotype, side of body, head to the left.

**Fig 9:**
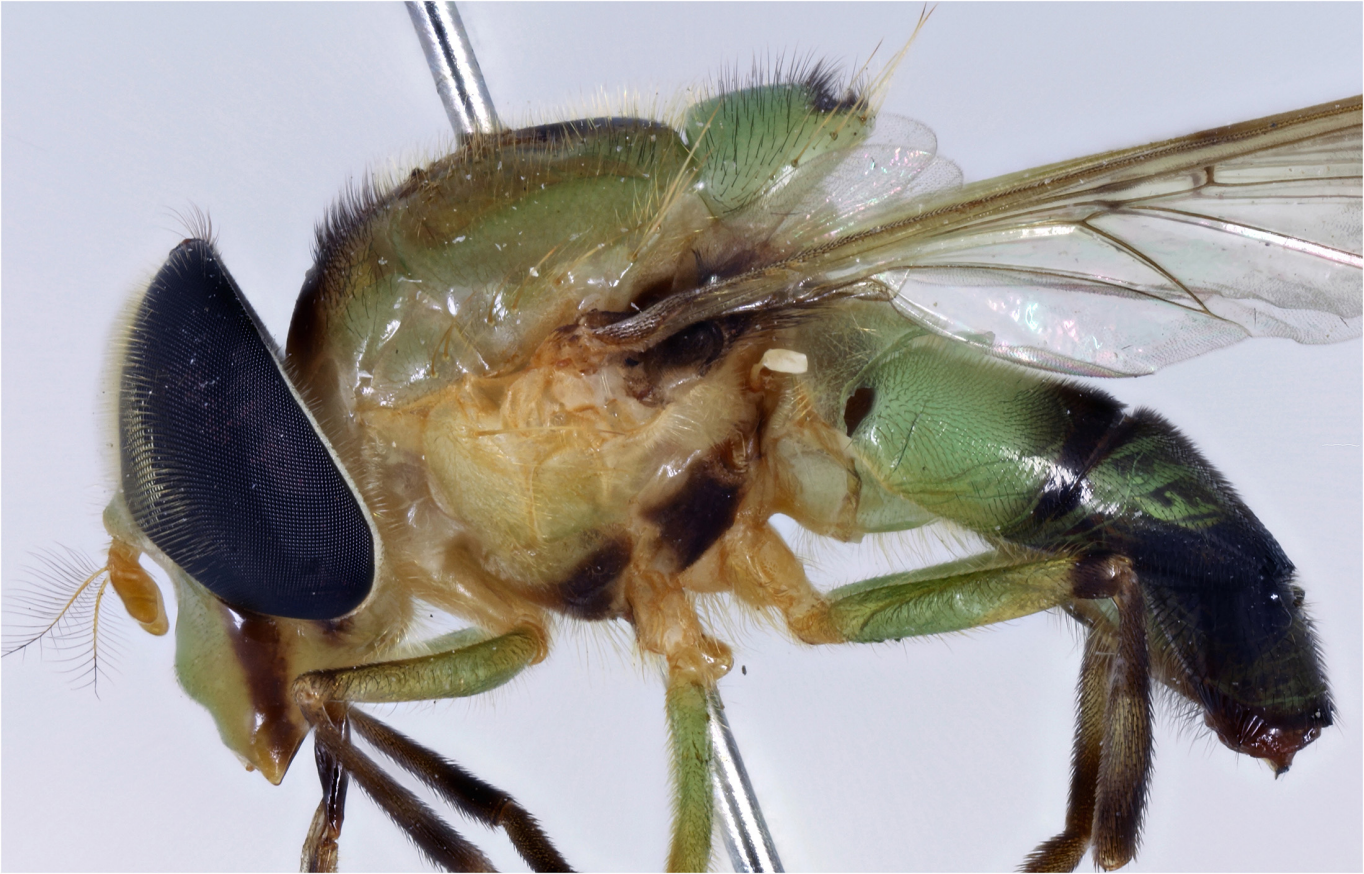
*Copestylum vagum*. Specimen from Costa Rica, side of body, head to the left.

**Fig 10:**
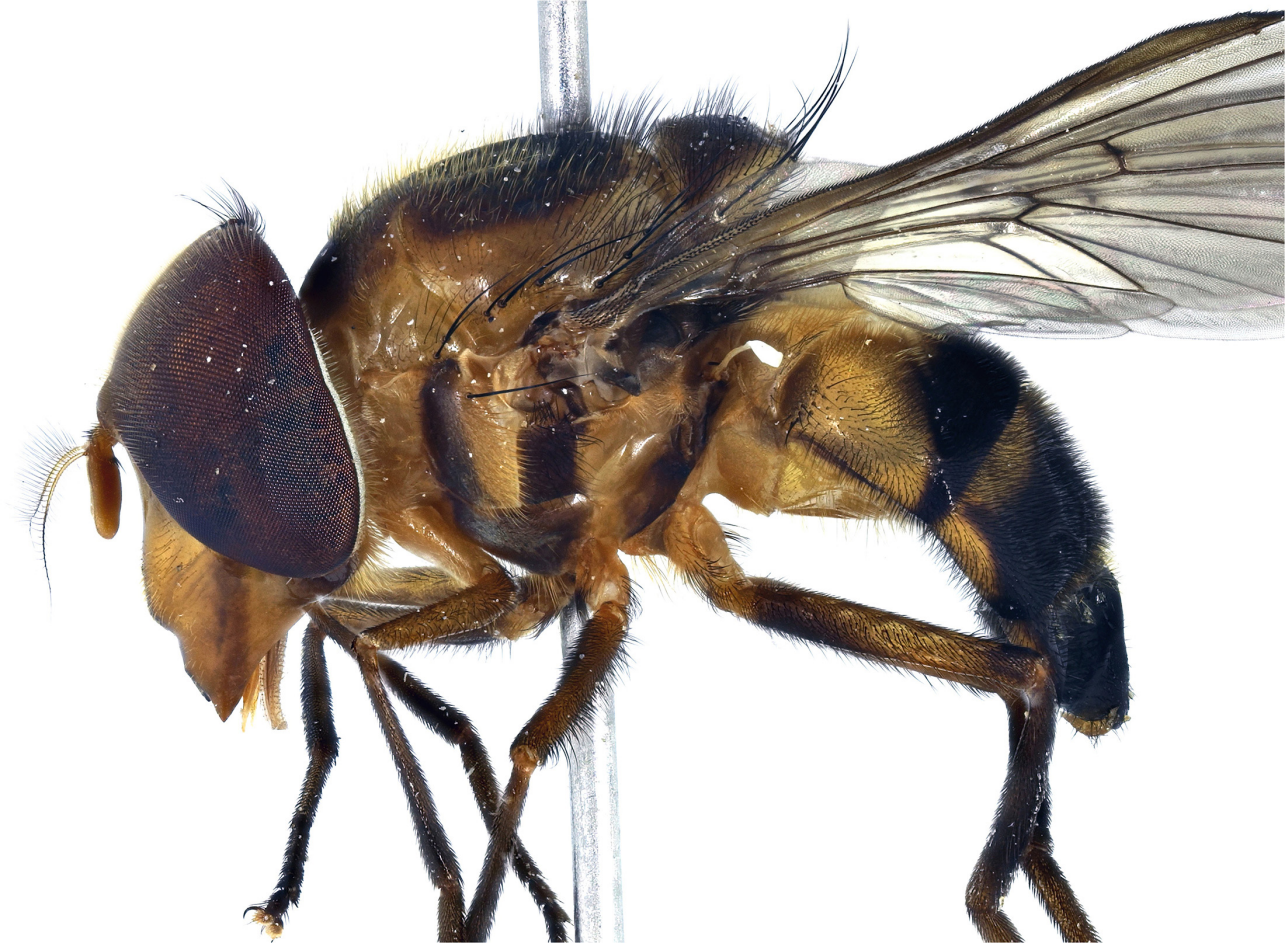
*Copestylum willistoni* sp. nov. Male holotype, side of body, head to the left.

*Thorax*: scutum variably coloured yellow, green, orange with brown or black marks generally shiny or metallic marked and coated in pile of variable length and may or may not be longer at the prescutellum (rear margin), but not forming distinct short and long layers and pile not extensively planiform and orientated in the same, not opposing directions so that a shifting pattern of blotches and stripes according to the angle of view is not present; pile longer in males than females; notopleuron always with 2 setae, 2 to 4 above wing insertion and 2 to 3 on the postalar callus, setae yellow or black; setae absent along the rear margin of the scutum, although longer pile usually present, especially in males; pleuron variably coloured, usually yellow, often with black marks and darkened posteriorly (Figs 5–10); scutellum usually with a variably deep, apical depression and variably coloured and with pile of variable size and colour and setae round the margin; scutellar pile varying in density, least dense medially and pile shorter within the depression; wings extensively microtrichose, rarely marked; legs variably coloured; apex of mid femora may or may not have a group of noticeably, longer setae.

**Fig 11:**
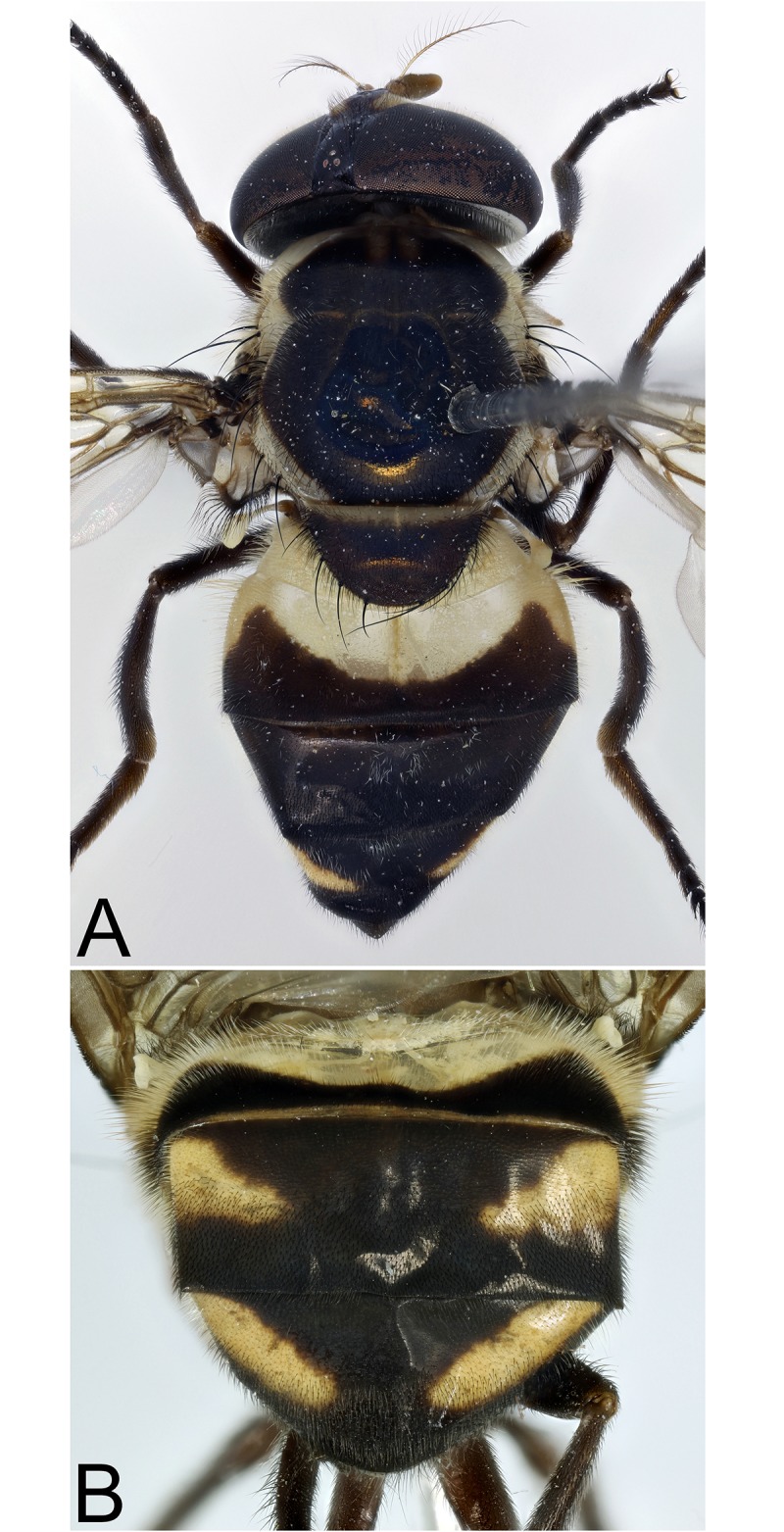
*Copestylum araceorum* sp. nov., dorsal view. A, Specimen from Costa Rica, female, whole body. B, Male holotype, abdomen.

*Abdomen*: widest at or near the apex of T2 and at this point, abdomen about a third again as wide as the scutum; rest of abdomen tapering to the apex of T5; abdomen variably coloured and marked, T1 and anterior half of T2 contrastingly white to yellow (Fig 11A); dorsum pilose, pile becoming longer towards lateral margins and not or only slightly longer on T4; pile variably coloured, usually matching the colour of underlying markings.

*Male genitalia*: cerci and surstylus of variable shape, the latter usually triangular in lateral view, sometimes subrectangular or L-shaped; epandrium with an upright projection at the point of articulation with the hypandrium; smooth margined superior lobe hook-like with variability in the outline shape and apex of the hook; aedeagal hood projecting ventrally.

**Fig 12:**
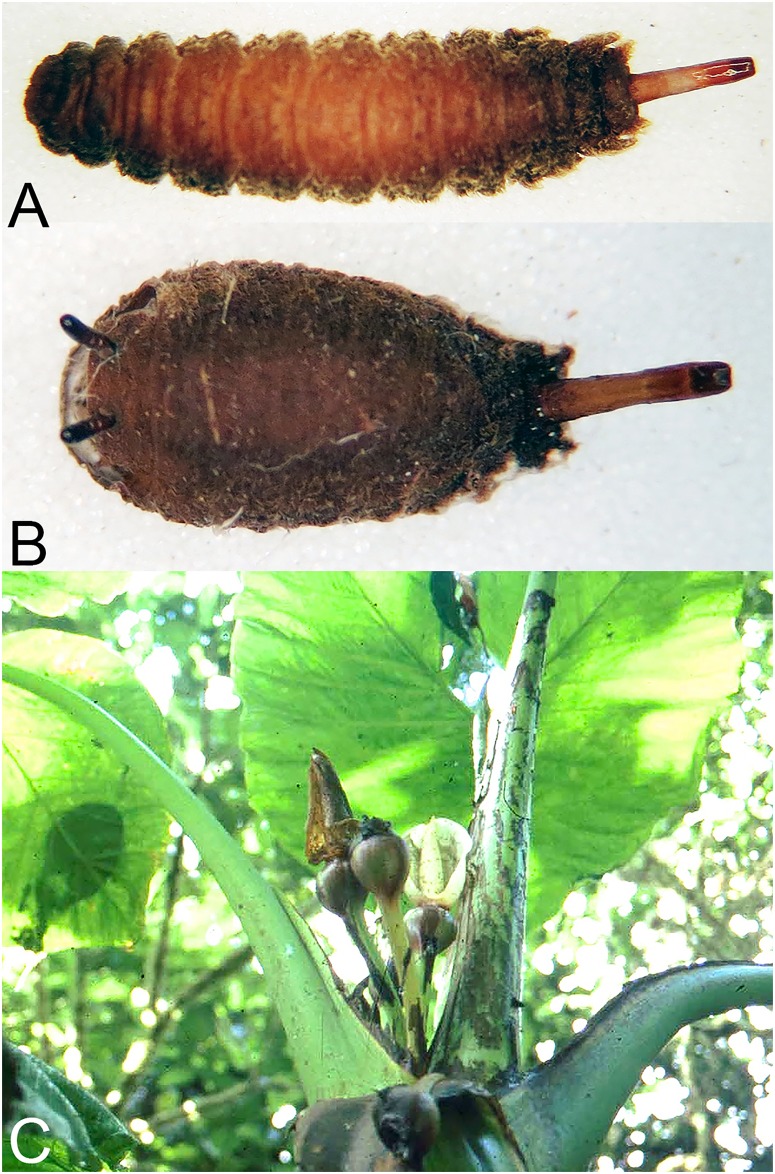
*Copestylum araceorum* sp. nov. A, Whole larva, head to the left. B, Whole puparium, head to the left. (30) Development site, *Anthurium* fruits (Araceae), Otonga Nature Reserve, Ecuador.

**Fig 13:**
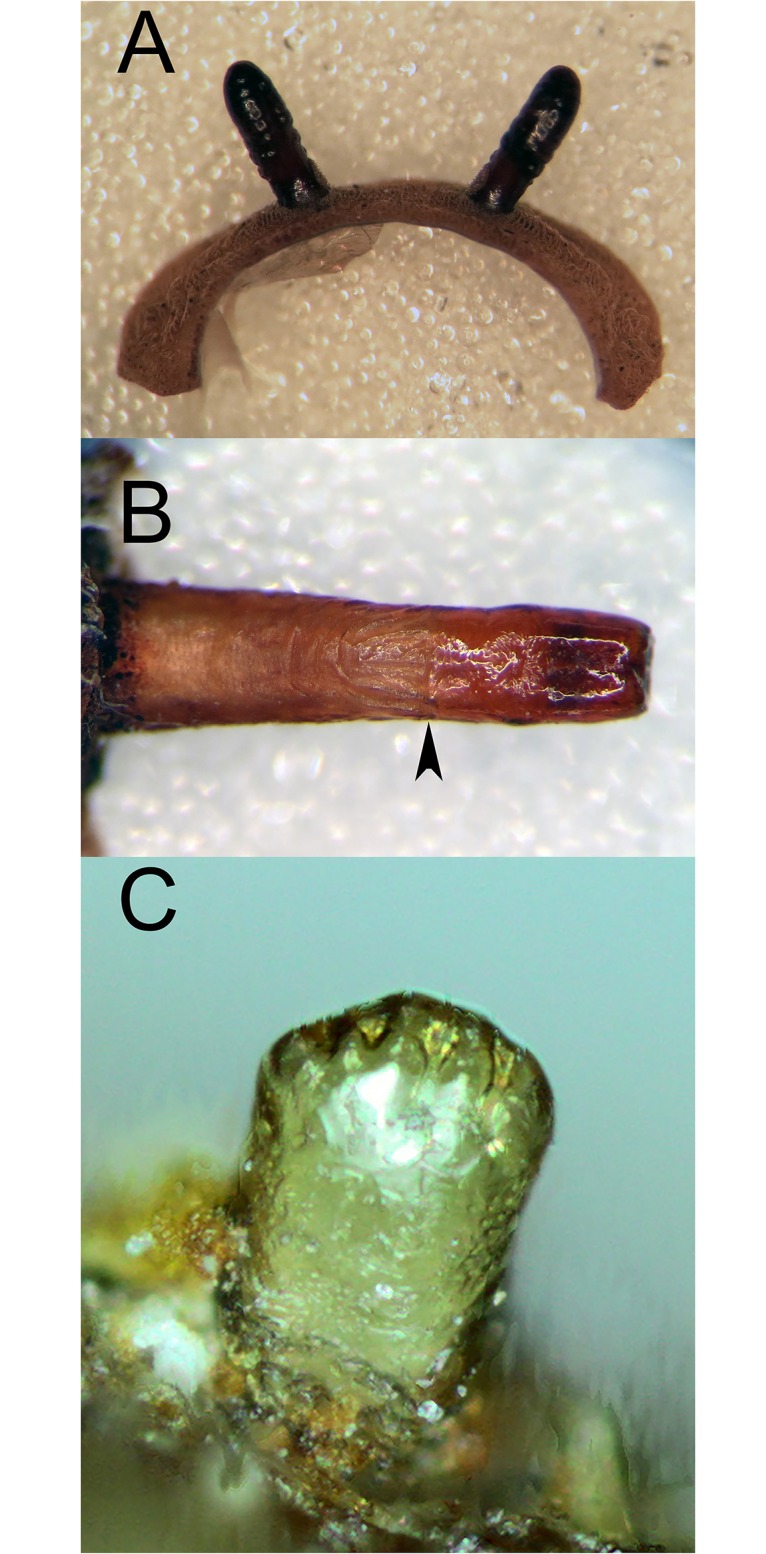
*Copestylum araceorum* sp. nov., holotype puparium. A, Pupal spiracles, posterior view. B, Posterior breathing tube, dorsal view (an arrowhead indicates position of the transverse ridge). C, Anterior breathing tube, lateral view.

**Fig 14:**
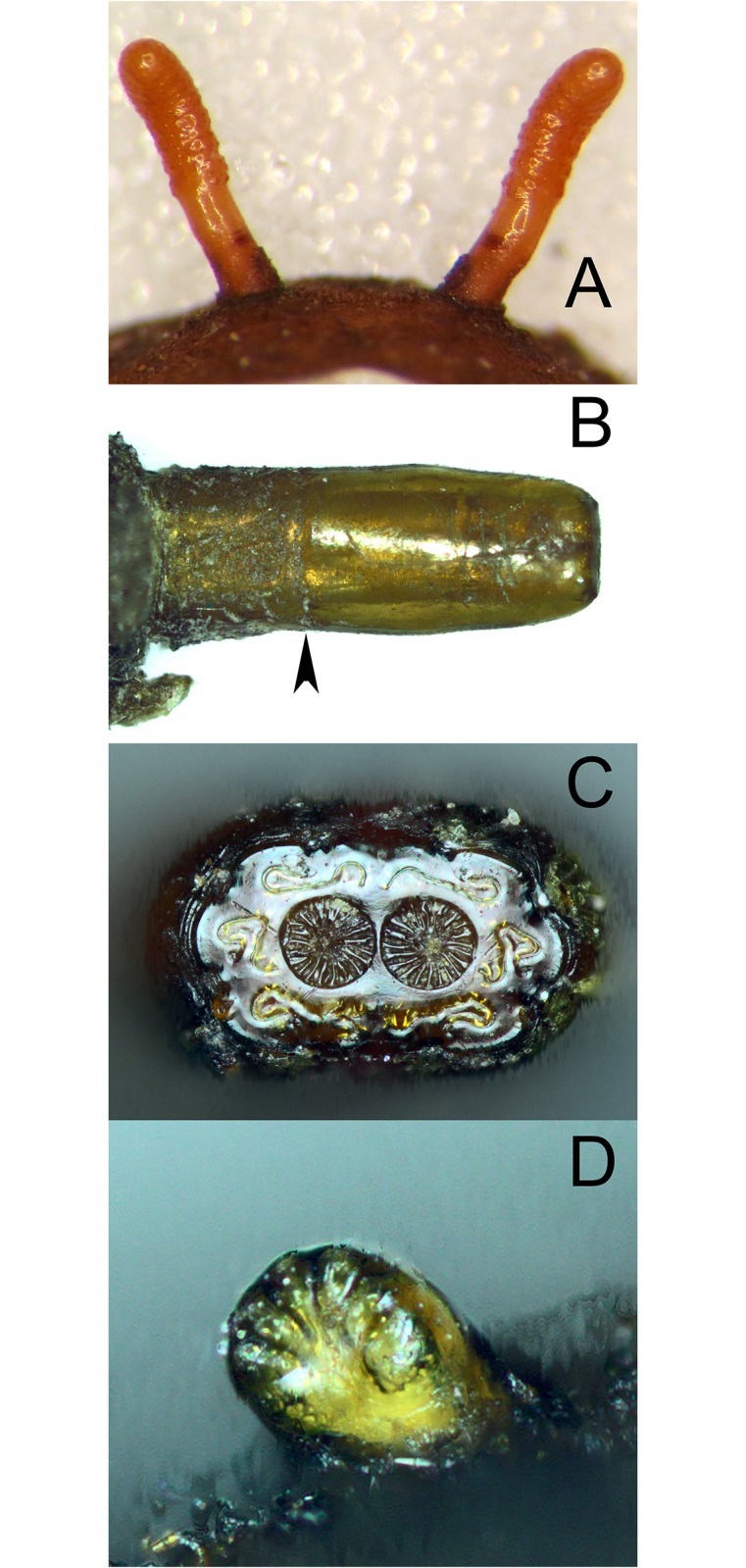
*Copestylum cinctiventre*, specimen from Trinidad. A, Pupal spiracles, posterior view. B, Posterior breathing tube, dorsal view (an arrowhead indicates position of the transverse ridge). C, Posterior breathing tube, apical view. D, Anterior breathing tube, lateral view.

**Fig 15:**
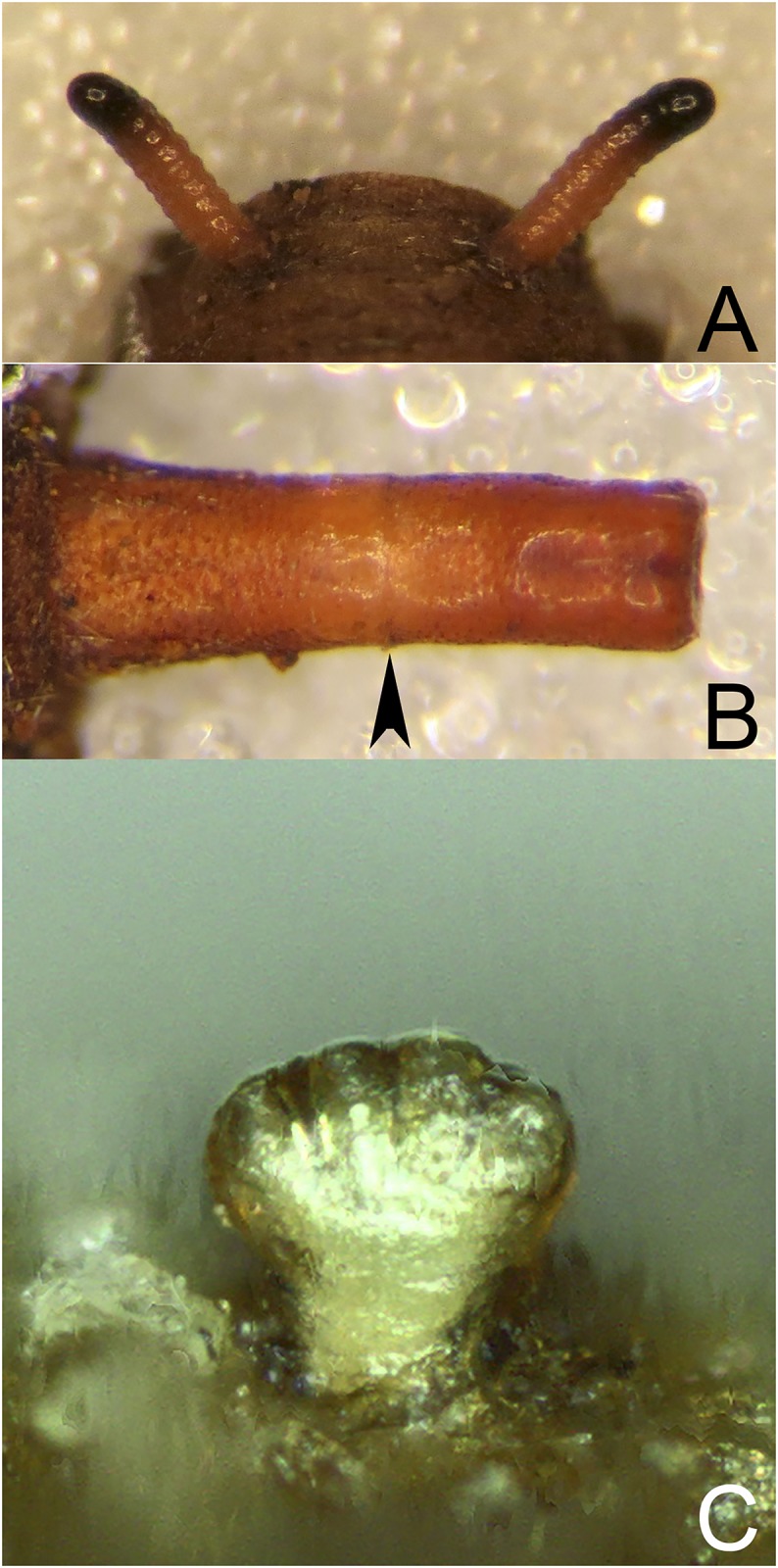
*Copestylum cyclops* sp. nov., holotype puparium. A, Pupal spiracles, posterior view. B, Posterior breathing tube, dorsal view (an arrowhead indicates position of the transverse ridge). C, Anterior breathing tube, lateral view.

*Early stages*: larva subcylindrical in cross-sectional shape; 12–15mm long, 3–5mm wide; truncate anteriorly, tapering posteriorly (Fig 12A); puparium drop-shaped (Fig 12B); projection bearing the antennomaxillary organs usually nodulate ventrally; lateral lips coated in fine setae; anterior fold of prothorax coated in transverse rows of backwardly directed spicules; anterior breathing tube wedge-shaped and tapering symmetrically each side with 4–6 spiracles across the apex (Figs 13C and 14B); antero-lateral margin of mesothorax with a group of spicules; mesothoracic prolegs with 2–3 transverse rows of 10+ crochets; antero-ventral margin of metathorax with two groups of linearly arranged spicules; dorsal and lateral margins coated in vestiture not in obvious transverse rows, vestiture longer on the lateral than dorsal margins and becoming longer towards the anal segment and longer than elsewhere on the anal segment; prolegs on abdominal segments 1–4 and rarely also on segments 5 and 6; prolegs usually with two rows of 6–8 crochets on abdominal segment 1 and reducing to 3–5 crochets on segment 4; posterior breathing tube varying in length from about half to twice the length of the anal segment; tube matt at base and shiny and variably punctate beyond transverse ridge; each spiracular plate with 3 pairs of curved spiracles, anterior and posterior spiracles S-shaped and longer than lateral spiracles which are also S-shaped, 4 groups of interspiracular setae, often broken off (Fig 14C); pupal spiracles yellow to brown and curved towards tip and above a matt base, with 6–11 bands of encircling spiracles, bands almost reaching the base (as in Fig 15A); bands coated in varying densities and lengths of pile; basal sclerite of head skeleton sclerotised black only at the dorsal bridge vertical plate and dorsal cornu; dorsal cornu short, under a third the length of the ventral cornu; labial bars and the mandibles and mandibular apodeme, narrow and sclerotised black; cibarial ridges present.

*Taxonomic notes*: adult stages are similar to those of the Chalybescens species group [5], but differ from them in lacking the defining feature of that group, a pair of oval-shaped, slightly depressed areas at the base of the scutellum bearing a rugose surface similar to that in the apical depression. Members of the Vagum group are also more pilose, with longer pile. Some Vagum group species are similar to certain Macula group (G.E. Rotheray, A. Ricarte & M.A. Marcos-García unpubl. data) species that have mostly shiny, lightly pollinose faces, but these species differ from the Vagum group in having a group of long, thick setae at the apex of the mid femora and thick, elongate setae in a row along the rear margin of the scutum. Larval and puparial stages are most similar to those of the Chalybescens group, sharing with them abdominal segments 5–7 lacking prolegs or if prolegs are present, they are smaller and have fewer crochets than on segments 1–4. A character that seems to separate some early stages of the two groups is the more developed groups of spicules on the antero-ventral margin of the metathorax in the Vagum group.

Adults of the Vagum group vary in colour patterns both between and within species. Sub-groups can be recognised on the basis of a shared pattern of colours, others on the basis of shared structures. A colour pattern sub-group is centred on *Copestylum vagum* (Wiedemann, 1830) which for all legs has coxae, trochanters and femora yellow to brown except for the femoral apices and the rest of the leg which is black and T3–4 yellow fasciate. A sub-group recognizable on morphological features is one based on *C*. *gertschi* (Curran, 1939), species of which have a narrow sulcus at the apex of the scutellum instead of a depression. Another is based on *C*. *sexmaculatum* (Palisot de Beauvois, 1819) and characterised by shiny, pellucid wings with areas clear of microtrichia.

***Copestylum araceorum*** Ricarte & Rotheray **sp. nov**. urn:lsid:zoobank.org:act: 6B5D21BC-E427-4183-951C-A2F5B5746101

Figs 1, 2, 3A, 4A, 5, 11A, 11B, 12A, 12B and 13A–13C

*Overall appearance*: a relatively large, dark species with strongly pigmented, black vittae on the centre and sides of the face, black legs and a conspicuous white marking at the base of the abdomen; male genitalia with globular cercus, evenly tapered surstylus and superior lobe not evenly curving from the base, curve leading to the hook longer so that the upper part of the hook is elongate and flattened.

*Adult size*: body length 9.7−11.7mm; wing length 10.8−11.5mm (n = 4)

Male holotype

*Head*: face with a strong (pigmented completely), black central vitta (Fig 3A) and a similarly strong pair of side vittae (Fig 4A); eyes rounded and orange pilose, except pile short and scattered on the lower quarter of eye; lunule black; scape and pedicel black bristled; basoflagellomere brownish.

*Thorax*: scutum black (Fig 11A), with opalescent reflections (more evident under artificial light), except for a yellow lateral margin from the postpronotum to the postalar callus and two medial fasciae ending before the transverse suture; scutum with black and yellow pile of different lengths intermixed and pile conspicuously longer at the rear margin; lateral setae black: 3 above wing insertion and 3 on the postalar callus; pleuron yellow except for a continuous U-shaped black mark on the lower half encompassing the katepisternum, meron, lower part of katepimeron, metasternum and anterior part of posterior anepisternum (Fig 5); posterior anepisternum with a black seta postero-dorsally; pleuron with yellow pile, except for some black pile on the ventral part of katepisternum; scutellum with pre-apical depression; scutellum yellow round the margin, otherwise shiny and metallic; except for pale pile at base, scutellum with black pile of variable size, including some very long pile (> half length of the apical bristles) and conspicuous black setae round the margin, 4 on one side, 3 on the other; legs dark brown to black and pile extensively black; wing very slightly brown pigmented on the anterior half (stigma darker); wing microtrichose except for parts of cells BM, CuP, CuA1 and anal lobe; halter yellow; calypter light brown, except for the dark brown margin and fringe.

*Abdomen*: T1 white and with yellow pile (Fig 11A); T2 black except for a crescent-shaped, white band on the anterior margin and lateral margins yellow; T2 with black and yellow pile overlying the black and white markings respectively; T3&4 black, except for pairs of antero-lateral, oblique yellow bands (Fig 11B); anterior halves of T3&4 with white pile except some black pile medially, posterior half with black pile; S1&2 yellow and yellow haired; S3&4 extensively black and with mostly yellow pile.

*Genitalia*: cercus globular in lateral view; surstylus with evenly tapered apex; hook-shaped superior lobe with the part of the curve leading to the apex longer than the basal part so that the apical part of the hook appears elongate and flattened; extreme apex tapered abruptly (Fig 2).

*Female*: same as the male except for the following characters: vertex and frons black, the lower third of frons black only medially; frons excavated in the central section; vertex and frons extensively dark brown to black haired; basoflagellomere dark brown; pile on the scutum shorter than that in the male; T3 with two small, faint, yellow maculae antero-laterally (Fig 11A); T5 black.

*Early stages*: third stage larva with vestiture long and dense on the anal segment (Fig 12A), puparium drop-shaped (Fig 12B); pupal spiracles short, shorter than distance apart and black marked (Fig 13A); pile of pupal spiracles inconspicuous; posterior breathing tube with transverse ridge just above the middle of the length of the tube, tapered, not squared-off at apex, mostly smooth and shining, without obvious punctures above the transverse ridge (Fig 13B); spiracular plate with relatively convoluted spiracles (as in Fig 14C); prolegs with crochets on abdominal segments 1–6, reducing in size and number of crochets towards segment 6.

*Material examined*: *holotype*: 1♂ with puparium and genitalia dissected, COSTA RICA, Cartago, P.N. (‘National Park’) Tapantí, ex Araceae spathe, 8.vii.2000, leg. G.E. Rotheray [INBio]. *Paratypes*: 2♂ and 1♀ with puparia and 2 third stage larvae, COSTA RICA, Cartago, P.N. Tapantí, ex Araceae spathe, L: 8.vii.2000, leg. G.E. Rotheray; 2♂ reared from larvae, Cartago, P.N. Tapantí, ex Araceae spathes, L: 8.vii.2000, A: 6.viii.2000; 1♀ with puparium, Cartago, P.N. Tapantí, ex Araceae spathe, L: 8.vii.2000, A: 9.viii.2000 [NMS & CEUA]; 1♂ with puparium and genitalia dissected, ECUADOR, Otonga, S. of Quito, 23.viii.2000, cloud forest, ex Araceae spathe, leg. G.E. Rotheray and E.G. Hancock [HM].

*Etymology*: the epithet ‘*araceorum*’ meaning ‘from Araceae plants’ refers to the development site of this species (Fig 12C).

*Taxonomic notes*: the adult of this species differs from all other Vagum group species considered here by its all black legs. Other species have yellow or orange marked legs. *Copestylum araceorum* is also darker in overall colour pattern than other species and has fully pigmented, shiny black, facial vittae. Early stages are distinguished from all other species of the Vagum group by the long, thick and dense vestiture that disguises the lappets on the anal segment and the pupal spiracles which are short, being only about as long as their distance apart. *Copestylum araceorum* is most similar to *C*. *willistoni* and *C*. *cyclops* in having a central vitta on the face and in the early stages it is most similar to *C*. *willistoni* in having prolegs with crochets on the first 6 abdominal segments. In *C*. *cyclops* and other Vagum group species, prolegs with crochets are absent on segment 6. Apart from short pupal spiracles, *C*. *araceorum* is readily distinguished from *C*. *willistoni* by the apex of the breathing tube which tapers towards the apex and is smooth, shining and lacks punctures, punctured in *C*. *willistoni*.

*Biology*: adults reared from spathes of more than one Araceae species. Species only known from Costa Rica and Ecuador.

***Copestylum cyclops*** Ricarte & Hancock **sp. nov**. urn:lsid:zoobank.org:act: 71957709-8B06-472D-BDEE-8BA7A80CB5EA

**Fig 16:**
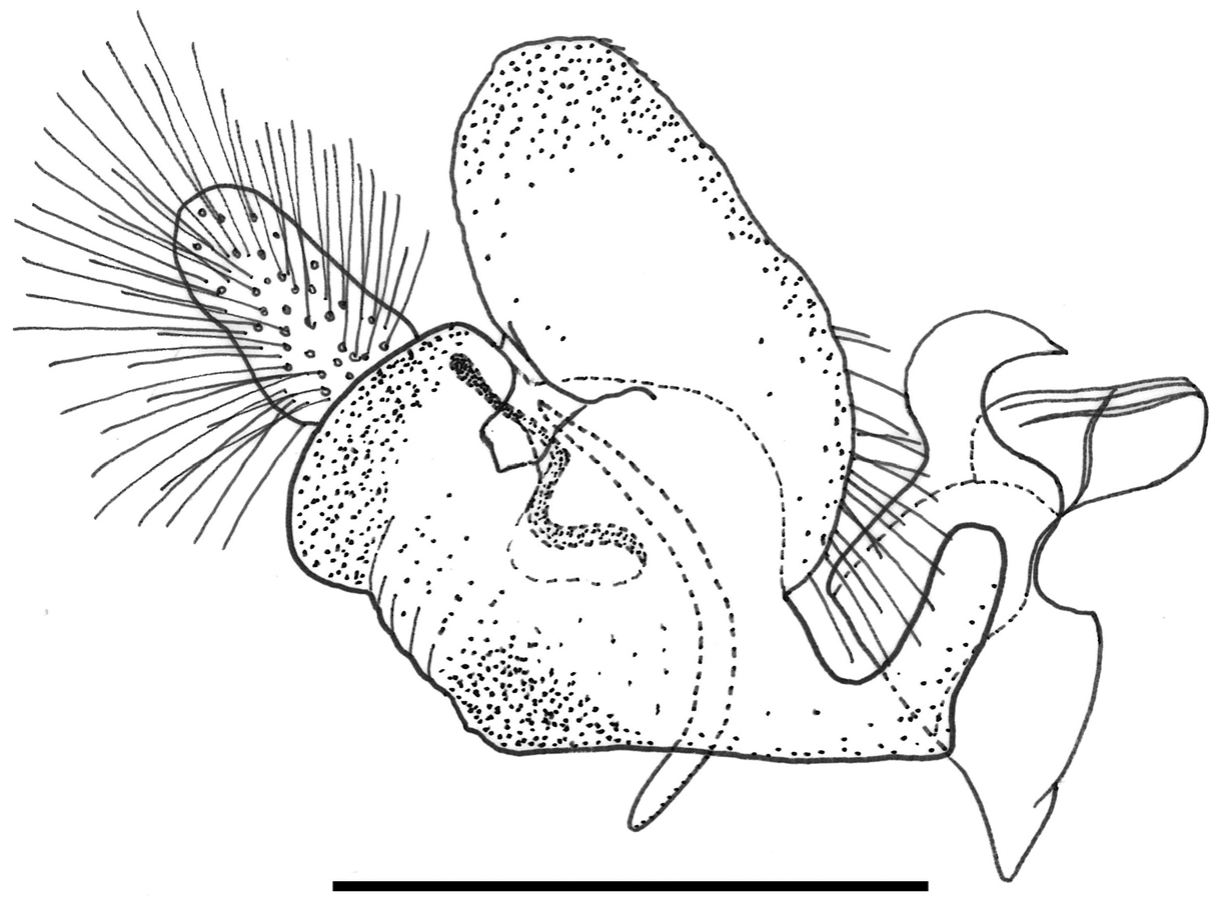
*Copestylum cyclops* sp. nov. Male holotype, genitalia, lateral view. Scale line = 0.5mm.

Figs 4B, 6, 15A–15C and 16

*Overall appearance*: a yellow-orange species usually with a black, central vitta on the face and a black prescutellar macula on the scutum; legs orange from base to femoral apices, rest darkened and front and middle legs with yellow to orange pile, rest darkened. Male genitalia with elongate cercus, about 2× as long as basally wide; surstylus subrectangular and evenly rounded apically; superior lobe in the form of an evenly rounded hook.

*Adult size*: body length 6.7−11.2mm; wing length 6.7−8.8 (n = 6)

Male holotype

*Head*: face with a central pale brown vitta (Fig 3B); eyes elongate and yellow pilose, except pile almost completely absent on lower third (Fig 4B); lunule shiny orange; scape and pedicel with both black and yellow setae.

*Thorax*: scutum shiny orange, with a brown prescutellar blotch and two brown medial stripes ending before the transverse suture; scutum with black pile, except for a band of yellow pile starting just anterior to the transverse suture and ending about the middle; lateral setae orange: 2 setae above wing insertion and 2 on postalar callus; pleuron yellow and pile yellow, with a yellow seta on posterior anepisternum postero-dorsally (Fig 6); scutellum yellow round the margin, otherwise shiny and metallic; scutellum with black pile of variable size and density, longest pile less than half the length of the apical setae; marginal setae yellow, apical pair thicker and longer than the less conspicuous setae on the lateral margin; legs yellow with blackish tibiae and tarsi; except for yellow pile at base of femora, legs with pile extensively black (Fig 6); wing lightly sepia anteriorly, with a conspicuous dark brown pterostigma; wing extensively bare basally and centrally, alula wholly microtrichose; halter with pedicel yellow and capitulum white; calypter black.

*Abdomen*: T1 yellow and with yellow pile; T2 yellow except for black posterior margin and with yellow pile except black on posterior and lateral margins; T3 with a yellow band in the anterior two thirds and T4 with a yellow band in the anterior half, otherwise black, pile black; S1&2 yellow and with yellow pile; S3&4 dark yellow and some pile black laterally, otherwise yellow.

*Genitalia*: cercus elongate, about 2× as long as basally wide; surstylus subrectangular with a rounded apex; hook-shaped superior lobe with a smooth, even curve (Fig 16).

*Female*: similar to the male except for the following characters: frons usually darkened, occasionally only posteriorly or wholly yellow; frons with dark brown to black pile, yellow anteriorly; eye pile shorter than in male and ventral half of eye virtually bare; postalar callus with 2–3 setae.

*Early stages*: pupal spiracles long, longer than distance apart (Fig 15A), oval not circular in cross-sectional shape, pile inconspicuous; posterior breathing tube short, transverse ridge at, not clearly above, the mid-point, tube parallel-sided at apex, not tapering and with punctures above the transverse ridge (Fig 15B); anterior breathing tube with 4 spiracles across the symmetrical apex (Fig 15C); prolegs on abdominal segments 1–4, reducing in size and number of crochets from segment 1 to 4.

*Material examined*: *holotype*: 1♂ with puparium and genitalia dissected, COSTA RICA, Guanacaste, Caribe, Est. (‘Station’) El Pilón, P.N. Volcán Tenorio, 400m S, path to waterfall, 700m, under decaying bark of a dead, standing unidentified shrub, L: 8.i.2007, A: 14.ii.2007, leg. J.A. Azofeifa [INBio]. *Paratypes*: COSTA RICA: 1♂ and 1♀ with puparia, Alajuela, Upala, PN (‘National Park’) Tenorio, Sector El Pilón, 800m, LN 298380–427850, ex decaying Sapotaceae fruit, L: 2.viii.2011, P: 28.viii.2011, A: 8.ix.2011, leg. A. Azofeifa; 1♂ (CRI001302352), Est. Eladios, 820m, Ref. (‘Refuge’) Peñas Blancas, Res. Biol. (‘Biological Reserve’) Monteverde, Prov. Alaju. (‘Alajuela Province’), L-N-254750 457650, 2−5.x.1990, leg. E. Bello, det. as *CR28*; 1♂, without locality data; 1♂ (CRI002475824), Prov. Puntarenas, Est. Agujas, Sendero Ajo, 300m, L_S_276750_526550, 24−26.ix.1996, de luz (‘light trap’), leg. A. Azofeifa, #8489; 1♂ with puparium, Guanacaste, Caribe, Falda N Volcán Tenorio, Valle río Roble, sendero a cerro Montezuma, 850m, ex fallen fruit of *Pachira acuatica* (Malvaceae), L: 11.iii.2007, A: 25.iv.2007, leg. J.A. Azofeifa; 3♂ with puparia, Guanacaste, Caribe, Estación El Pilón, P.N. Volcán Tenorio, 400 m S, path to waterfall, 700m, L: 8.i.2007, A: 12.ii.2007 (2♂), A: 15.ii.2007 (1♂), under decaying bark of a dead, standing shrub, leg. J.A. Azofeifa; 1♂ (CRI001048332), Estac. (‘Station’) Pitilla, 700m, 9km, S. Santa Cecilia, Guanac. Pr. (‘Guanacaste Province’), GNP Biodiversity Survey, W85°25′40″ N10°59′26″, xi.1988; 1♀ (CRI000136654), Estac. Pitilla, 700m, 9km, S. Santa Cecilia, Guanac. Pr., GNP Biodiversity Survey, 330200, 380200, 21.iii−21.iv.1989; 1♀ with puparium, JAAZ-1989.31 (code information not found); 1♀, Volcán Tenorio, Alajuela, Upala, Albergue Heliconias, 30.i.2006, trampa luz (‘light trap’), leg. M.A. Marcos & G. Rotheray (10° 42′ 45.21″ N 85° 1′ 41.15″ W, 900 m asl); 1♀ (INB0003027826), Prov. Alajuela, A.C.A., San Carlos, Reserva Ftal (‘Forestal’) Arenal, Sector la Península, 600m, ii.1999, leg. G. Carballo; 1♀ (INB0003056003), Puerto Jiménez (Osa, Puntarenas), Estación los Patos, ex Sapotaceae fruit, L: 25.v.1999, A: 18.vi.1999, leg. M.L.V.; 1♀ (deformed) with puparium, Guanacaste, Caribe, Est. El Pilón, P.N. Volcán Tenorio, 400m S, path to waterfall, 700m, under bark of a dead standing shrub, L: 8.i.2007, A: 15.ii.2007, leg. J.A. Azofeifa. All male paratypes with genitalia dissected [INBio, NMS & CEUA].

*Etymology*: The Latin epithet ‘*cyclops*’ refers to the one-eyed giant, Cyclops, in Greek mythology and is given to this species because of extensive eye contiguity which gives ‘one-eyed‘ appearance to this syrphid.

*Taxonomic notes*: *C*. *cyclops* varies in colour. On the face, the central vitta can be faint, reduced or even absent. The scutum ranges from yellow to dark orange and can also be wholly pale. The dorsum of T3 and 4 ranges from extensively light brown (with posterior margins of terga black) to jet black with lateral faded spots. *C*. *cyclops* is, nonetheless, distinguished from most Vagum group species by the presence of a central, black vitta on the face, a character shared only with *C*. *willistoni* and *C*. *araceorum*. It can be distinguished from these latter two species by the pile of the front and mid femora which in *C*. *cyclops* is yellow to orange in the basal third; in contrast these femora are entirely coated in black pile in *C*. *willistoni* and *C*. *araceorum*. If the facial vitta is faint, the yellow to orange pile in the basal third of the mid-femora separates *C*. *cyclops* and *C*. *musicanum* from all other Vagum group species lacking a central vitta. *C*. *cyclops* can be distinguished from *C*. *musicanum* by the shape of the eyes which are dorsally flattened (Fig 4B). The early stages of *C*. *cyclops* differs from others of the Vagum group by the relatively short, posterior breathing tube with the transverse ridge at about the mid-point of the length of the tube and the obvious, deep punctures above the transverse ridge. Of the four Vagum group species studied here, a short posterior breathing tube with the ridge at about the mid-point is shared only with *C*. *tenorium*, but the tube is lightly and inconspicuously punctured in that species. The pupal spiracles of these two species have bands that are interrupted on both dorsal and ventral surfaces with a narrow clear strip between the bands. In *C*. *cyclops*, the dorsal strip is wider and complete to the apex, in *C*. *tenorium* the cleared strip is narrower and the top two bands are complete and not interrupted.

*Biology*: adults were reared most frequently from decaying fruits of species of Malvaceae and Sapotaceae and also from under decaying bark of an unidentified shrub. This species is only known from Costa Rica.

***Copestylum musicanum*** (Curran, 1930)

Figs 17 and 18.

**Fig 17:**
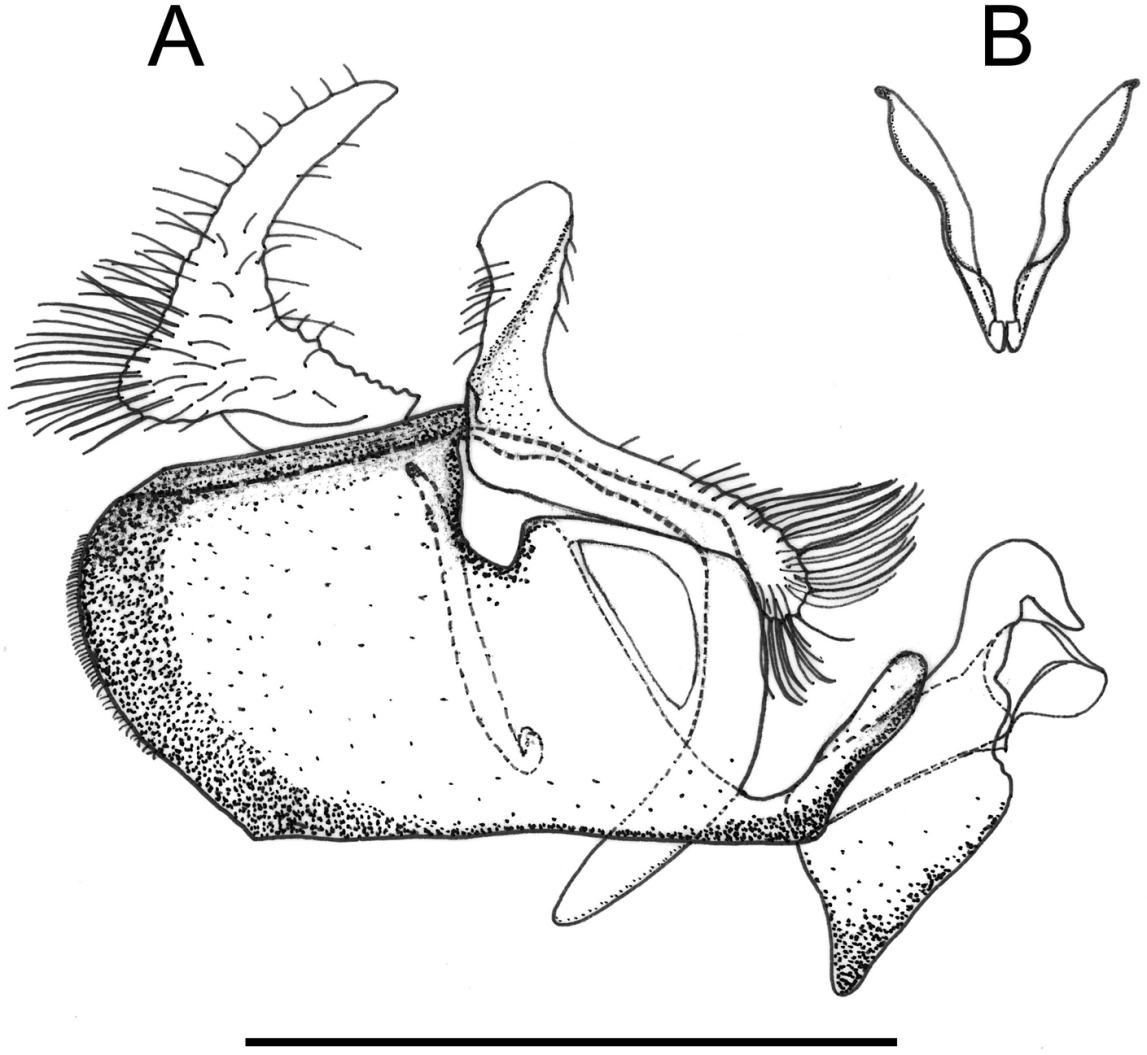
*Copestylum musicanum*, male holotype, genitalia, scale line = 1mm. A, Whole genitalia, lateral view. B, Surstylar apodeme, ventral view.

**Fig 18:**
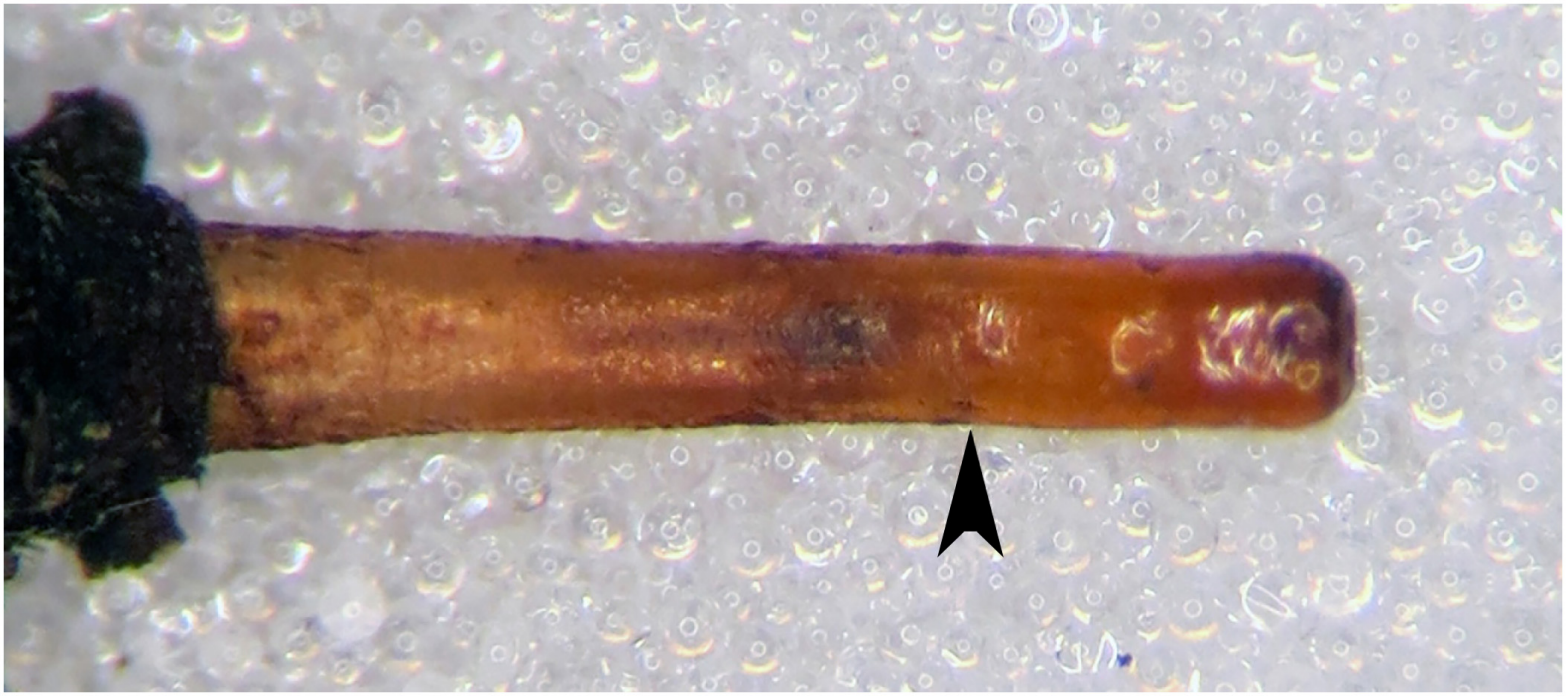
*Copestylum musicanum*. Specimen from Costa Rica, posterior breathing tube, dorsal view (an arrowhead indicates position of the transverse ridge).

*Overall appearance*: a yellow-orange species lacking a central vitta on the face and with a black-marked scutum, base of legs yellow and rest of legs dark and distinctive male genitalia with both cercus and surstylus L-shaped.

*Diagnostic features*: face without a central vitta (Fig 3D); eyes elongate and yellow pilose; lunule yellow; scape and pedicel yellow setulose; scutum orange except for yellow anterior and lateral margins and brown prescutellar macula and two brown, medial vittae ending before the transverse suture; scutum with black pile anteriorly, yellow pile starting just anterior to the transverse suture and yellow and black pile admixed along the rear margin; lateral setae: 3 above wing insertion and 3 on postalar callus; lower half of pleuron orange, upper half yellow except slightly darkened katatergum, katepimeron and meron and with yellow pile and a yellow seta on the posterior anepisternum; scutellum yellow except for black apical margin and slightly darkened latero-apical margins; coxae, trochanters and base of femora orange and with orange pile, otherwise legs darkened with black pile although basotarsomere of meso- and metalegs contrastingly pale, although black pilose; wing with cells R, BM and CuP extensively bare of microtrichia; cells R2+3, R4+5, DM and CuA1 bare basally; alula wholly microtrichose; wing with a conspicuous, black, pterostigmal mark, mostly on cell SC but extending to the apex of cell C; T1 yellow and with yellow pile; T2 yellow except for black posterior margin and with yellow pile except black on posterior margin and apical end of lateral margins; T3&4 black except for a pair of comma-shaped yellow fasciae whose narrow ends almost meet on the midline of the posterior margin of each tergum and pile all black on T3 and all white on T4; S1&2 yellow and yellow pilose; S3&4 dark yellow and some pile black laterally, otherwise yellow; male genitalia: in lateral view, cercus and surstylus L-shaped, with basal section of surstylus longer than the apical section (Fig 17A); surstylar apodeme not conspicuously curved upwards at base, nor expanded apically and apex not developed, without an elongate, sclerotised apical extension (Fig 17B).

*Early stages*: pupal spiracles long, longer than distance apart, oval not circular in cross-sectional shape, pile inconspicuous; posterior breathing tube long, transverse ridge clearly above the mid-point of the length of the tube and parallel-sided at apex, not tapering and mostly smooth and shining above the transverse ridge, any punctures light and inconspicuous (Fig 18); prolegs on abdominal segments 1–4, reducing in size and number of crochets from segment 1 to 4.

*Material examined*: *holotype*: 1♂ in good condition, with genitalia dissected, Type *Volucella musicana* Curran ♂ (written on a red label) / S.W. Williston Collection / Chapada / Am. Mus. Nat. Hist., Dept. Invert. Zool., No. 19224 [AMNH]. *Additional material*: COSTA RICA: 1♂ and 1♀ with puparia, Guanacaste, Rincón de la Vieja, Estación San Gerardo, ex decaying fruit of *Crescentia cujete* (Bignoniaceae), L: 15.viii.2001, A: A: 1.x.2001 (♂), A: 16.x.2001 (♀), leg. G.E. Rotheray; 1 ♂ with puparium (INB0003055985), Guanacaste, Santa Rosa, Tempisque, en el cruce antes del Mirador (‘at the crossroad before the Mirador’), ex fruit of *Theobroma cacao* (Malvaceae), P: 28.vii.1999, A: 18.viii.1999, leg. A Picado; 1♂ and ♀ with puparia, Alajuela, Guatuso, Cote, Fca. (‘estate’) Justo Robles, 494m, N10 35 36.6 W 84 51 40.8, ex decaying palm stump, L: 13.iv.2010, various collectors; 1♂ with puparium, Alajuela, Guatuso, Buenavista, Fca. La Garroba, 93m, N10 48 11.7 W 84 53 23.2, ex unidentified Sapotaceae fruit, L: 13.vii.2010, P: 17.viii.2010, A: 20.viii.2010, various collectors; 1♂ with puparium, Alajuela, Guatuso, Buenavista, Fca. La Garroba, 93m, N10 48 11.7 W 84 53 23.2, ex Sapotaceae fruit, L: P: 15.viii.2010, A: 27.viii.2010, various collectors; 4♀ (INB0003055982, INB0003055982 and INB0003055983), Limón, R.B. (‘Biological Station’) Hitoy Cerere, ex fruits of *Pouteria viridis* (Sapotaceae), L: 24.iv.1998 and 16.ix.1998, A: 14−16.x.1998, leg. E. Rojas; 1♀ with puparium, Alajuela, Guatuso, Buenavista, Fca. La Garroba, 93m, N10 48 11.7 W 84 53 23.2, ex Sapotaceae fruit, L: 13.viii.2010, A: 25.viii.2010, various collectors [INBio, NMS, CEUA].

**Fig 19:**
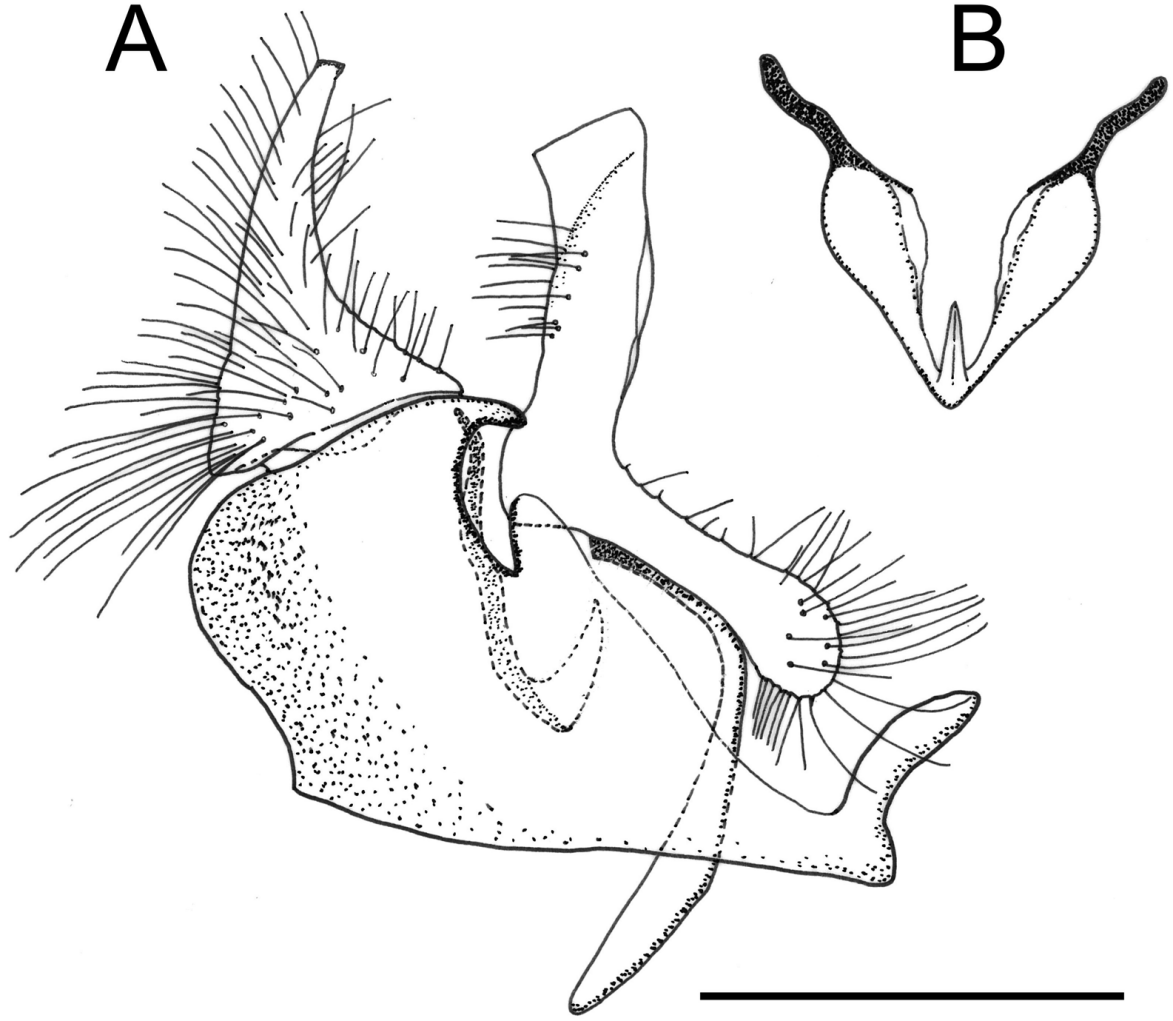
*Copestylum tigrinum* sp. nov., male holotype, genitalia, scale line = 0.5mm. A, Whole genitalia, lateral view. B, Surstylar apodeme, ventral view.

*Taxonomic notes*: the adult stage of *C*. *musicanum* is distinguished from other Vagum group adults by colour of the legs which have the coxae and trochanters and extreme bases of the femora yellow and the rest of the legs darkened. The male genitalia are also distinctive in having, in lateral view, a L-shaped cercus and surstylus. The male genitalia of *C*. *trigrinum* also have a L-shaped surstylus, but the cercus is triangular in profile shape among other differences (Figs 17A, 17B, 19A and 19B). The early stages of *C*. *musicanum* are most similar to those of *C*. *vagum*. They both have pupal spiracles longer than their distance apart and long posterior breathing tubes, about or more than the maximum width of the larval or puparial body with the transverse ridge well above the middle of the tube. The early stages of *C*. *musicanum* can be distinguished from those of *C*. *vagum* by the apex of the posterior breathing tube above the transverse ridge which is mostly smooth and shining, this part more frequently and more deeply punctured in *C*. *vagum*.

*Biology*: adults were reared in Costa Rica from decaying fruits of species of Bignoniaceae, Malvaceae and Sapotaceae and a decaying palm stem (Palmaceae).

***Copestylum tenorium*** Ricarte & Rotheray **sp. nov**. urn:lsid:zoobank.org:act: 3C7E16F1-3D37-424B-AEA5-73DB857D9AEA

**Fig 20:**
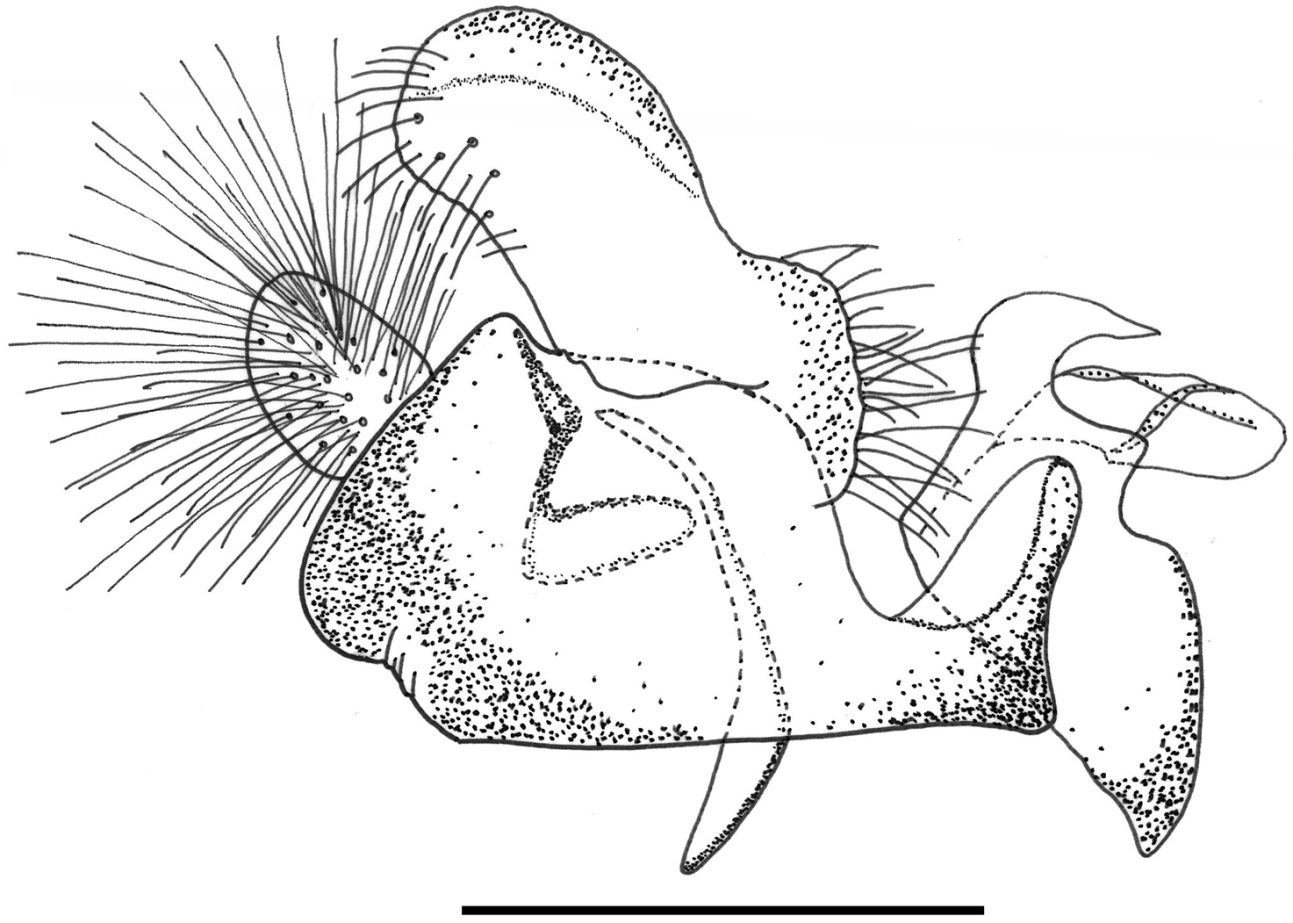
*Copestylum tenorium* sp. nov. Male holotype, genitalia, lateral view. Scale line = 0.5mm.

Figs 3C, 4C, 7 and 20

*Overall appearance*: a yellow to orange species with T2–4 greenish with narrowly black posterior margins, pleuron without black maculae, face without a central vitta and the top of the male eyes flattened. Male genitalia with subrectangular surstylus abruptly indented ventrally, just below the middle.

*Adult size*: length (mm): body = 8.3−10, wing = 8.2−10.2 (n = 4)

Male holotype

*Head*: face greenish-yellow without a clear central vitta (Fig 3C); eyes rounded and yellow pilose, except pile almost completely absent on lower half (Fig 4C); lunule contrastingly darkened compared to the face; scape and pedicel with yellow bristles.

*Thorax*: scutum dark greenish-orange with a central, brown, prescutellar macula and similar coloured maculae on the scutum; scutum with pile not much longer at the rear margin; pile black except for a band of orange pile starting just anterior to the transverse suture and ending at the brown blotch on the rear margin; lateral setae orange: 4 above wing insertion and 2 on postalar callus; pleuron dark orange in the lower half, lighter orange above and with orange pile and an orange seta on posterior anepisternum (Fig 7); scutellum dark greenish-orange except for black apex; scutellum with black pile except for orange pile along the ventral margin especially at the base; marginal setae orange, apical pair thicker and longer than the less conspicuous setae on the lateral margin; legs with coxa and trochanter dark orange, rest of legs greenish-orange, except extreme apex of metafemora black; coxa, trochanter and femur with orange pile, except postero-apical end of femur and anterior face of metafemora with black pile; tibia and tarsi with black pile; wing lightly sepia anteriorly, extensively bare basally and centrally and a conspicuous dark brown pterostigma; alula wholly microtrichose; halter with pedicel yellow and capitulum white; calypter black.

*Abdomen*: T1 light greenish-orange and with yellow pile; T2–4 greenish-orange except for a black apex; T2 with yellow pile anteriorly, black over the lateral margin and extending halfway along the lateral margins; T3&4 with black pile; S1–4 greenish-orange with slightly darkened posterior margins and entirely with yellow pile.

*Genitalia*: cercus tapered and about as long as basally wide; subrectangular surstylus abruptly indented just below the middle; superior lobe hook-shaped and curved unevenly with a longer section above the base and tapering to a point (Fig 20).

*Female*: similar to the male except for the following characters: frons yellow with subtle green reflections, black on the ocellar triangle and behind it; frons yellow pilose and, on the posterior half, dark brown pile intermixed; eye pile shorter than in male; ventral half of eye virtually bare; subscutellar fringe of black or yellow hairs.

*Early stages*: pupal spiracles long, longer than distance apart, oval not circular in cross-sectional shape, pile inconspicuous; bands bearing spiracles interrupted on both dorsal and ventral margins by a clear strip; posterior breathing tube short, transverse ridge at, not clearly above, the middle, tube parallel-sided at apex, not tapering and lightly and inconspicuously punctured above the transverse ridge; prolegs on abdominal segments 1–4, reducing in size and number of crochets from segment 1 to 4.

*Material examined*: *holotype*: 1♂ with genitalia dissected, COSTA RICA, Pilón, 13-En 11 (‘13.i.2011’), libre (‘free’ sampling), J. Azofeifa [INBio]. *Paratypes*: COSTA RICA: 1♂ (INBIO CRI000623758), Est. Cacao, 1000–1400m, lado SO Vol. (‘Volcano’) Cacao, P.N.G. (‘Guanacaste National Park’) Prov. Guan., C. Chaves, L-N-323300, 375700, vi.1991; 1♂ with puparium, Alajuela, Upala, PN Tenorio, Sector El Pilón, 800m, LN 298380–427850, ex decaying ‘inga’ fruit [*Inga edulis* Mart. (Fabaceae)], L: 4.ix.2010, P: 16.x.2010, A: 30.x.2010, various collectors; 1♀ with puparium (INB0003056004), Alajuela, San Carlos, Fortuna, Est. Península, ex decaying fruit, L: 30.x.1999, leg. J.D. Gutiérrez; 1♀ with puparium, Alajuela, Upala, PN Tenorio, Sector El Pilón, 800m, LN 298380–427850, ex Sapotaceae fruit, L: 4.ix.2010, various collectors; 1♀ with puparium, Alajuela, Upala, PN Tenorio, Sector El Pilón, 800m, LN 298380–427850, ex bromeliad, L: 13.v.2010, P: 14.viii.2010, A: 28.viii.2010, various collectors; 1♀ with puparium, Alajuela, Guatuso, Cote, Fca. Justo Robles, 494m, N10 35 36.6 W 84 51 40.8, ex decaying palm stump, L: 13.iv.2010, various collectors; 1♀ with puparium (INB0003056019), Limón. Res. Biol. (‘Biological Station’) Hitoy Cerere, ex Sapotaceae fruit, L: 10.12.1998, A: 18.i.1999, leg. E. Rojas. All male paratypes with genitalia dissected [INBio, NMS, CEUA].

*Etymology*: the epithet *tenorium* refers to the Tenorio Volcano National Park, the type locality of this species.

*Taxonomic notes*: males are generally darker than females. Among the Vagum group species examined here, *C*. *tenorium* is most similar to *C*. *vagum* in having greenish T2–4 with narrow, black, apical margins that lack yellow spots and marks. *C*. *tenorium* is readily distinguished from *C*. *vagum* by the completely yellow to orange pleuron which in *C*. *vagum* is black marked. Males of these two species are readily separated in that only *C*. *tenorium* has the dorsal margin of the eyes flattened and the indented surstylus is unique among all Vagum group species examined here. The early stages of *C*. *tenorium* are most similar to *C*. *cyclops*. Both species have a relatively short posterior breathing tube, less than larval or puparial width and with the transverse ridge at about the mid-point of the length of the tube. *C*. *tenorium* is readily distinguished from *C*. *cyclops* by the pupal spiracles which are oval in cross sectional shape with the bands bearing spiracles interrupted on both dorsal and ventral margins by a clear strip and the posterior breathing tube which is lightly and inconspicuously punctured.

*Biology*: adults were reared from decaying fruits of species of Fabaceae and Sapotaceae and also from an unidentified bromeliad (Bromeliaceae) and a decaying palm stump (Palmaceae). This species is only known from Costa Rica.

***Copestylum tigrinum*** Ricarte & Hancock **sp. nov**. urn:lsid:zoobank.org:act: D22B7C3D-180B-4D24-B293-3A10AC7E6938

**Fig 21:**
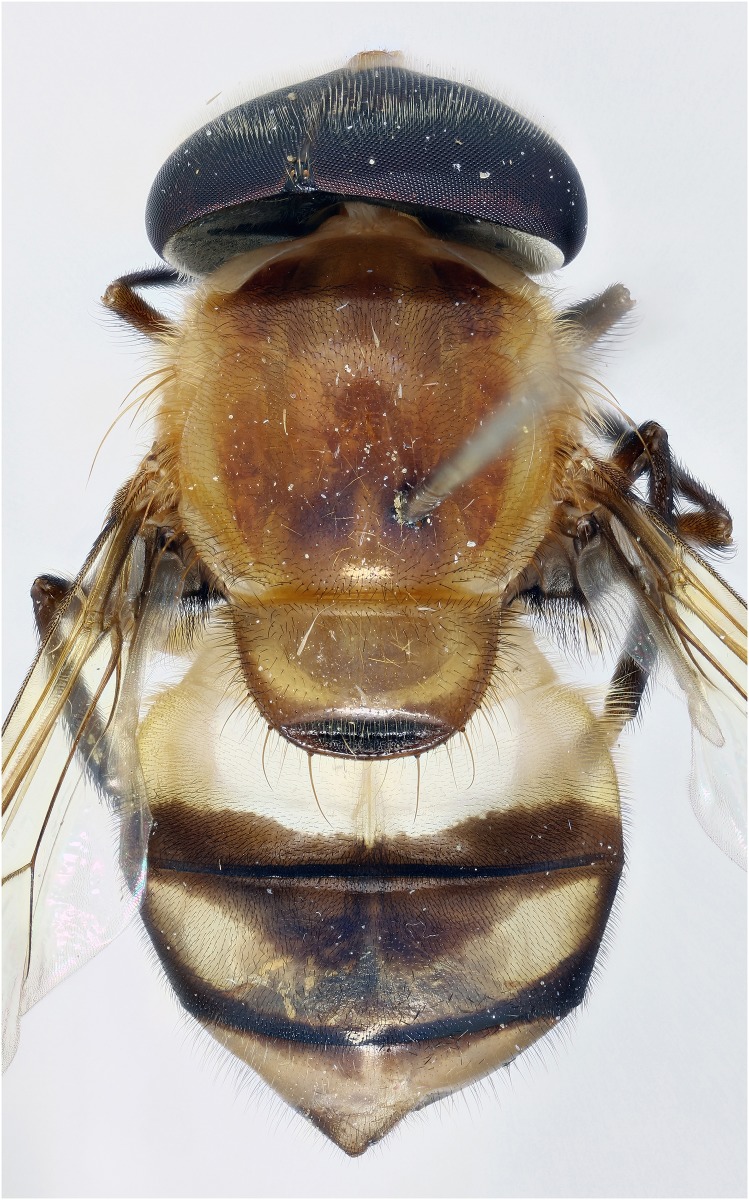
*Copestylum tigrinum* sp. nov. Male holotype, whole body, dorsal view.

Figs 4D, 8, 19A, 19B and 21

*Overall appearance*: a yellow, orange species without clearly defined, dark maculae on the scutum, jet black tibia and sloping eyes in the male; male genitalia with L-shaped surstylus and cercus triangular-shaped and extremely narrow at apex.

*Adult size*: length (mm): body = 9.2−9.8 (n = 2), wing = 8−8.7 (n = 3)

Male holotype

*Head*: face orange below and fading into yellow above the tubercle, without a central vitta; eyes elongate and yellow pilose, pile almost completely absent on lower third; lunule orange (Fig 4D); scape and pedicel with yellow setae.

*Thorax*: scutum orange with vague brownish maculae anteriorly and posteriorly; scutum with pile not much longer at the rear margin; pile black except for a band of yellow pile starting just anterior to the transverse suture and ending before the rear margin; lateral setae orange: 3 above wing insertion and 2 on postalar callus; pleuron dark orange in the lower half, lighter orange above and with orange pile and an orange seta on posterior anepisternum; scutellum orange except for black apex and lateral margins (Fig 8); scutellum with orange pile; marginal setae orange, apical pair thicker and longer than the less conspicuous setae on the lateral margin; coxa and trochanter orange, femur orange except for black apex of metafemora; tibia black, basitarsomere yellow tarsomere 2 partially yellow, tarsomeres 3–5 black; coxa, trochanter and femur with orange pile except postero-apical end of femur and anterior face of metafemora with black pile; tibia and tarsus with black pile; wing hyaline, only slightly sepia antero-apically; pterostigmal spot black, including the apex of cell C; basal half of wing extensively bare; alula wholly microtrichose; halter with pedicel brownish yellow and capitulum white; calypter black.

*Abdomen*: T1 yellow and yellow pilose; T2 yellow except for black posterior margin and yellow pilose except black on posterior margin and apical end of lateral margins; T3&4 black, except each with a pair of comma-shaped, yellow fasciae whose narrow ends meet near the posterior margin and pile all black on T3 and all white on T4 (Fig 21); S1–3 yellow and yellow pilose; S4 black and yellow pilose.

*Genitalia*: in lateral view, cercus triangular-shaped and narrowing to apex; surstylus L-shaped, with basal section about as long as the apical section (Fig 19A); surstylar apodeme conspicuously curved upwards at base and expanded apically with a black, elongate sclerotised apical extension (Fig 19B).

*Female*: unknown.

*Early stages*: pupal spiracles long, longer than distance apart, oval not circular in cross-sectional shape, pile inconspicuous; posterior breathing tube long, transverse ridge clearly above the mid-point of the length of the tube and parallel-sided at apex, not tapering and mostly smooth and shining above the transverse ridge with scattered punctures; prolegs on abdominal segments 1–4, reducing in size and number of crochets from segment 1 to 4.

*Material examined*: *holotype*: 1♂ with genitalia dissected, TRINIDAD AND TOBAGO: Trinidad, Simla, nr (‘near’) Arima, 27.vii–9.viii.08, E.G. Hancock / Hunterian Mus, GLAHM, Entry No. 783 / Not reared [HM]. *Paratypes*: TRINIDAD: 1♂, Trinidad, Lopinot, 24.vii.1999, E.G. Hancock; 1♂, Trinidad Northern Range, Lopinot, ex decaying cocoa pod [*Theobroma cacao* (Malvaceae)] lying on the ground, L: 22.vii.1998, leg. G.E. Rotheray. Paratypes with genitalia dissected [HM & NMS].

*Etymology*: the epithet ‘*tigrinum*’ refers to the similarity between the black and yellow marks of a tiger and the black and yellow fasciae on the abdomen of this species.

*Taxonomic notes*: *C*. *tigrinum* is similar to *C*. *musicanum* in that T3 has a pair of inclined, comma-shaped fasciae, usually meeting medially, but these fasciae sometimes reduced to spots in *C*. *musicanum*. The male genitalia of these two species are also similar in having L-shaped surstyli in lateral view but the cerci are triangular in lateral view, not L-shaped as in *C*. *musicanum*. In both sexes, a difference between these two species is the jet black tibia in *C*. *tigrinum* which in *C*. *musicanum* is dark yellow. Early stages are most similar to *C*. *musicanum* and *C*. *vagum* in having long posterior breathing tubes, longer than larval or puparial width and with the transverse ridge above the middle of the length of the tube. *C*. *tigrinum* appears to be most easily distinguished by the scattered punctures at the apex of the breathing tube in contrast to the more obvious ones of *C*. *vagum* and the lack of punctures in *C*. *musicanum*.

*Biology*: adults were reared from decaying fruits of Malvaceae and this species is only known from Trinidad.

***Copestylum vagum*** (Wiedemann, 1830)

**Fig 22:**
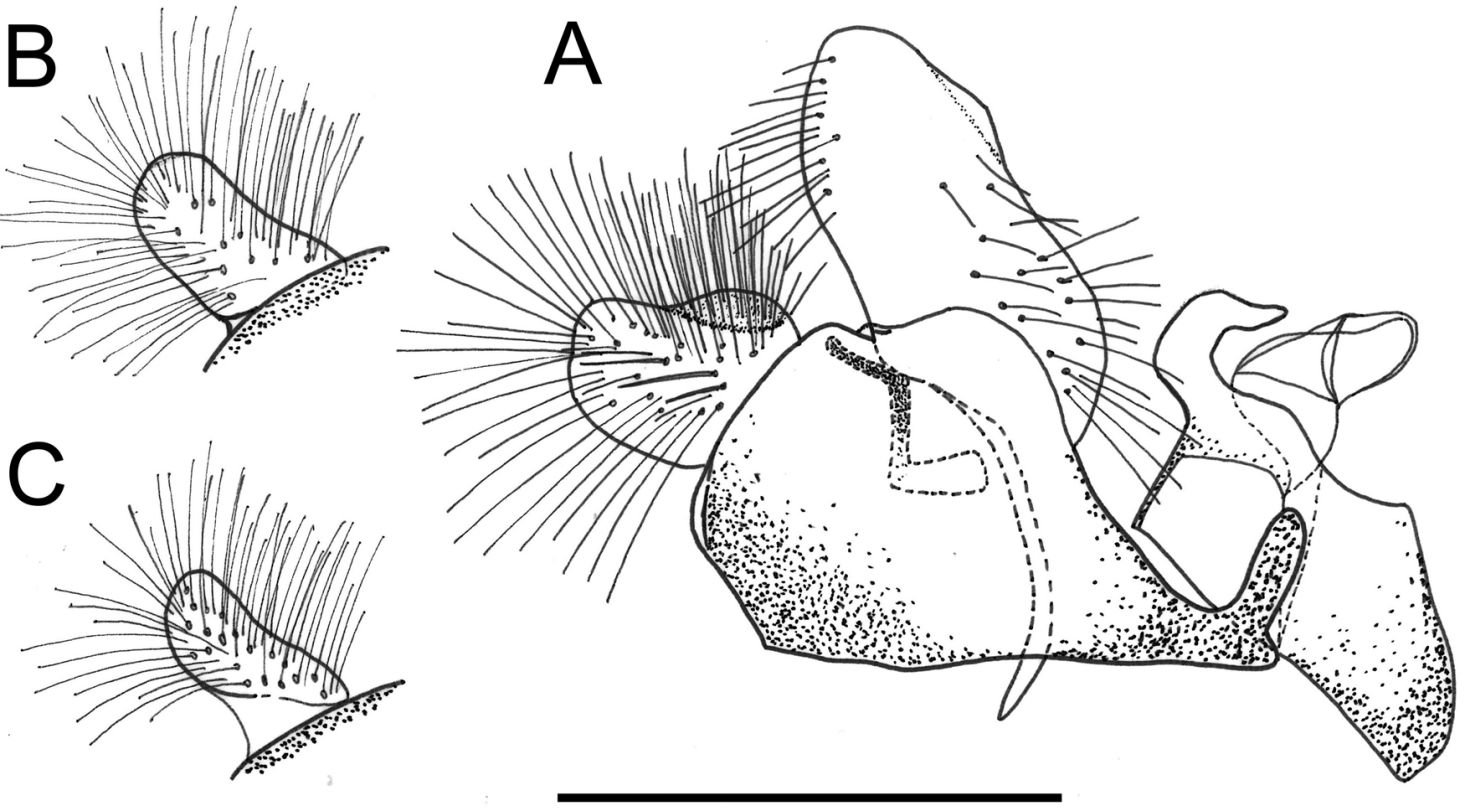
*Copestylum vagum*, genitalia, lateral view, scale line = 0.5mm. A, Male holotype, whole genitalia. B, Specimen from Suriname, cercus. C, Specimen from Costa Rica, cercus.

Figs 3D, 4E, 9 and 22A–22C

*Overall appearance*: a yellow to orange species with T2–4 greenish with narrowly black posterior margins, pleuron with black maculae, face without a central vitta and the top of the male eyes sloping; male genitalia with cercus variable in shape, usually slightly longer than basally wide, apically tapering surstylus and superior lobe with a kinked apex.

*Diagnostic features*: face without a central vitta (Fig 3D); eyes elongate and yellow pilose (Fig 9); lunule yellow; scape and pedicel yellow setulose; scutum orange except for yellow anterior and lateral margins and the middle region from the scutum to the scutellum which is dark and metallic; scutum with black pile anteriorly, yellow pile starting just anterior to the transverse suture and continuing to the rear margin; lateral setae orange: 2 above wing insertion and 2 on postalar callus; lower half of pleuron orange except meron sometimes black-marked, upper half yellow and with yellow pile and an orange seta on the posterior anepisternum (Fig 9); scutellum yellow except for black apical margin; coxa and trochanter yellow, femur orange except for black or blackened apex; tibia and all but tarsomere1 black, tarsomere 1 orange; coxa, trochanter and femur orange pilose, except at apex of femur which is black pilose, otherwise legs black pilose; wing hyaline except for the dark brown pterostigmal spot; basal half of wing extensively bare of microtrichia, alula wholly microtrichose; halter light yellow; calypter dark brown. T1 yellow with yellow pile; T2–4 yellow except for a black apex; T2 with yellow pile anteriorly, black over the black margin and along the side margins; black pilose, T4 white pilose; S1–4 yellowish with darkened posterior margins and entirely yellow pilose; male genitalia with cercus and surstylus in lateral view, slightly longer than wide and round-tipped; superior lobe kinked at apex, not smoothly tapered (Fig 22A).

*Early stages* (Costa Rican material): pupal spiracles long, longer than distance apart, oval not circular in cross-sectional shape, pile inconspicuous; posterior breathing tube long, transverse ridge clearly above the mid-point of the length of the tube and parallel-sided at apex, not tapering and mostly punctured above the transverse ridge, punctures deep and conspicuous; prolegs on abdominal segments 1–4, reducing in size and number of crochets from segment 1 to 4.

*Material examined*: *holotype*: 1♂ in poor condition (prolegs and left mesoleg missing, abdomen partially eaten by *Anthrenus*), with genitalia dissected, *Volucella vaga* Wd (handwritten) / Brasilia, Freireiss. / Typus, D386 (on a red label) [NMW]. *Additional material*: COSTA RICA: 1♂ (genitalia dissected but over-cleared) and 1♀ with puparia, Guanacaste, Rincón de la Vieja, Estación San Gerardo, ex decaying fruit of *Crescentia cujete* (Bignoniaceae), L: 15.viii.2001, A: 19.ix.2001 (♂), A: 17.ix.2001 (♀), leg. G.E. Rotheray; 1♂ (CRI000545863), Est. Queb. (‘Quebrada Station’) Bonita, 50m, Res. Biol. Carara, Prov. Punt., L-N-194500, 469850, ii.1991, leg. M. Moraga; 1♀, Estac. Cacao, 1000–1400m, SW side Volcan Cacao, Guanac. Pr., UTM 323300, 375700, ix.1989, URCG R. Blanco & C. Chavez [INBio & NMS]; SURINAME: 1♂, Brownsberg, 04°56′45″N 55°10′59″W, Malaise trap main camp, 4.iii-1.iv.2006, leg. M. Reemer; 1♀, Bakhuis Mts., 04°46′33.8″N 56°46′21.6″W, 95m. asl, Air strip, Malaise trap, 3–13.iii.2006, leg. B. De Dijn & A. Gangadin [NMS].TRINIDAD: 1♀, Arima, VII.1998, Gl. Univ. Epdtn, reared from larva collected in cocoa fruits [*Theobroma cacao* (Malvaceae)], A: 25.vii.1998 [HM].

*Taxonomic notes*: although Wiedemann [8] described *C*. *vagum* as being green, the holotype is orange and has probably faded with time. This may also explain colour variation of other *C*. *vagum* specimens. Individuals vary in the colour of the scutum and pleuron, as well as in the size of the male cercus (Fig 22A–22C). The scutum can be yellow with a black macula at the rear margin (holotype and specimens from Costa Rica) or bear a black vitta of variable width (specimens from Surinam and Trinidad). Curran [9] refers to such variation in relation to specimens at the Vienna Museum. The pleuron can be yellow (holotype), yellow with black meron (specimens from Surinam and Trinidad) or yellow with black katepisternum, katepimeron and meron. All specimens share the same general shape of the cercus, but in the holotype the cercus is broader than in other males we examined (Fig 22A–22C). Slight differences can also be observed in the shapes of the surstylus and surstylar apodeme. Early stages are similar to those of *C*. *musicanum* and *C*. *tigrinum* in having a long posterior breathing tube, longer than body width and with the transverse ridge above the mid point of its length. *C*. *vagum* differs from these two species in having the apex of the posterior breathing tube with conspicuous punctures.

*Biology*: adults were reared most frequently from Costa Rica and Trinidad from decaying fruits of species of Bignoniaceae and Malvaceae.

***Copestylum willistoni*** Ricarte & Hancock **sp. nov**. urn:lsid:zoobank.org:act: 16A0FD99-4E0E-42E4-9DD6-EF91FE134FBD

Figs 4F, 10 and 23

**Fig 23:**
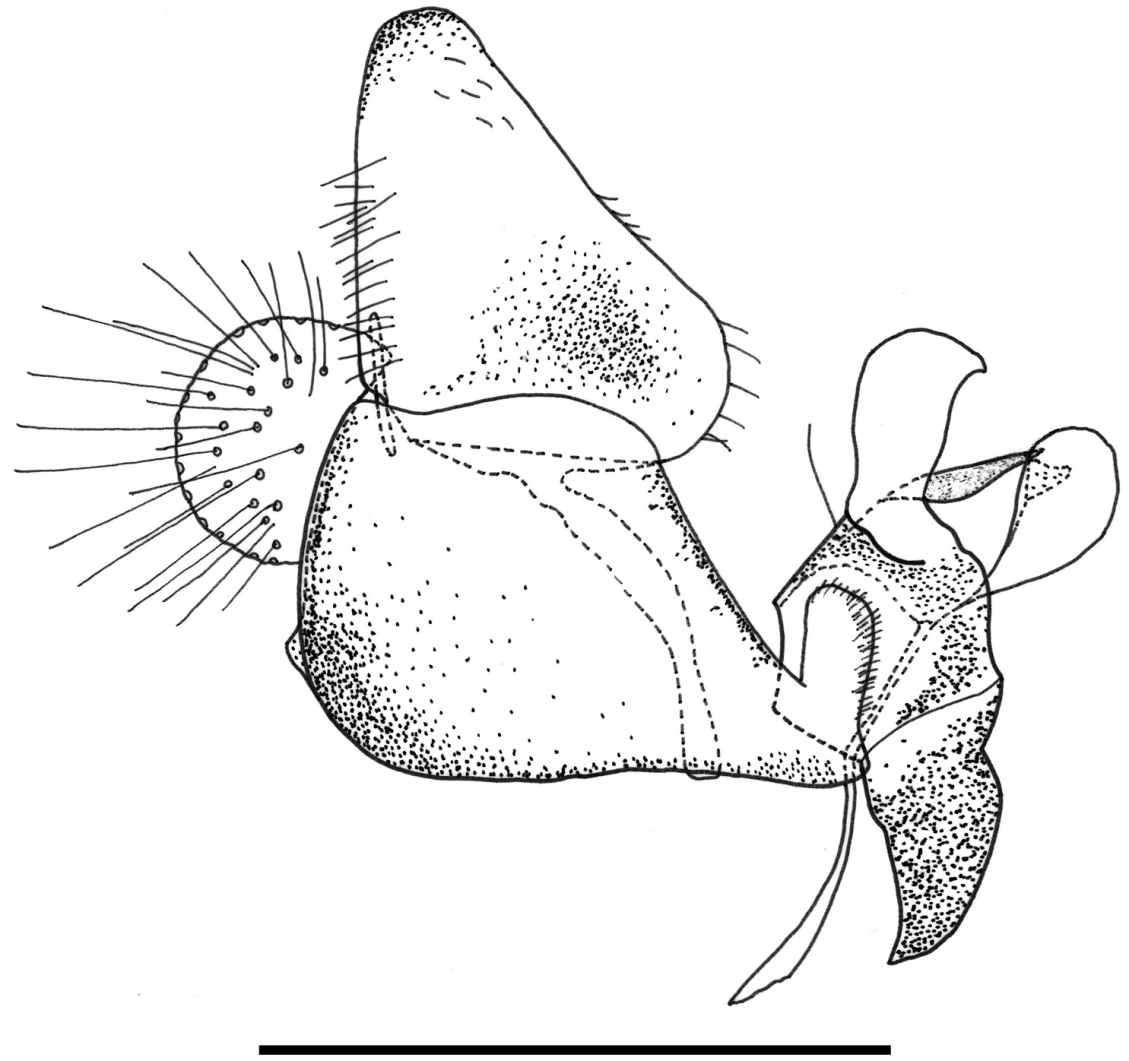
*Copestylum willistoni* sp. nov. Male holotype, genitalia, lateral view. Scale line = 0.5mm.

*Overall appearance*: a dark species with a faint central vitta and slight central tubercle on the face, thoracic setae black and T3&4 yellow marked; pleuron yellow-orange with a unique W-shaped arrangement of black maculae; male genitalia with a unique, bar-shaped superior lobe with only a slight apical hook.

*Adult*: length (mm): body = 10−11, wing = 8.7−9.7 (n = 3); one female exceptionally small, body length = 7.7mm, wing length = 7.3mm.

Male holotype

*Head*: face with a dark, central vitta; in lateral view, eyes rounded and orange pilose, except pile short and scattered on the lower quarter of the eye (Fig 4F); lunule orange; scape and pedicel black and yellow setulose.

*Thorax*: scutum metallic dark with opalescent reflections (more evident under artificial light), except for a yellow lateral margin from the postpronotum to the postalar callus and two medial vittae ending before the transverse suture; scutum with black and yellow pile of different lengths intermixed and pile conspicuously longer at the rear margin; lateral setae black: 3 above wing insertion and 3 on the postalar callus; pleuron yellow except for a black katepisterum, anterior anepisternum and anepimeron and a dark marked meron forming a W-shape (Fig 10); posterior anepisternum with a black seta postero-dorsally; pleuron yellow pilose; scutellum with a slight, pre-apical, yellow depression; scutellum yellow with a broad, black, metallic fascia across the middle; except for pale pile at base, scutellum with black pile of variable size, longest pile less than half length of the apical setae and conspicuous black setae round the margin; legs dark brown to black and extensively black pilose; legs dark yellowish and extensively black pilose, except for some yellow pile on pro- and metatrochanters; wing lightly sepia in cells C, SC and stigma; cells BM and CuP extensively bare, cells R, CuA1 and anal lobe with bare areas of different sizes, alula wholly microtrichose; halter with pedicel yellow and capitulum white; calypter dark brown to black.

*Abdomen*: T1 yellow and yellow pilose; T2 orange in anterior half with orange fasciae extending to the posterior corners, otherwise black, pile orange and black accordingly except lateral margins black pilose; T3&4 black with yellow anterior margin; anterior margin of T3&4 mostly black pilose and orange pile at the middle of the anterior margin; S1&2 yellow and yellow pilose; S3&4 extensively black and mostly yellow pilose.

*Genitalia*: in lateral view, cercus slightly shorter than basally long, surstylus triangular with an almost straight anterior margin, longer than the other two margins; superior lobe uniquely bar-shaped, with a rounded apex and only a slight hook (Fig 23).

*Female*: same as the male, except for the following characters: vertex black and black haired; frons yellow with a medial, black stripe from the vertex to the lunule; frons extensively black haired; eye facets of about the same size; eye hairs shorter than those in male; scutum extensively yellow haired; pro- and mesofemora with a black macula basally; band of black hairs on terga 2&3 more reduced than that in male.

*Early stages*: pupal spiracles long, longer than distance apart and black marked; pile of pupal spiracles inconspicuous; posterior breathing tube with transverse ridge just above the middle of the length of the tube, squared-off, not tapered at apex, heavily punctured above the transverse ridge; prolegs with crochets on abdominal segments 1–6, reducing in size and number of crochets towards segment 6; lappets and projections on segments 6 and 7 not disguised by a dense coating of vestiture.

*Material examined*: *holotype*: 1♂ with puparium, COSTA RICA, Alajuela, Guatuso, Buenavista, Fca. La Garroba, 93m, N10 48 11.7 W 84 53 23.2, ex Sapotaceae fruit, L: 13.vii.2010, P: 28.viii.2010, A: 10.ix.2010, various collectors [INBio]. *Paratypes*: COSTA RICA: 6♂ with puparia (all puparia except one are contained in the same plastic capsule), Cartago, Turrialba, Tres Equis, P.N. Barbilla, ex cocoa fruits [*Theobroma cacao* (Malvaceae)], L: 21.xi.2000, A: no data, leg. E. Rojas; 1♂ with puparium, Alajuela, Guatuso, Buenavista, Fca. La Garroba, 93m, N10 48 11.7 W 84 53 23.2, ex Sapotaceace fruit, L: 13.vii.2010, P: 28.viii.2010, A: 8.ix.2010, various collectors; 1♀ with puparium, Alajuela, Guatuso, Buenavista, Fca. La Garroba, 93m, N10 48 11.7 W 84 53 23.2, ex Sapotaceace fruit, L: 13.vii.2010, P: 30.viii.2010, A: 13.ix.2010, various collectors; 1♀ with puparium, Alajuela, Guatuso, Buenavista, Fca. La Garroba, 93m, N10 48 11.7 W 84 53 23.2, ex Sapotaceace fruit, L: 13.vii.2010, P: 21.viii.2010, A: 3.ix.2010, various collectors [INBio & NMS]. TRINIDAD: 1♂, Hollis Dam, L: vii.1998, A: 16.viii.1998, ex tree sap, Glasgow. Univ. Epdtn., specimen in poor condition: three legs and one wing missing, base of the abdomen destroyed ventrally and genitalia dissected but over-cleared [HM].

*Etymology*: The epithet ‘*willistoni*’ refers to the American dipterologist Samuel Wendell Williston, who described many *Copestylum* and other Diptera species during his working life.

*Taxonomic notes*: the single specimen from Trinidad differs from Costa Rican specimens in the following characters: scutum mostly yellow and with a few black pile only on the posterior margin and postalar callus, otherwise yellow pilose; anepimeron extensively yellow; scutellum yellow, only centrally dark brown. The Trinidadian specimen appears to be only a pale specimen of *C*. *willistoni*. *C*. *willistoni* is similar to *C*. *araceorum* and *C*. *cyclops* in having a central vitta on the face, although it is relatively faint in *C*. *willistoni* and *C*. *cyclops*. *C*. *willistoni* shares with *C*. *araceorum* black thoracic setae and mid femora entirely dark pilose. In *C*. *cyclops*, the thoracic setae are orange and the mid femora pale pilose basally. Apart from a faint central vitta, *C*. *willistoni* differs from *C*. *araceorum* by the less pronounced central tubercle on the face, T3 mostly black pilose, only a few pale pile at the anterior margin and the different shapes of the surstylus and superior lobe. Early stages are most similar to *C*. *araceorum* in having prolegs with crochets on the first 6 abdominal segments. In *C*. *cyclops* and other Vagum group species, prolegs with crochets are absent on segment 6. Apart from pupal spiracles longer than their distance apart, *C*. *willistoni* is readily distinguished from *C*. *araceorum* by the apex of the breathing tube which is squared-off apically and does not taper and is punctate.

*Biology*: adults were reared from decaying fruits of species of Malvaceae and Sapotaceae in Costa Rica and from exuding tree sap in Trinidad.

### Group and species systematics of *Copestylum* reared from live and dead flowers

#### Cinctiventre species group

*Diagnosis–overall appearance*: adult shiny, dark, metallic; sides of face with yellow vittae and usually, tomentum; scutum with distinct layers of short pile orientated anteriorly and long pile orientated posteriorly; pile noticeably longer at the rear margin; scutellum with an apical depression; postero-apical margin of the mid-femora usually with a group of conspicuously long, thick setae; abdominal tergites more or less uniform in background colour, without a contrasting pale colour on T1&2. Early stages with prolegs and crochets on segments 1–3; posterior breathing tube above the transverse ridge either flattened dorso-ventrally or becoming wider than below the ridge; if tube not like this then tube heavily punctured.

#### Adult

**Fig 24:**
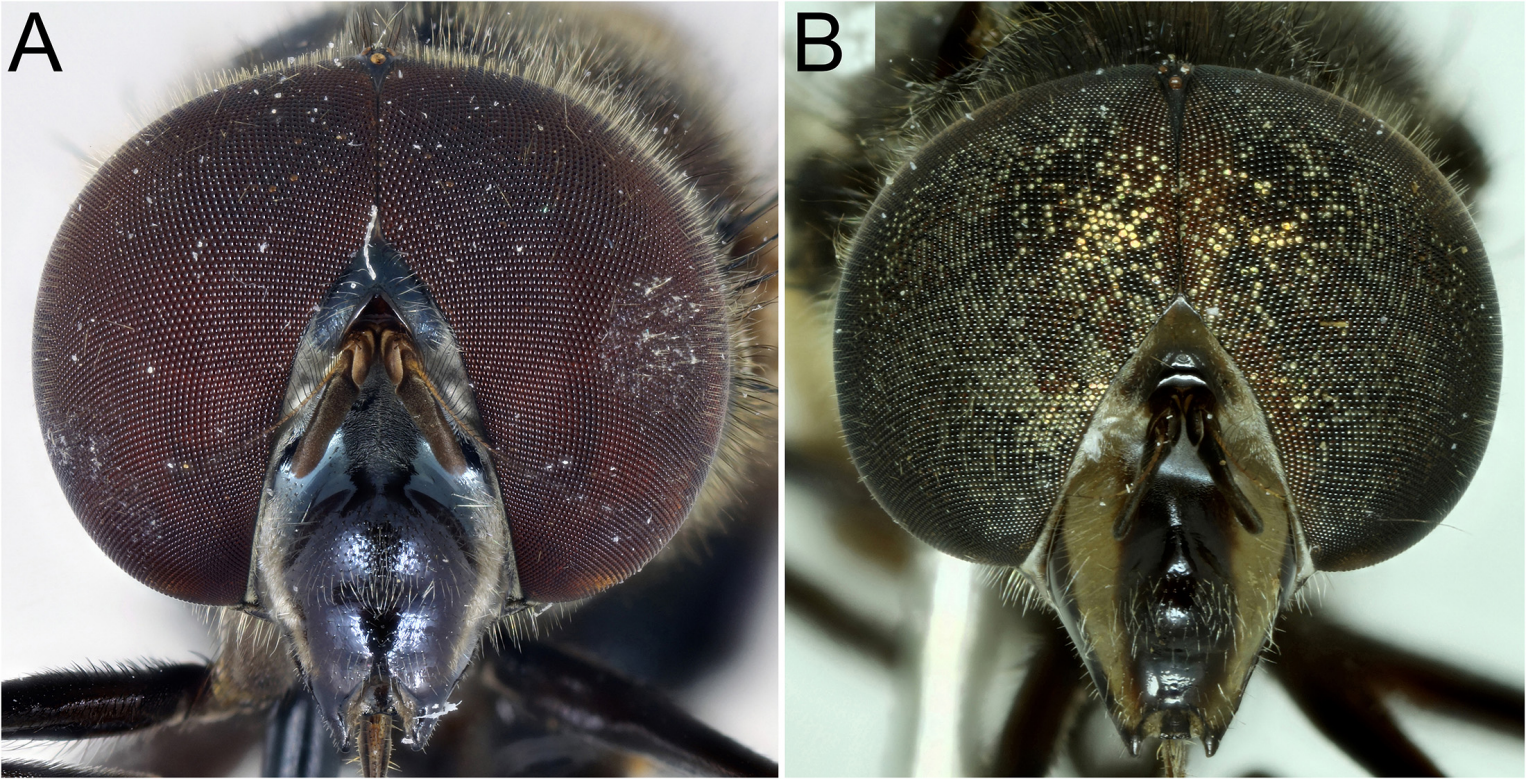
*Copestylum* species of the Cinctiventre group, head, anterior view. A, *Copestylum azofeifa* sp. nov., male holotype. B, *Copestylum cinctiventre*, specimen from Trinidad, male.

**Fig 25:**
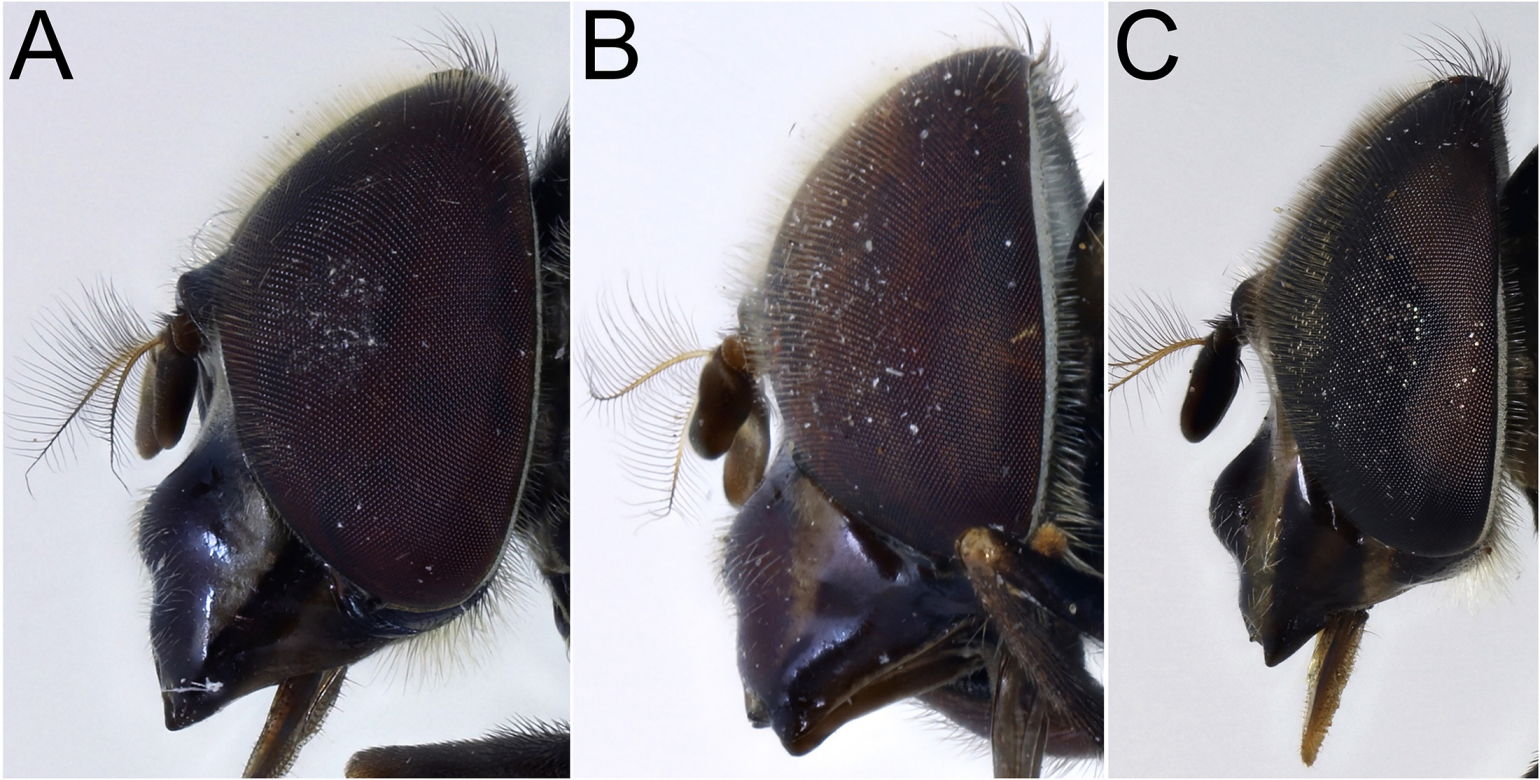
*Copestylum* species of the Cinctiventre group, head, lateral view. A, *Copestylum azofeifa* sp. nov., male holotype. B, *Copestylum cinctiventre*, specimen from Trinidad, male. C, *Copestylum ellenae* sp. nov., male holotype.

*Head*: face pale pilose with background colour metallic dark brown, black or blue-black and central vitta absent (Fig 24 and 24B); usually, yellow to orange vittae on the side of the face and sometimes gena pale-marked (Fig 25A–25C); dust confined to extreme eye margins up to the anterior sides of the frontal triangle or ending just below and extending under the antennae and usually, one or two, separate, tomentose maculae on or next to vittae on the sides of the face, otherwise face shiny (Fig 1); facial tubercle present and clearly defined; face not much extended or drawn out; eyes neither particularly rounded or elongate; eye facets in males only slightly larger dorsally and eyes yellow to orange pilose to about two thirds down the length of the eyes, pile becoming shorter and absent on the lower and rear margins; arista basal half to two thirds orange, the rest black; basoflagellomere dirty yellow to brown, kidney to lozenge shaped varying in length from 1.5–2.5× as long as height with an indistinct pit on the inner, basal margin.

**Fig 26:**
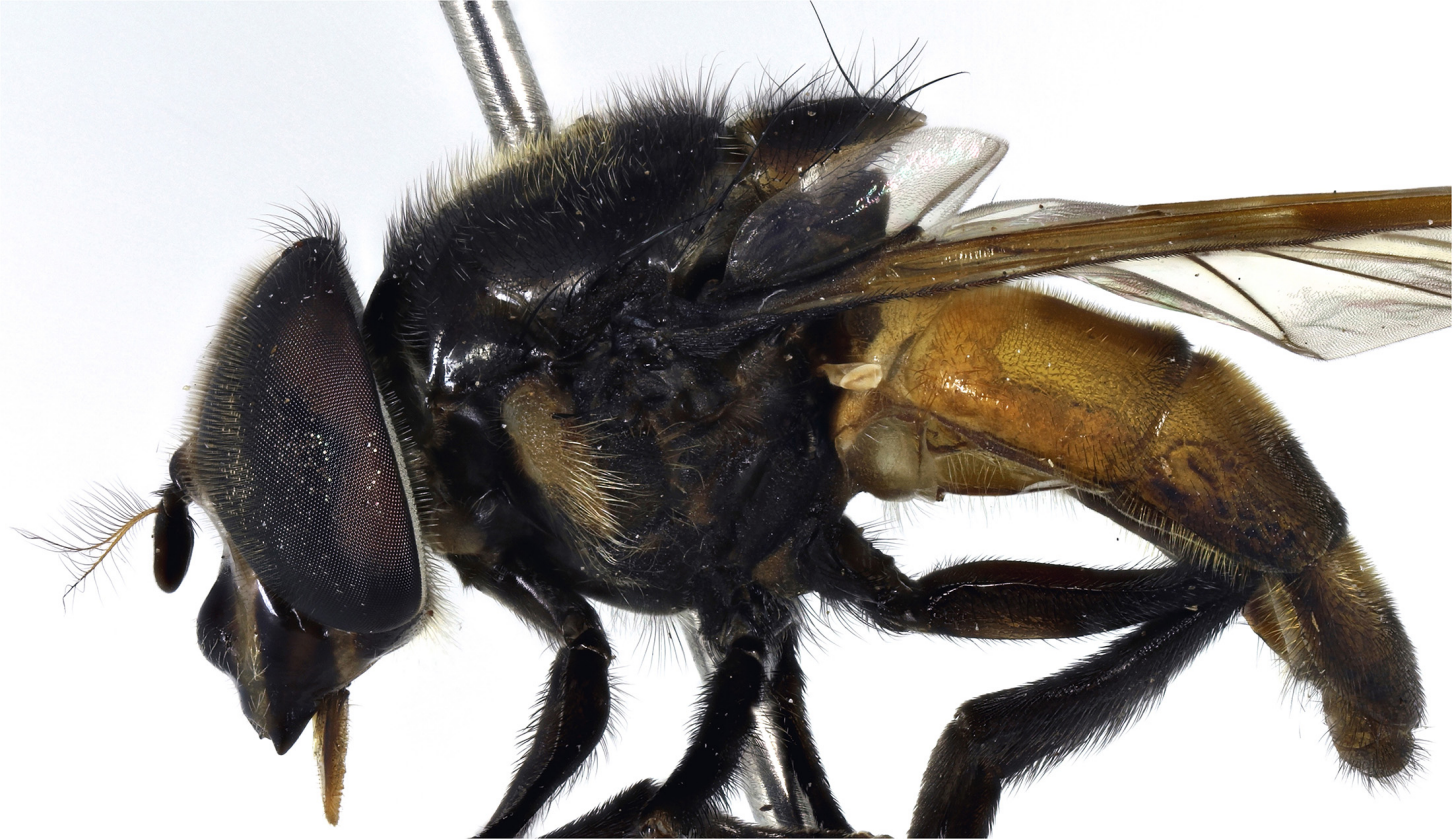
*Copestylum azofeifa* sp. nov. Male holotype, whole body, lateral view, head to the left.

**Fig 27:**
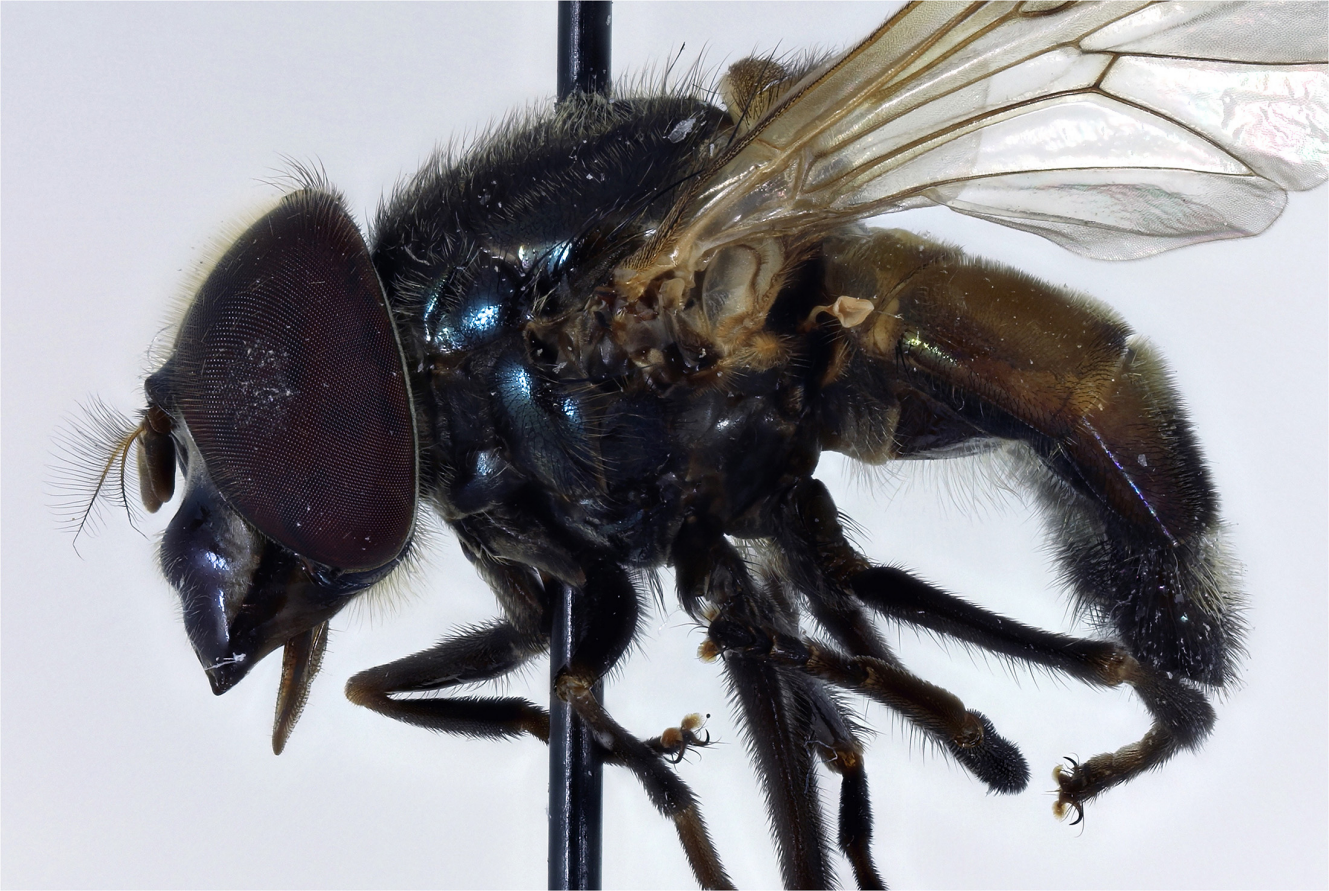
*Copestylum cinctiventre*. Specimen from Trinidad, male, whole body, lateral view, head to the left.

**Fig 28:**
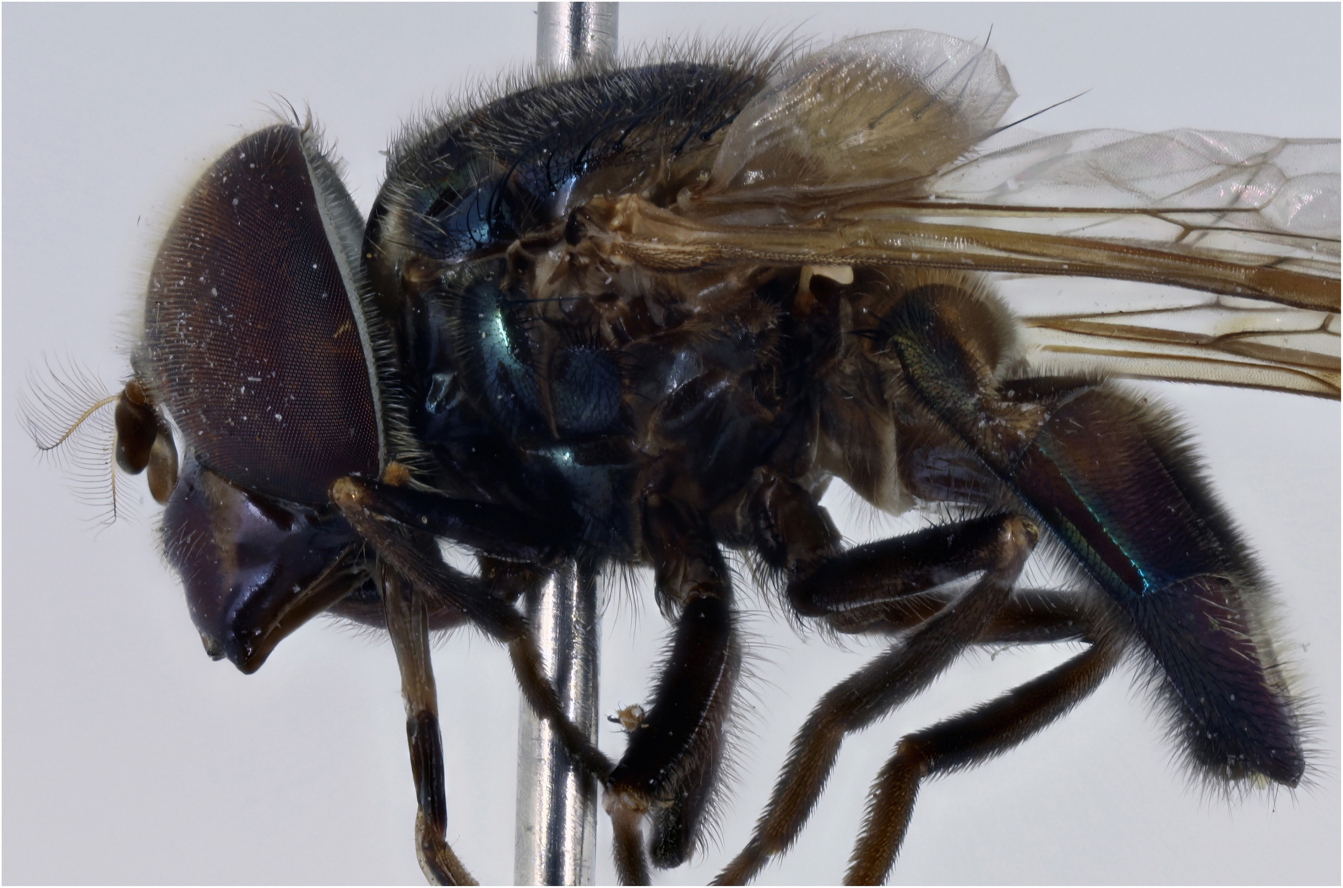
*Copestylum ellenae* sp. nov. Male holotype, whole body, lateral view, head to the left.

*Thorax*: blue-black metallic in colour and lightly dusted (Figs 26–28); anepisternum rarely and katepimeron often yellow marked; scutum coated in a complex pattern of pile, short pile directed forwards and longer pile directed backwards or curved over apically, in males, rear margin usually with pile about 2× as long as elsewhere; long pile before the transverse suture mostly black, elsewhere pale or scattered black pile present, overall effect is a shifting pattern of silvery and dark pilose stripes and blotches according to the angle of view; pile longer in males than females; apart from longer pile, long setae absent at the rear margin; scutellum usually with a shallow, apical depression bearing a rugose surface; wing cells BM and CuP extensively bare of microtrichia; cells DM, CuA1 and anal lobe with bare areas; stigma, cell R1 near stigma and cell C apically lightly brown pigmented; halter white; calypter brown, with marginal hair fringe dark brown; legs dark orange to black, tarsi often paler; apex of mid femora with a few noticeably, long, thick, setae.

**Fig 29:**
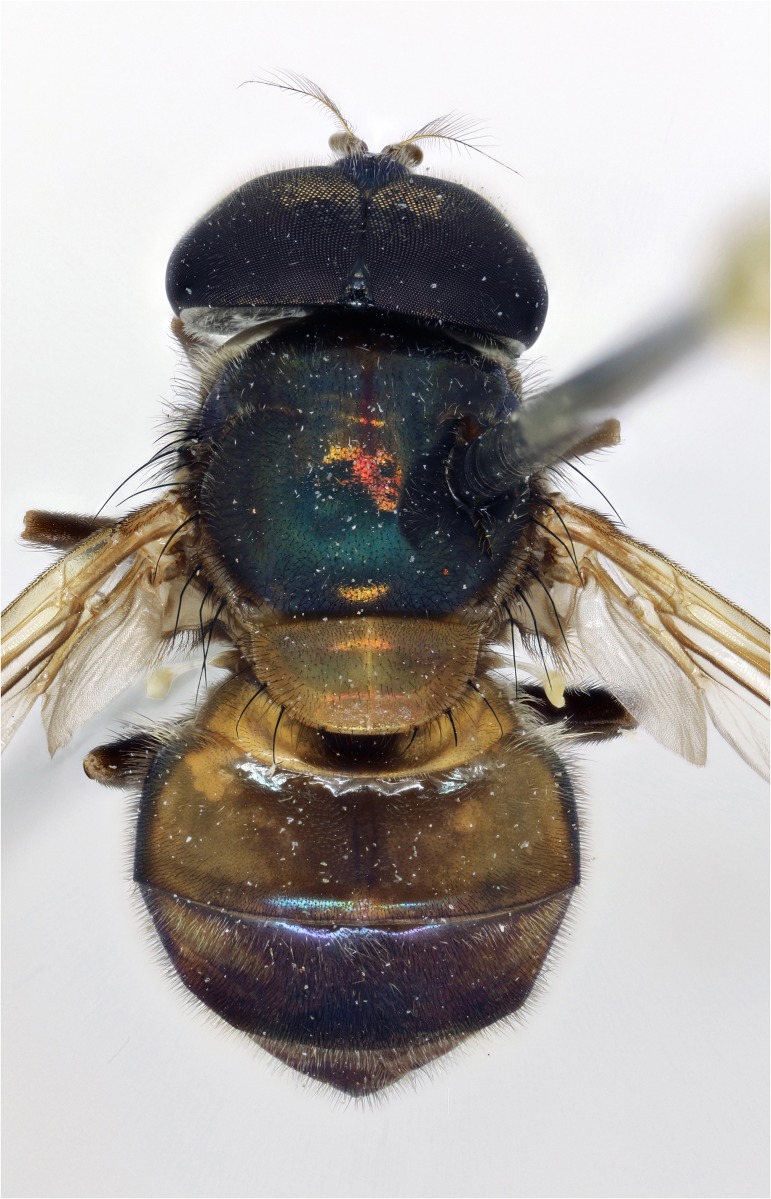
*Copestylum ellenae* sp. nov. Male holotype, whole body, dorsal view.

*Abdomen*: widest at or near the apex of T2 and at this point, abdomen about a third again as wide as the thorax; rest of abdomen tapering to the apex of T5; abdomen pale to dark orange or metallic black; T1 and anterior half of T2 not contrastingly pale marked (Fig 29); tergites pilose, pile upright and becoming longer towards lateral margins and not or only slightly longer on T4; pile variably coloured with white, yellow, orange or black, arranged in fasciae or oval maculae.

**Fig 30:**
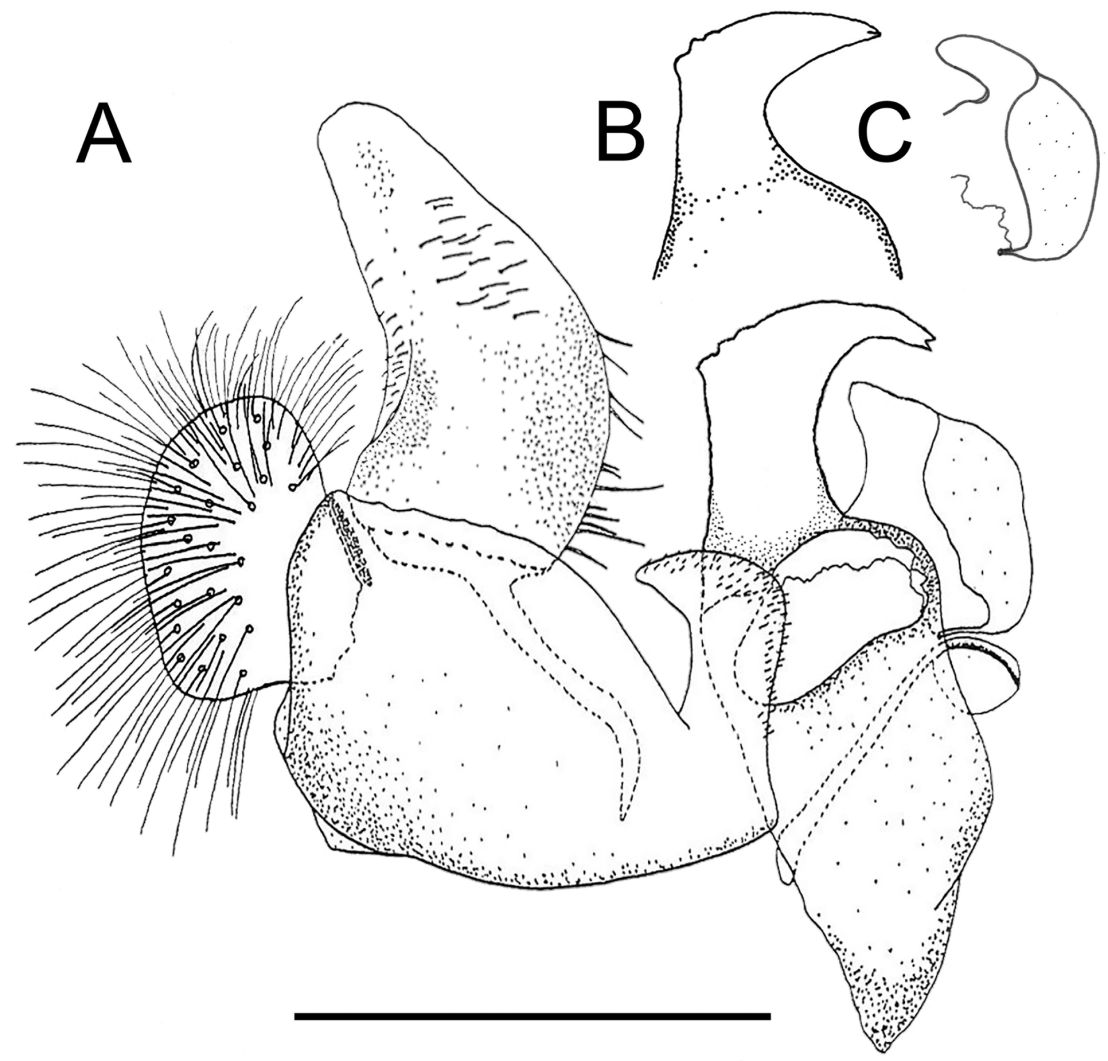
*Copestylum* species of the Cinctiventre group, male genitalia, lateral view, scale line = 0.5mm. A, *Copestylum ellenae* sp. nov., holotype, whole genitalia. B, *Copestylum cinctiventre*, specimen from Trinidad, superior lobe. C, *Copestylum cinctiventre*, specimen from Trinidad, aedeagal hood.

**Fig 31:**
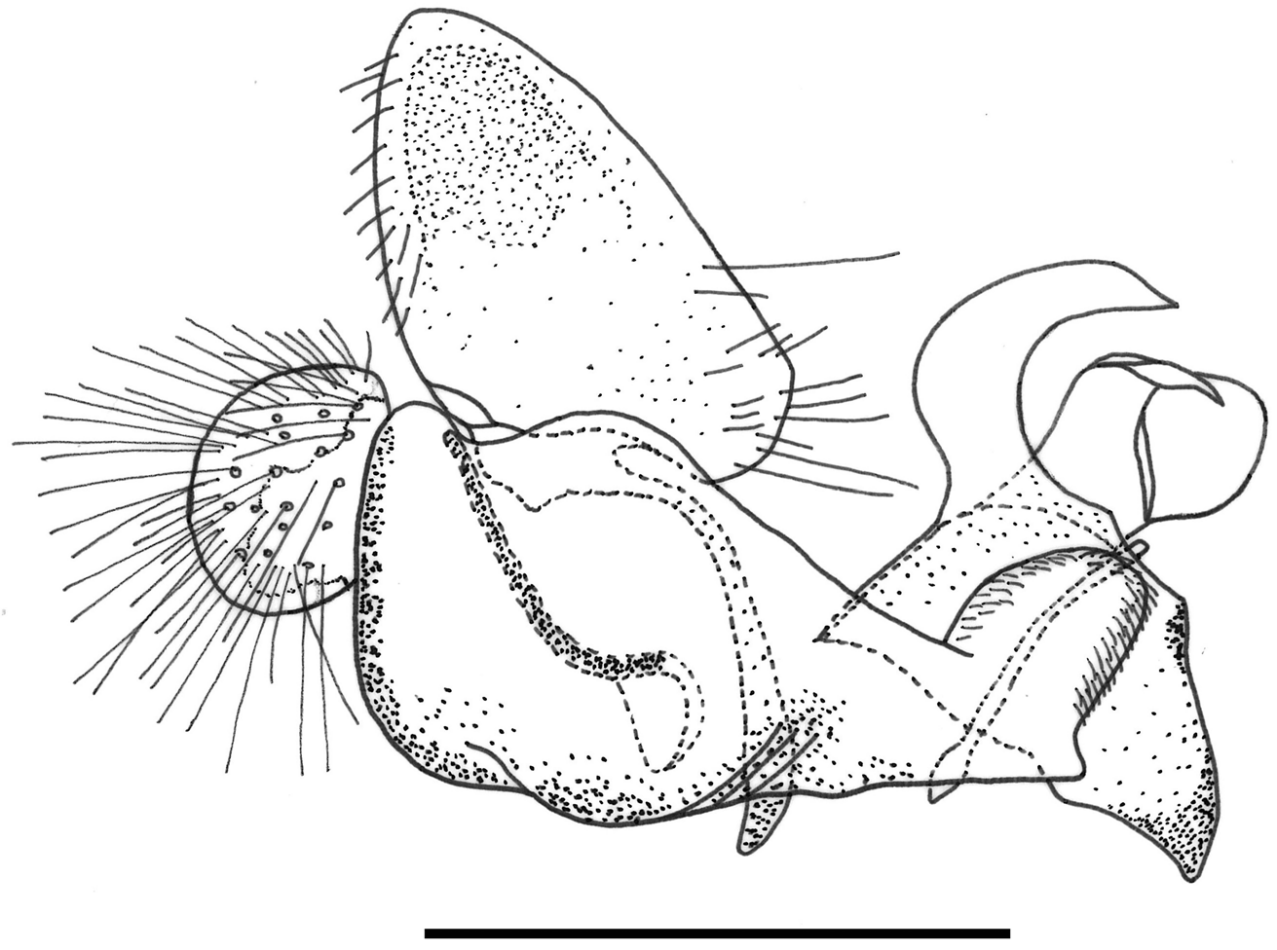
*Copestylum azofeifa* sp. nov. Male holotype, genitalia, lateral view. Scale line = 0.5mm.

**Fig 32:**
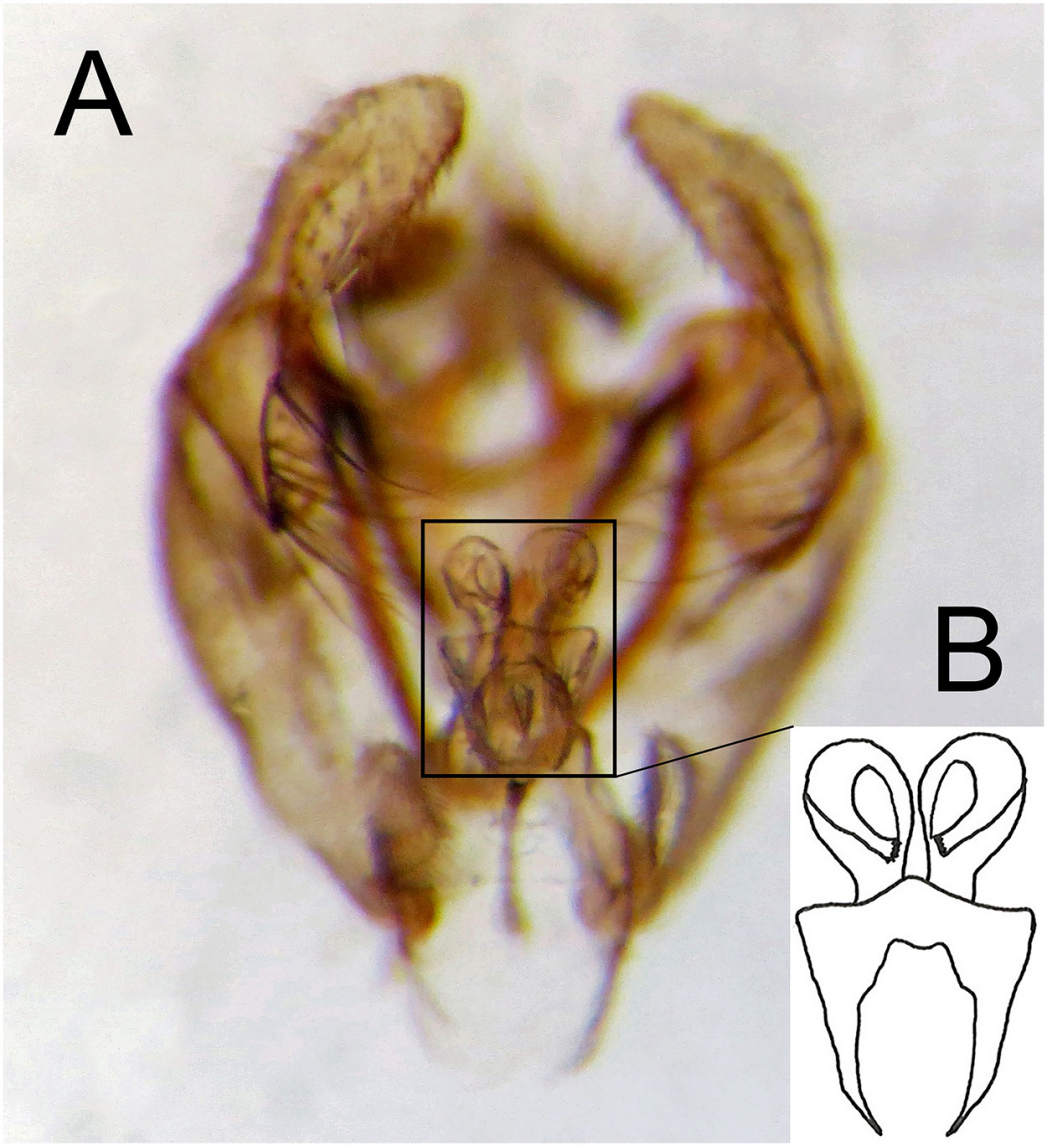
*Copestylum azofeifa* sp. nov., male holotype, genitalia. A, Whole genitalia, ventral view. B, Superior lobe and aedeagal hood, apical view.

**Fig 33:**
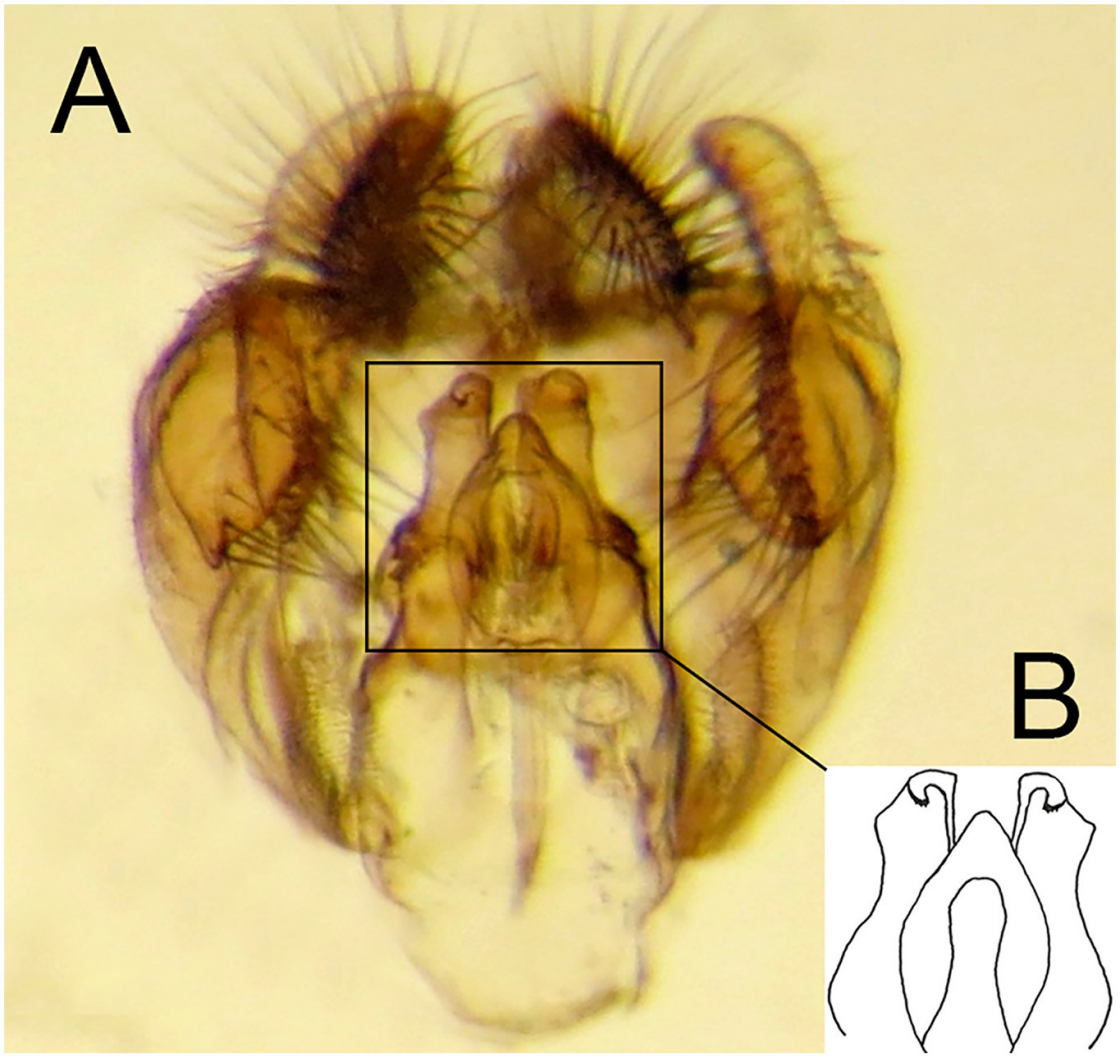
*Copestylum cinctiventre*, specimen from Trinidad, male genitalia. A, Whole genitalia, ventral view. B, Superior lobe and aedeagal hood, apical view.

**Fig 34:**
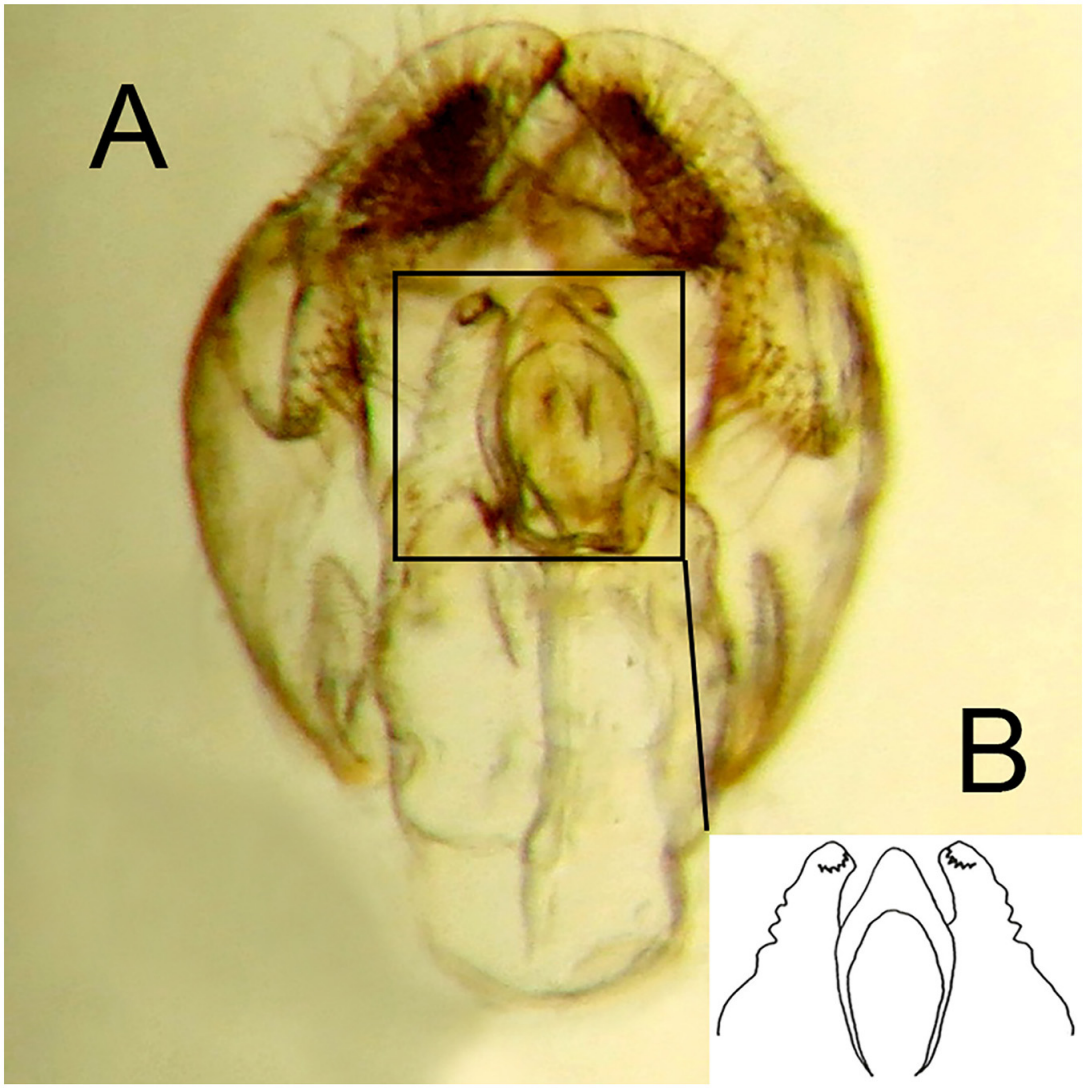
*Copestylum ellenae* sp. nov., male holotype, genitalia. A, Whole genitalia, ventral view. B, Superior lobe and aedeagal hood, apical view.

*Male genitalia*: epandrium with a large, projection at the point of articulation with the hypandrium, usually with the apico-lateral margin tapered and pointing towards the cercus; epandrium may or may not have dark sclerotised lines on margin articulating with the cercus; surstylus variable in shape, usually kinked or curved back above the base and tapering to the apex (Fig 30A) or triangular (Fig 31); hypandrium variable in shape, usually 2× as long than wide across the middle (Fig 30A) or not as long as wide (Fig 31); superior lobe variable in shape, usually with, in lateral view, a pair of conspicuous, broad, hook-like apical processes of variable shape (Figs 31, 30A and 30B) and bearing serrated apices and projections on the outer margin (Figs 32B, 33B and 34B); aedeagal hood variable in shape, usually projecting dorsally, between the hooks of the superior lobe (Fig 30A and 30C) or with side flanges (Figs 31, 32A and 32B).

**Fig 35:**
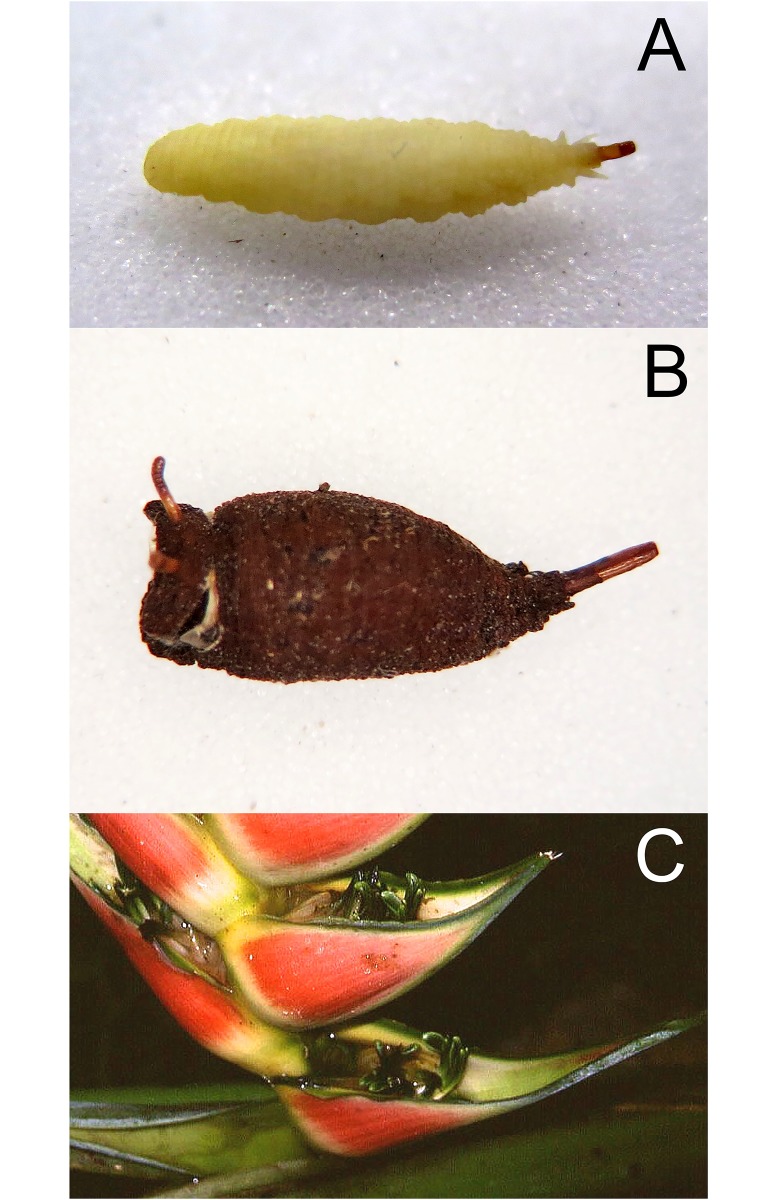
*Copestylum cinctiventre*. A, Specimen from Trinidad, whole third stage larva, head to the left, dorso-lateral view. B, Specimen from Trinidad, whole puparium, lateral view. C, Development site, decaying *Heliconia bihai* flowers (Heliconaceae), Trinidad.

**Fig 36:**
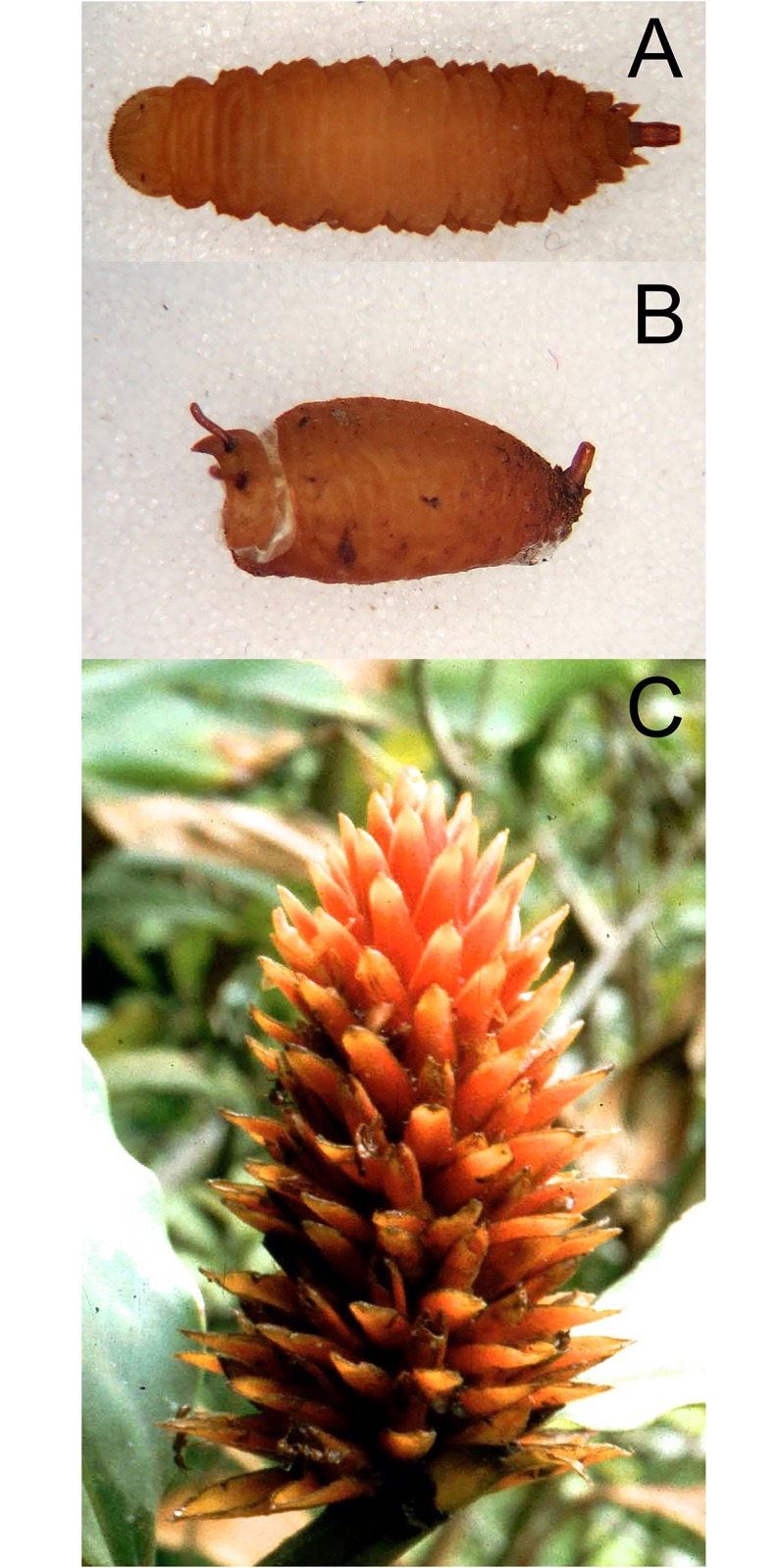
*Copestylum ellenae*, sp. nov. A, Specimen from holotype locality, whole third stage larva, head to the left, dorsal view. B, Holotype whole puparium, lateral view. C, Development site, *Renealmia* flowers (Zingiberaceae), from holotype locality, Costa, Rica.

**Fig 37:**
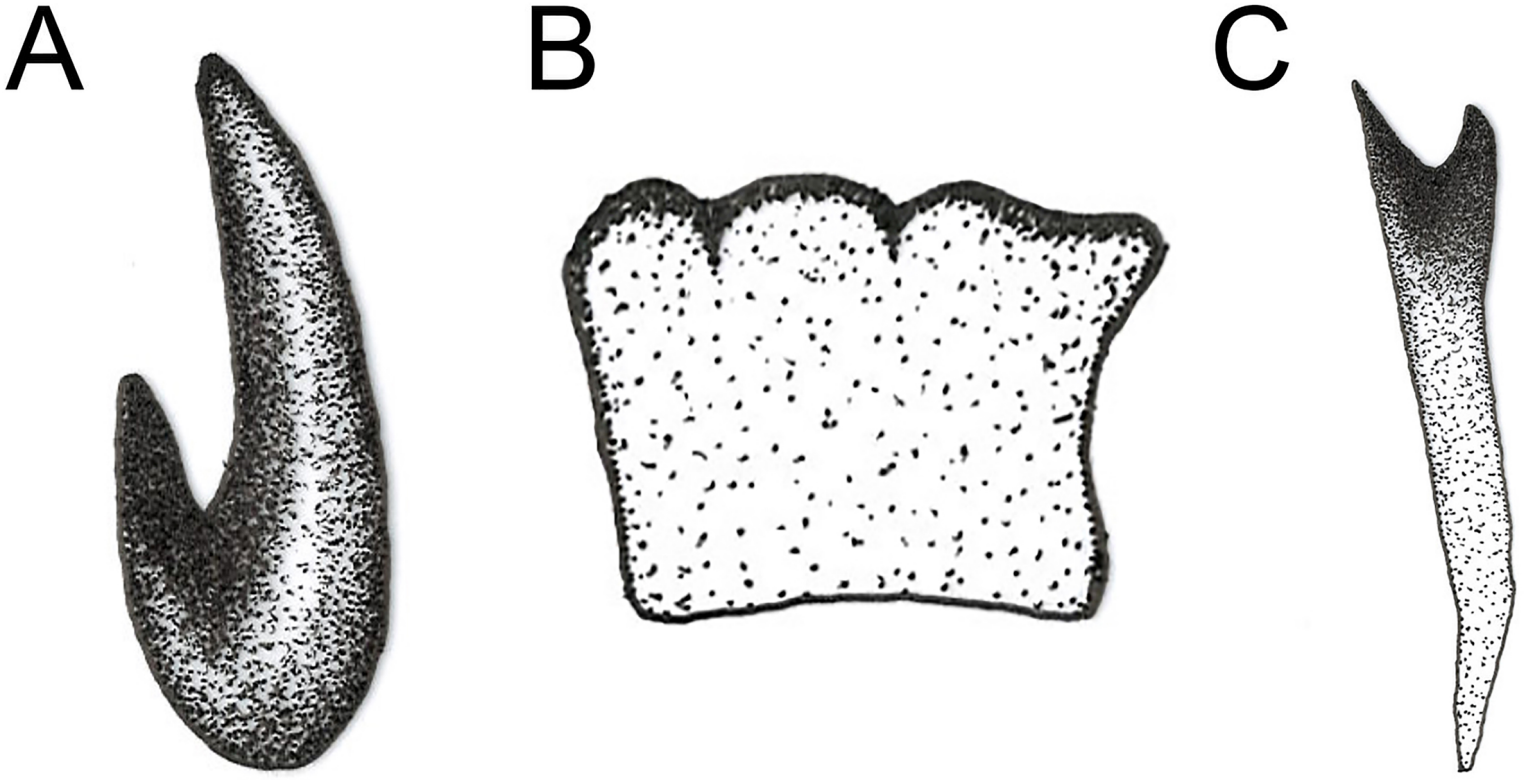
*Copestylum* species of the Cinctiventre group, third stage larva, spicule from anterior fold of prothorax. A, *Copestylum azofeifa*, sp. nov., specimen from holotype locality, spicule in dorso-lateral view. B, *Copestylum cinctiventre*, specimen from Trinidad, spicule in anterior view. C, *Copestylum ellenae*, sp. nov., specimen from holotype locality, spicule in dorso-lateral view.

**Fig 38:**
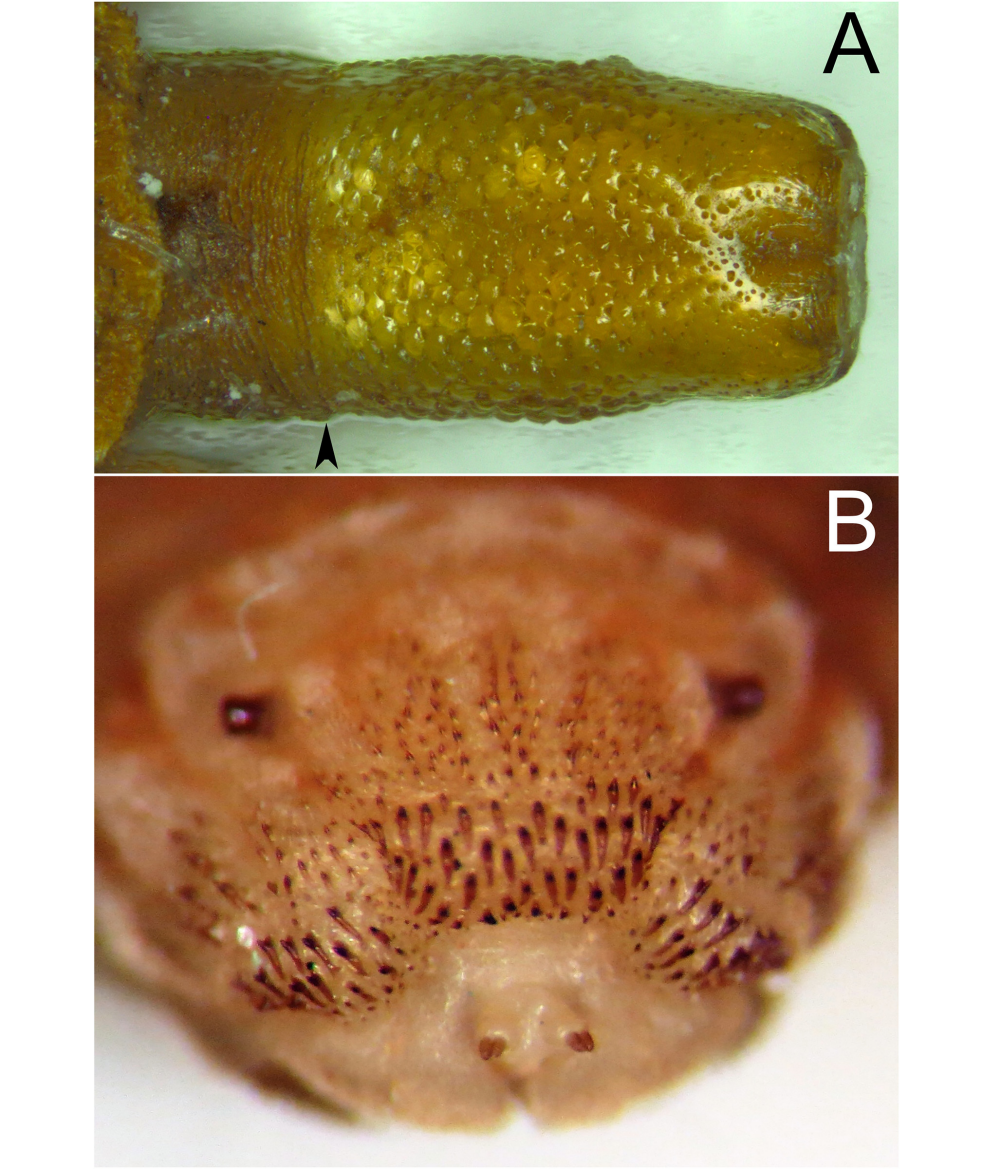
*Copestylum ellenae*, sp. nov. A, Holotype puparium, posterior breathing tube, dorsal view (an arrowhead indicates position of the transverse ridge). B, Specimen from holotype locality, third stage larva, anterior fold of prothorax, anterior view.

**Fig 39:**
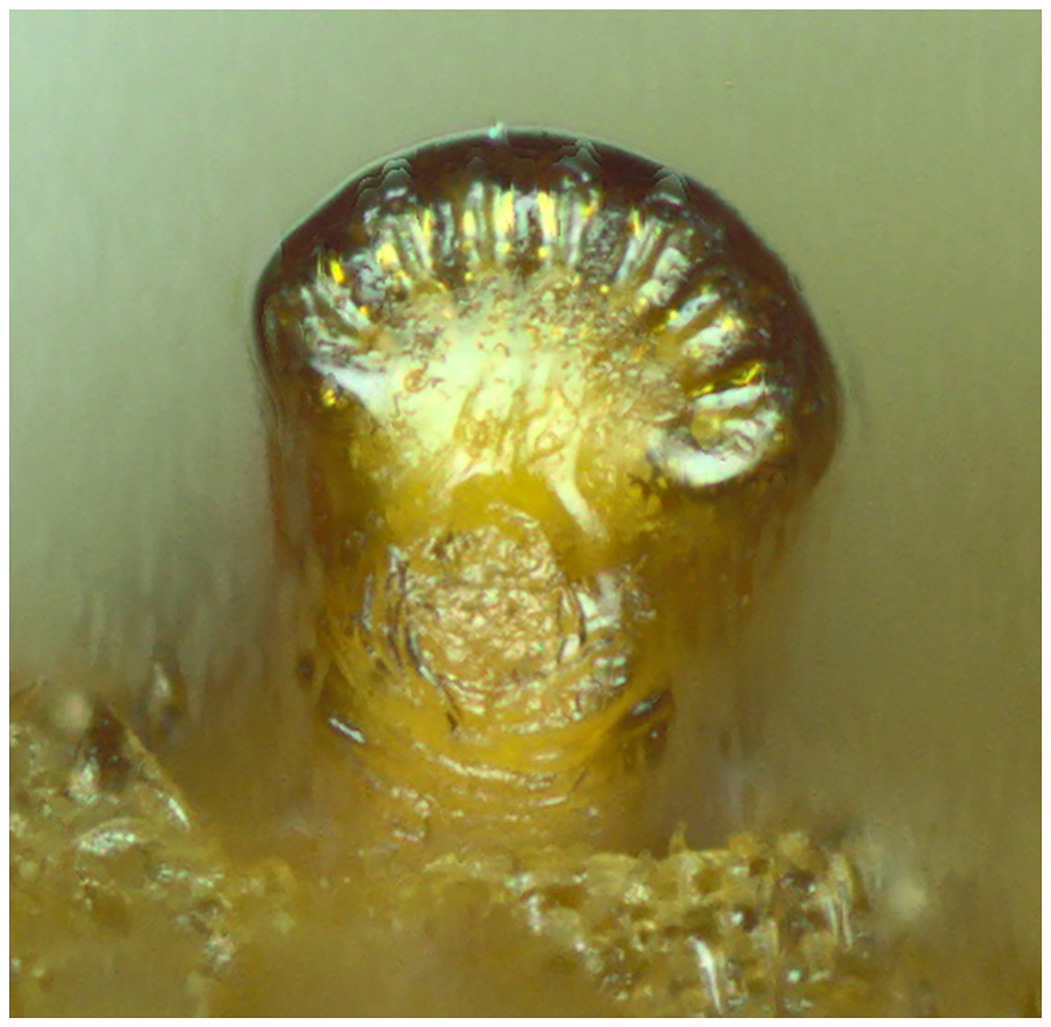
*Copestylum ellenae*, sp. nov. Holotype puparium, anterior breathing tube, lateral view.

**Fig 40:**
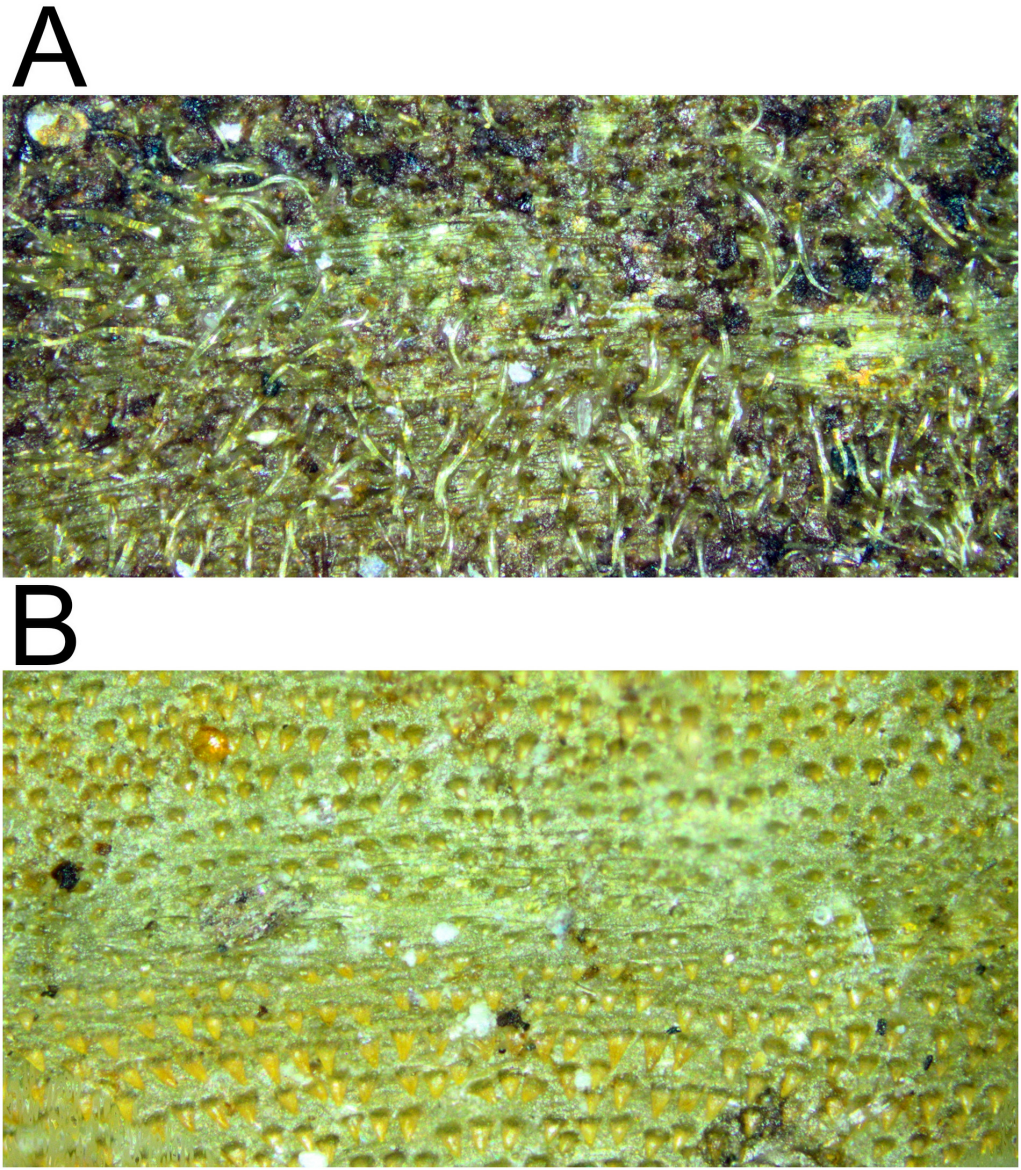
*Copestylum* species of the Cinctiventre group, holotype puparium, 2^nd^ abdominal segment, vestiture, dorsal view. A, *Copestylum azofeifa*, sp. nov. B, *Copestylum ellenae*, sp. nov.

**Fig 41:**
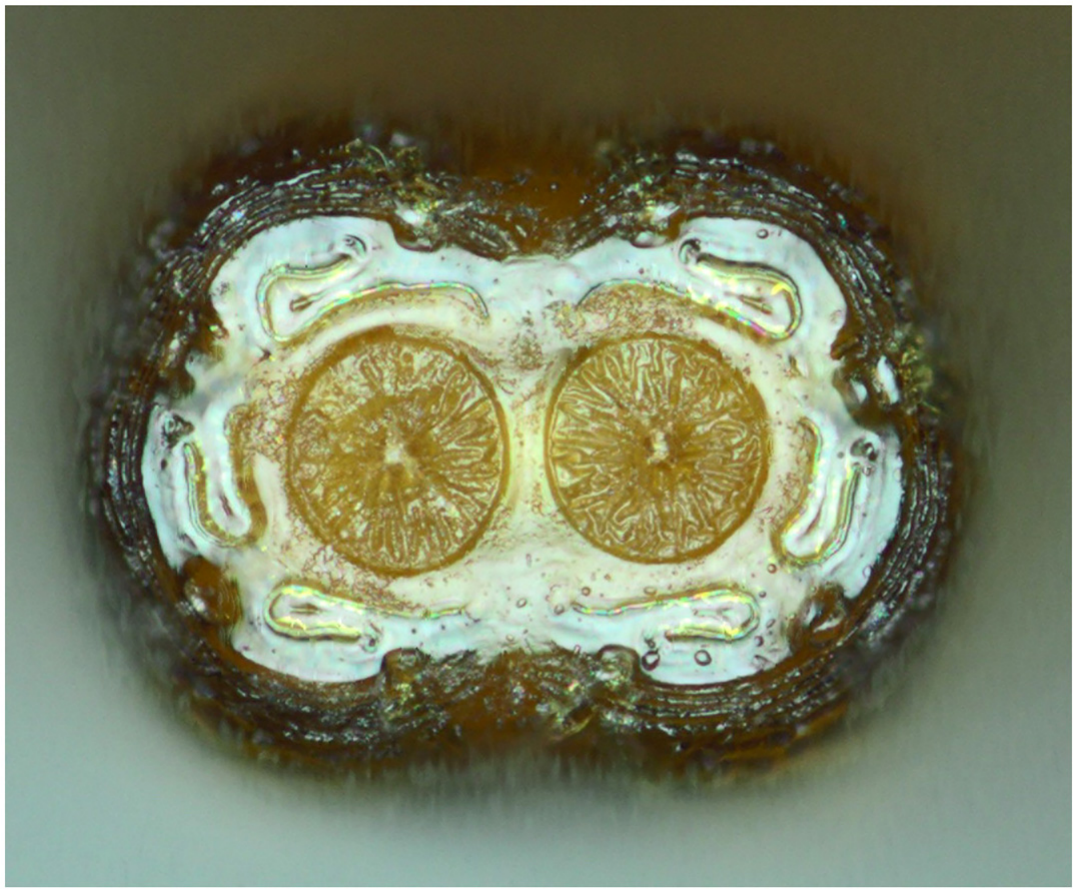
*Copestylum ellenae*, sp. nov. Holotype puparium, posterior breathing tube, apical view.

*Early stages*: third stage larva subcylindrical in cross-sectional shape, 12–15mm long, 3–5mm wide, truncate anteriorly, tapering posteriorly (Figs 35A and 36A); puparium drop-shaped (Figs 35B and 36B); projection bearing the antennomaxillary organs rugose ventrally; lateral lips coated in fine setae; anterior fold of prothorax coated in transverse rows of conspicuous, backwardly directed spicules of variable form (Fig 37A–37C); spicules extending to the dorsum of the prothorax and the antero-lateral mesothoracic margins (Fig 38B); anterior breathing tube indented more on one side and thereby tapering symmetrically and with 6+ spiracles at the apex (Figs 14D and 39); mesothoracic prolegs with a large group of variably sized crochets; antero-ventral margin of metathorax with two groups of spicules; dorsal and lateral margins coated in vestiture of variable form (Fig 40A and 40B), vestiture not or not much longer on the lateral than dorsal margins or becoming longer towards the anal segment (Figs 35A and 36A); prolegs on abdominal segments 1–3, usually with two rows of 6–8 crochets on abdominal segment 1 and reducing to 3–5 crochets on segment 3; posterior breathing tube varying in length from about half to twice the length of the anal segment (Figs 35B and 36B); tube matt at base and shiny and smooth and shiny or variably punctate beyond transverse ridge (Figs 14C and 38A); each spiracular plate with 3 pairs of curved spiracles, with the lateral spiracles U-shaped and 4 groups of interspiracular setae (Fig 41); pupal spiracles coated in inconspicuous pile, yellow to brown, curved towards tip above a matt base; pupal spiracles with 5–9 bands of encircling spiracles on the upper two thirds (Fig 14A); basal sclerite of head skeleton sclerotised black only at the dorsal bridge vertical plate and dorsal cornu; dorsal cornu short, under a third the length of the ventral cornu; labial bars and the mandibles and mandibular apodeme, narrow and sclerotised black; cibarial ridges present.

*Taxonomic notes*: adult stages of the Cinctiventre species group reared here are distinguished by their metallic colouring, uniform metallic colour of the abdominal tergites, face with a dark background colour with tomentose side-stripes (absent in *C*. *azofeifa*), scutum with short pile orientated anteriorly and long pile orientated posteriorly and lack of setae along the rear margin. However, facial colours in other Cinctiventre species are reversed, with yellow background colour and black vittae, eg *Copestylum roraima* (Curran, 1939). In general form, they are close to the Vagum and Macula species groups, but Macula group species have setae along the rear margin of the scutum and usually, lack a depression at the apex of the scutellum.

Early stages are also similar to the Vagum and Macula species groups. They are distinguished from the Macula group by the absence or poor development of prolegs with crochets on abdominal segments 5 and 6. Macula group species have prolegs with crochets on these segments and sometimes, segment 7. The uniform coating of vestiture in Vittifacium species is also a distinguishing feature; it usually forms transverse bands in the Macula group. Early stages of Cinctiventre species are distinguished from those of the Vagum species by the presence of spicules on the dorsum of the prothorax and the posterior breathing tube. Above the transverse ridge in Vagum group species, the posterior breathing tube is subcylindrical in cross-sectional shape, shiny and lightly punctured. In the Vittifacium group the posterior breathing tube is either flattened dorso-ventrally and wider above than below the transverse ridge or, if it is subcylindrical, it is conspicuously punctured, punctures light and inconspicuous in the Vagum group.

***Copestylum azofeifa*** Ricarte & Rotheray **sp. nov**. urn:lsid:zoobank.org:act: 847F324E-F472-4AE1-A286-1D1D7D4A6B24

Figs 24A, 25A, 26, 31, 32A, 32B, 37A, 40A and 42

**Fig 42:**
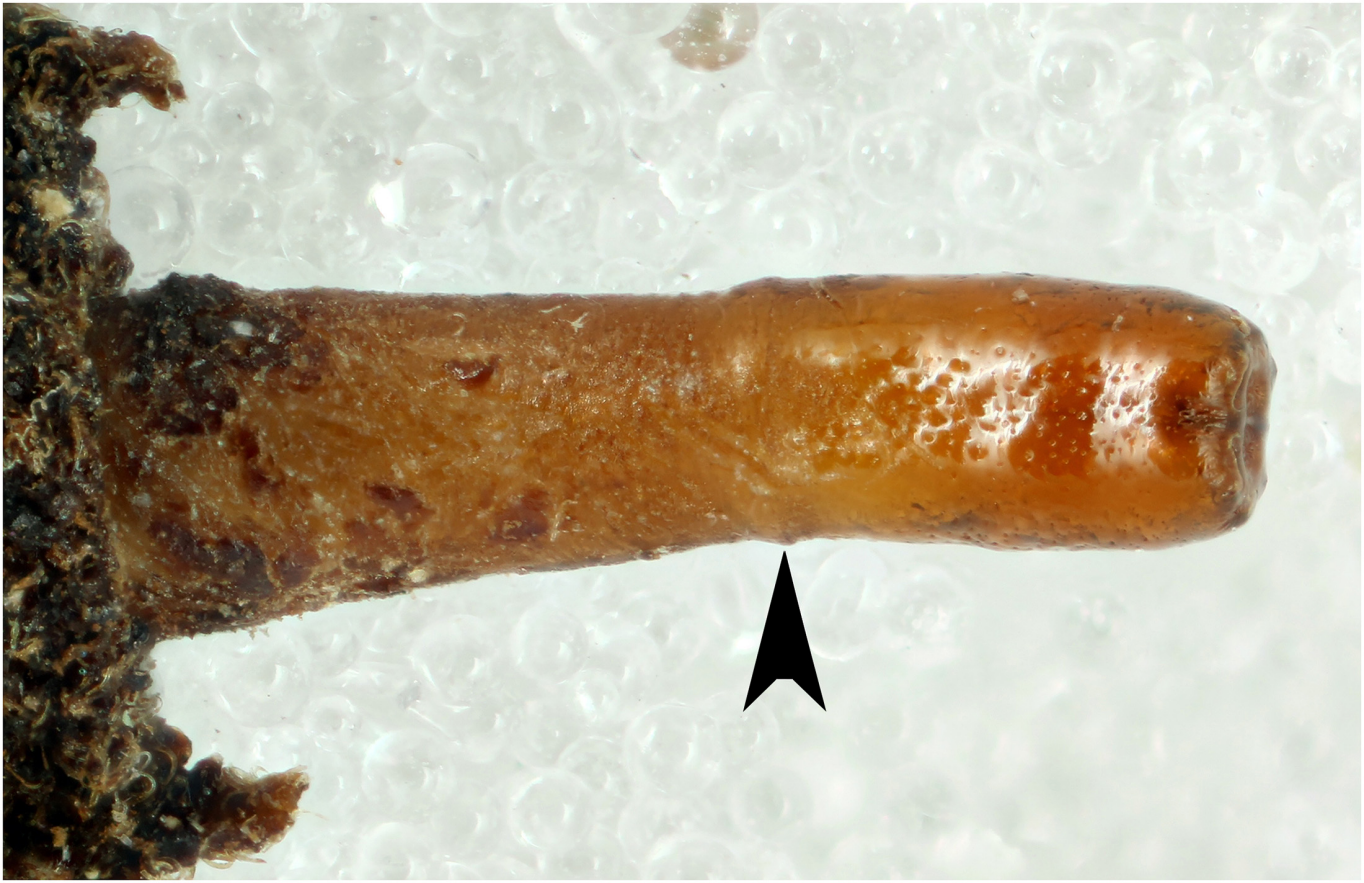
*Copestylum azofeifa*, sp. nov. Holotype puparium, posterior breathing tube, dorsal view (an arrowhead indicates position of the transverse ridge).

*Overall appearance*: sides of face with two yellow, linear vittae, dorsal one originating on the sides of frontal triangle down past the antenna and almost reaching the mouth edge, tapering only slightly; lower vitta short, less than half as long as the dorsal vitta, extending from the eye margin to the lower margin of the face; sides of face lacking tomentose macula; pleuron dark metallic except for mid-anterior margin of the anterior anepisternum and the posterior half of the posterior anepisternum clearly yellow marked.

*Adult*: length (mm): body = 11.5, wing = 10.2 (n = 1)

Male holotype

*Head*: sides of face with two yellow, linear vittae, dorsal one originating on the sides of frontal triangle down past the antenna and almost reaching the mouth edge, tapering only slightly (Figs 25A and 24A); lower vitta short, less than half as long as the dorsal vitta, extending from the eye margin to the lower margin of the face (Fig 25A); sides of face lacking tomentose maculae.

*Thorax*: pleuron dark metallic except for mid-anterior margin of the anterior anepisternum and the posterior half of the posterior anepisternum yellow marked (Fig 26); scutellum dark coloured, as the scutum, yellowish towards apex.

*Abdomen*: tergites pale red to yellowish (Fig 26); dorsum and lateral margins of T2 yellow pilose, except at upper, postero-lateral margin which has some black pile and posterior margin narrowly black pilose; T3 orange pilose in anterior half, black in posterior half; T4 orange pilose in anterior third, black in the posterior two thirds; male genitalia with surstylus triangular in lateral view, not curved back from the base and tapering towards the apex (Fig 31); hypandrium short, about 2× as long as wide and triangular in profile view (Fig 31); superior hook smoothly curved in lateral view (Fig 31) and, in apical view, apex narrowed and curving inwards (Fig 32A and 32B).

*Female*: unknown

*Early stages*: anterior fold coated in curved spicules without long bases (Fig 37A), spicules extending between the longitudinal folds on dorsum of the prothorax; antero-ventral margin of metathorax with a pair of linear bands of spicules; posterior breathing tube long, base just over half as long as apex above the transverse ridge and square at the apex (Fig 42); above the transverse ridge, posterior breathing tube slightly flattened dorso-ventrally, shining with and along the midline, a few light punctures (Fig 42); vestiture comprising long, hair-like pile (Fig 40A); upper half of pupal spiracles with 9 bands of spiracles and bent just below apex; below bands base matt and rugose becoming smooth and shiny up to the bands.

*Material examined*: *holotype*: 1♂ with puparium and genitalia dissected: COSTA RICA, Guanacaste, Caribe, falda N Volcán Tenorio, Valle río Roble, sendero a cerro Montezuma (‘track to the Montezuma hill’), N 10 41.882 W 85 00.499, 850m, L: 7.xi.2006, A: 20.xii.2006, decaying Araceae flower, leg. J.A. Azofeifa [INBio].

*Etymology*: the name in apposition ‘*azofeifa*’ refers to José Antonio Azofeifa (‘Toño’), the INBio parataxonomist who collected the holotype.

*Taxonomic notes*: the adult of this species is the most distinctive of the Cinctiventre group studied here. The vittae on the face are unique in the dorsal pair originating either side of the antennae rather than lower on the face as in other species and the bar-like shape of the lower vittae. Other unique features are the clear yellow marks on the anepisternum, the lack of tomentose maculae on the sides of the face and the smooth curve of the superior lobe in the male genitalia (Fig 31). Early stages are readily distinguished from those of other Cinctiventre species by the spicules coating the anterior fold at the front of the prothorax which have an apical hook and a short base, not longer than the apical hook is high and the lightly punctured posterior breathing tube.

*Biology*: this species was only reared from a decaying Araceae flower. It is only known from Costa Rica.

***Copestylum cinctiventre*** (Curran, 1930)

***Copestylum pinkusi*** (Curran, 1938) **syn. nov**.

Figs 14A–14D, 24B, 25B, 27, 30B–30C, 33A, 33B, 35A, 35B and 37B

*Overall appearance*: sides of face with two yellow vittae, lower one widening from the eye margin to spread over most of the ventral facial margin; pleuron mostly dark; T4 with black pile except for a pair of oval shaped areas at the anterior margin with orange pile; sterna darkened or black; in apical view, male genitalia with outer margin of superior lobe sharply angled just below apex.

*Diagnostic features*: sides of face with two yellow vittae, dorsal one extending from eye margin and tapering to almost reach the mouth edge, ventral vitta triangular-shaped, narrow at eye margin and extending broadly over the lower, side margin of the face (Figs 24B and 25B); tomentose macula overlying the dorsal vitta and extending between the two vittae (Figs 1 and 24B); pleuron with a dusky yellow macula extending from above the mid coxae to the wing base, except meron mostly dark metallic (Fig 27); T1&2 pale pilose except for the posterior margin of T2 which is black pilose and postero-lateral margins which are also black pilose; T3 black pilose except for a pair of ovals of orange pile in the anterior third; T4 anterior two thirds white pilose, posterior third black pilose; male genitalia with surstylus curved backwards just above base and tapering thereafter (as in Fig 30A); in apical view, outer margin of superior lobe sharply angled just below apex (Fig 33A and 33B); in lateral view, superior lobe with a short stem leading to the apical hook and inner margin V-shaped (Fig 30B); in lateral view, upper part of aedeagal hood with a finger-like process (Fig 30C).

*Early stages*: third stage larva as in Fig 35A, puparium as in Fig 35B; anterior fold coated in mostly, spatulate not hooked spicules (Fig 37B) which extend between the longitudinal folds on the dorsum of the prothorax (for reference, see Fig 38B); above the transverse ridge, posterior breathing tube flattened slightly, shiny and smooth, without punctures (Fig 14B); upper half of pupal spiracles with 7–9 bands of spiracles and bent just below apex so that the lines between bands are not all parallel to each other; base matt and rugose becoming smooth and shiny up to the bands (Fig 14A).

*Material examined*: *holotype*: 1♀, PANAMA, Barro Colrado Island, Canal Zone January 8 1929 [AMNH]. *Other holotypes examined for comparison*: *C*. *vittifacium*: 1♀, BRAZIL, Amazon, 66–53 / Brazil, Amazon, H.W. Bates, 66–53 (handwritten) / *Volucella vittifacia* Hull (handwritten on a red label) [NHM]; *C*. *pinkusi*: 1♂, TRINIDAD & TOBAGO, St. Anns, Trinidad, 20.viii.1933 / *Volucella pinkusi* ♂, Curran, Holotype [AMNH]. *Additional material*: TRINIDAD & TOBAGO: 1 third stage larva, Trinity Hills, Mount St. Benedict’s, 1.vii.1996, ex *Heliconia bihai* bracts, leg EG Hancock; 1 third stage larva, Chaguaramas,?.vii.1996, ex *Heliconia bihai* bracts, leg EG Hancock; 1 third stage larva 1♂, Arima Valley, L: vii–viii.1999, A: 3.ix.1999, ex *Heliconia bihai* bracts (Heliconiaceae) (Fig 36B), leg. Sharon Kennedy, Gl. Univ. Epdtn.; 1♂, Arima Valley, 19.vii−2.viii.1999, ex *Heliconia bihai* bracts, leg. Sharon Kennedy, Gl. Univ. Epdtn.; 1♂ and 1♀ with puparia, Arima Valley, 12.viii−3.ix.1999, ex *Heliconia bihai* bracts, leg. Sharon Kennedy, Gl. Univ. Epdtn.; 1♂ with puparium, Arima Valley, 23.vii−12.viii.1999, ex *Heliconia bihai* bracts, leg. Sharon Kennedy, Gl. Univ. Epdtn.; 1♂ with puparium, Arima Valley, 29.vii−12.viii.1999, ex *Heliconia bihai* bracts, leg. Sharon Kennedy, Gl. Univ. Epdtn.; 1 puparium and 1♀, Arima Valley, 16.viii−3.ix.1999, ex *Heliconia bihai* bracts, leg. Sharon Kennedy, Gl. Univ. Epdtn.; 1♀ with puparium, Arima Valley, 9−23.vii.1999, ex *Heliconia bihai* bracts, leg. Sharon Kennedy, Gl. Univ. Epdtn.; 2♀ with puparia, Arima Valley, 19.viii−3.ix.1999, ex *Heliconia bihai* bracts, leg. Sharon Kennedy, Gl. Univ. Epdtn.; 1♀ with puparium, Arima Valley, ex *Heliconia bihai*, L: 23.vii−5.viii.1999, leg. Sharon Kennedy, Gl. Univ. Epdtn.;1♂ with puparium (#3), Trinity Hills, ex *Heliconia bihai*, L: 14.vii.1998, P: 17.viii.1998, A: 1.ix.1998, Gl. Univ. Epdtn.; 1 third stage larva, 1♂ with puparium (#5), Chaguaramas, adult reared, P: 31.vii.1998, A: 11.viii.1998, Gl. Univ. Epdtn.; 1♂ with puparium (#7a), Chaguaramas, ex *Heliconia bihai*, L: 3.vii.1998, A: 17.ix.1998, Gl. Univ. Epdtn.; 1♀ with puparium (#5), Chaguaramas, L: 29.vii.1998, A: 9.viii.1998; 1♂ with puparium (#1), Cumaca, ex *Heliconia tortuosa*, L: 28.vii.1998, A: 13.ix.1998, Gl. Univ. Epdtn.; 1♂ with puparium (#1), Cumaca, ex *Heliconia tortuosa*, L: 29.vii.1998, A: 20.ix.1998, Gl. Univ. Epdtn.; 1♂ with puparium (#1), Cumaca, ex *Heliconia tortuosa*, L: 23.vii.1998, A: 15.ix.1998, Gl. Univ. Epdtn.; 1♀ with puparium (#1), Cumaca, ex *Heliconia tortuosa*, L: 28.vii.1998, A: 18.ix.1998, Gl. Univ. Epdtn.; 1♀ with puparium (#1), Cumaca, ex *Heliconia tortuosa*, L: 28.vii.1998, A: 16.ix.1998, Gl. Univ. Epdtn.; 1♀ with puparium (#1), Cumaca, ex *Heliconia*, L: 28.vii.1998, A: 25.ix.1998, Gl. Univ. Epdtn.; 1♀ with puparium, Mt Harris, ex *Heliconia tortuosa*, L: 29.vii.1998, P: 23.viii.1998, A: 3.ix.1998, Gl. Univ. Epdtn.; 1♀ with puparium (#5), Chaguaramas, L: 17.viii.1998, A: 24.viii.1998; 2♀ with puparia, Guanapo Road, 5−19.viii.1999, ex *Heliconia bihai* bracts, leg. Sharon Kennedy, Gl. Univ. Epdtn.; 2♀ with puparia, Guanapo Road, 2−19.viii.1999, ex *Heliconia bihai* bracts, leg. Sharon Kennedy, Gl. Univ. Epdtn.; 3♀ with puparia, Lopinot Rd (‘Road’), 19.viii−8.ix.1999, ex *Heliconia bihai* bracts, leg. Sharon Kennedy, Gl. Univ. Epdtn.; 1♀ with puparium, Lopinot Rd, 26.vii−12.viii.1999, ex *Heliconia bihai* bracts, leg. Sharon Kennedy, Gl. Univ. Epdtn.; 2♀ with puparia, Lopinot Rd, 23.vii−3.viii.1999, ex *Heliconia bihai* bracts, leg. Sharon Kennedy, Gl. Univ. Epdtn [HM, NMS & CEUA]. COSTA RICA: 1♂ (CR102) and 1♀ (CR101) with puparia and male genitalia dissected, Braulio Carrillo, 22.ix.2006, ex *Heliconia latispatha*, 550m (♂), 500m (♀), leg. M.A. Marcos-García; 2♂ (CR94, 95) with puparia and genitalia dissected, Braulio Carrillo, 31.x.2006, 550m, leg. M.A. Marcos-García; 1♀ (CR104) with puparium, Braulio Carrillo, 13.xi.2006, 550m, leg. M.A. Marcos-García; 1♀ (CR96) with puparium, Braulio Carrillo, 2.xi.2006, 550m, leg. M.A. Marcos-García; 1♂, Limón, Talamanca, Puerto Viejo, 5km, LS_400800N 599900E, ex heliconia flower, L: 22.ii.1999, A: 31.iii.1999, leg. G.E. Rotheray (INB0003056088); 4♂ and 8♀ with puparia (males: INB0003056069, INB0003056067, INB0003056079, INB0003056080; females: INB0003056066, INB0003056068, INB0003056081, INB0003056082, INB0003056070, INB0003056078, 2♀ without INBio codes), Puntarenas, Osa, Puerto Jiménez, estación Los Patos, ex heliconia flowers, L: 9.ii.1999, A: 14.iii.1999, leg. M. Lobo; 1♂ (INB0003056087) and 1♀ (INB0003056086), Limón, Talamanca, Puerto Viejo, 5km, LS_400800N 599900E, ex heliconia flower, L: 22.ii.1999, A: 31.iii.1999, leg. M.A. Zumbado, leg. G.E. Rotheray [INBio].

*Taxonomic notes*: *Copestylum cinctiventre* was introduced by Curran [10] on the basis of a female collected from Panama and *C*. *pinkusi* was introduced by Curran [11] on the basis of 3 males collected from Trinidad. Curran comments that *C*. *pinkusi* is related to *C*. *cinctiventre*, the difference between the two being in the colour of the pile on the scutum, being pale in *C*. *cinctiventre* and dark in *C*. *pinkusi*. However, based on the reared material examined here, this is a sexual difference. Other differences between the descriptions of the two taxa provided by Curran [10, 11] are also probable sexual differences, such as the colour of the abdominal pile. However, within our material, both males and females are variable in some of their colours. For instance, the basoflagellomere varies from being yellow to being blackish; the pile on T3 may or may be all black or black with orange pile anteriorly. The sternites, particularly S2, can be yellow or be darkened almost black. The background colour of hind tibiae can be dark yellow to black. Across male colour variants and between males reared from Costa Rica and Trinidad, no consistent differences in gross morphology, male genitalia and early stages were found. On these grounds *C*. *pinkusi* is here synonymised under *C*. *cinctiventre*.

*C*. *cinciventre* is similar to *C*. *vittifacium* (Hull, 1943) but, on the basis of comparing the holotypes, these two species can be separated by the following characters: in *C*. *cinctiventre*, the face is more produced forwards and the lateral yellow vittae are more conspicuous; the scutellum has pale pile; metabasitarsomere darker; T2&3 with pale pile only, while in *C*. *vittifacium* the T2 with black pile antero-laterally, and the T3 has a band of black pile posteriorly. *C*. *cinctiventre* differs from *C*. *roraima*, a Cinctiventre group species also reared from *Heliconia* flowers [12], in that the face of *C*. *roraima* has a yellow, not a black, background colour.

*C*. *cinctiventre* is distinguished from other Cinctiventre species reared here by sides of the face with two vittae, the dark anepisternum lacking yellow marks, superior lobe of the male genitalia with a short stem, not much longer than the length of the apical hook (Fig 30B). Early stages are readily distinguished by the spatulate form of the spicules coating the anterior fold at the front of the prothorax.

*Biology*: adults were reared in Costa Rica and Trinidad from heliconia flowers (Heliconiaceae), most frequently *H*. *bihai*, but also *H*. *latispatha* and *H*. *tortuosa*.

***Copestylum ellenae*** Rotheray & Ricarte **sp. nov**. urn:lsid:zoobank.org:act: F7CA1305-00CD-410B-968F-E25799C6B204

Figs 25C, 28, 29, 30A, 34A, 34B, 36A, 36B, 37C, 38A, 38B, 39, 40B and 41

*Overall appearance*: sides of face with one yellow vitta; T4 black pilose except for a pair of oval shaped areas of orange pile at the anterior margin; sterna darkened or black; male genitalia with superior lobe having a long stem, longer than the length of the apical hook.

*Adult*: length (mm): body = 9.7−10.3 (n = 2), wing = 7.8 (n = 1)

Male holotype

*Head*: sides of face with one yellow vitta, extending from eye margin and tapering to almost reach the mouth edge (Fig 25C); this vitta almost completely covered in tomentum and a smaller tomentose macula beneath.

*Thorax*: pleuron mostly dark, except anterior margin of katepimeron narrowly yellow, otherwise dark metallic (Fig 28).

*Abdomen*: T2 pale pilose except black on the posterior and lateral margins; T3 black pilose except for a pair of narrow, dark orange pilose fasciae on the anterior margin; T4 white pilose on the anterior two thirds and black elsewhere including the lower lateral margins (Fig 29); male genitalia with surstylus curved backwards just above base and tapering thereafter (Fig 30A); in apical view, outer margin of superior lobe corrugated (Fig 34A and 34B); in lateral view, inner margin of superior lobe U-shaped (Fig 30A); in lateral view, upper part of aedeagal hood triangular.

*Female*: unknown.

*Early stages*: third stage larva as in Fig 36A, puparium as in Fig 36B; anterior fold coated in spicules with long bases, base 2× or more than length of terminal hook, spicules extending between the longitudinal folds on dorsum of the prothorax (Figs 38B and 37C); antero-ventral margin of metathorax with an almost complete band of similarly long-based spicules in at least 2 rows; posterior breathing tube short, base less than half as long as apex above the transverse ridge and tapering towards the apex (Figs 36B and 38A); above the transverse ridge, matt with heavy, deep punctures making the basal two thirds of the tube appear nodulate (Fig 38A); spiracular plate with 3 pairs of curved spiracles and 4 groups of interspiracular setae (broken off) (Fig 41); anterior breathing tube with 7+ spiracles across the apex (Fig 39); vestiture short and spicule-like (Fig 40B); upper half of pupal spiracles with 5 bands of spiracles and bent just below apex; below bands base matt and rugose becoming smooth and shiny up to the bands.

*Etymology*: The epithet ‘*ellenae*’ meaning ‘Ellen’s’ refers to Ellen Louise Rotheray, G.E. Rotheray’s daughter.

*Material examined*: *holotype*: 1♂ with puparium and genitalia dissected: COSTA RICA, Guanacaste National Park, San Gerardo, ex flower of *Renealmia* L. (Zingiberaceae), (INB0003055978) (Fig 41), L: 25.vi.2000, A: 6.vii.2000, leg. G.E. Rotheray [INBio].

*Paratype*: 1♂ with puparium and genitalia dissected (INB0003055979) and 3 third stage larvae, same data as holotype [INBio, NMS].

*Taxonomic notes*: the adult of this species is very similar to *C*. *cinctiventre*. It is distinguished from that species by the sides of the face with one vitta and the superior lobe of the male genitalia which has a corrugated outer margin (Fig 34A and 34B) and a U-shaped inner margin between the apical hook and the base (Fig 30A). Early stages are readily distinguished by the short, punctured posterior breathing tube, spiculate vestiture and spicules of the anterior fold with long bases.

*Biology*: adults were reared from *Renealmia* flowers (Zingiberaceae). This species is only known from Costa Rica.

### Key to species groups and species reared from live and dead fruits and flowers in the present study

#### Adults

1. Face with a pale background colour (Fig 3A–3D); T1 and anterior part of T2 with a conspicuous pale mark compared to the rest of the abdominal tergites (Fig 11A).......... 2 (Vagum species group)
  - Face with a dark background colour (Fig 24A and 24B); T1 and anterior part of T2 with a similar colour pattern to the rest of the abdominal tergites (Fig 29).......... 8 (Cinctiventre species group)
2. T3&4 with yellow marks or completely black.......... 4
  - T3&4 green to orange with a narrow, black, apical margin.......... 3
3. Pleuron black-marked; dorsal margin of eyes sloping (Fig 9).......... *C*. *vagum*
  - Pleuron without black marks, uniformly yellow and orange; dorsal margin of eyes flattened (Fig 7).......... *C*. *tenorium* sp. nov.
4. Thoracic setae black (Fig 10); mid femora with entirely dark pile.......... 5
  - Thoracic setae orange (Fig 9); mid femora with at least some pale pile.......... 6
5. Face with central vitta strongly pigmented, shining black (Fig 3A); pleuron with a U-shaped yellow macula (Fig 5); femora jet black.......... *C*. *araceorum* sp. nov.
  - Face with central vitta faintly pigmented black (cf Fig 3B); pleuron with a W-shaped black macula (Fig 10); femora dark yellow.......... *C*. *willistoni* sp. nov.
6. Eyes more than half as long as high (Fig 4B); face with central vitta present, even if faint (Fig 3B)… *C*. *cyclops* sp. nov.
  - Eyes less than half as long as high (Fig 4D); face without central vitta (cf Fig 3D).......... 7
7. Tibiae jet black; male cerci triangular-shaped (Fig 19A).......... *C*. *tigrinium* sp. nov.
  - Tibiae dark yellow; male cerci L-shaped (Fig 17A).......... *C*. *musicanum*
8. Posterior anepisternum with a clear yellow mark (Fig 26); vittae on the sides of the face extending up to yellow side margins of the frontal triangle (Fig 24A).......... *C*. *azofeifa* sp. nov. (female unknown)
  - Posterior anepisternum black, without a yellow mark (Figs 27 and 28); vitta on the sides of the face only reaching lower eye margin, not extending to the frontal triangle which is completely dark or black (Fig 24B).......... 9
9. Sides of face only with one vitta (Fig 25C); superior lobe with inside margin of apical hook U-shaped (Fig 30A) and outer margin corrugated (Fig 34A and 34B).......... *C*. *ellenae* sp. nov.
  - Sides of face with two vittae (Fig 25A); superior lobe with inside margin of apical hook V-shaped (Fig 30B) and side margin not corrugated, outer margin angled (Fig 33A and 33B)…*C*. *cinctiventre*

#### Early stages (third stage larva and puparium)

1. Prolegs and crochets on abdominal segments 1–4, sometimes 5&6; dorsum of prothorax lacking spicules; reared mainly from fruits.......... 2
  - Prolegs and crochets on abdominal segments 1–3; dorsum of prothorax with spicules (Fig 38B); reared from flowers and Araceae spathes.......... 8
2. Prolegs and crochets on abdominal segments 1–6.......... 3
  - Prolegs and crochets on abdominal segments 1–4.......... 4
3. Pupal spiracles short, about as far apart as they are long (Fig 13A); apex of posterior breathing tube smooth and shining, with light puncturing (Fig 13B)… *C*. *araceorum* sp. nov.
  - Pupal spiracles long, much longer than their distance apart (Fig 14A); apex of posterior breathing tube with obvious punctures.......... *C*. *willistoni* sp. nov.
4. Posterior breathing tube short, less than body width, transverse ridge at about mid-point (Fig 15B).......... 5
  - Posterior breathing tube long, about or more than body width, transverse ridge well above the mid-point (Fig 18).......... 6
5. Pupal spiracles with lower 4 encircling bands interrupted on both dorsal and ventral margins; apex of posterior breathing tube conspicuously punctured (Fig 15B).......... *C cyclops* sp. nov.
  - Pupal spiracles with the lower 4 encircling bands interrupted only one side; apex of posterior breathing tube with light, inconspicuous punctures.......... *C*. *tenorium* sp. nov.
6. Posterior breathing tube with a few conspicuous punctures.......... 7
  - Posterior breathing tube smooth, without punctures.......... *C*. *musicanum*
7. Posterior breathing tube tapering from base to apex, subcylindrical in cross-sectional shape; prolegs with crochets absent or reduced and inconspicuous on abdominal segment 5.......... *C*. *vagum*
  - Posterior breathing tube widened and flattened at apex, so that apex is wider than width across the transverse ridge; prolegs with crochets present on abdominal segment 5, if reduced still obvious.......... *C*. *tigrinum* sp. nov.
8. Vestiture long, hair-like and obvious (Fig 40A).......... 9
  - Vestiture short and inconspicuous, like short, stunted spicules (Fig 40B); anterior fold with spicules on elongate bases and a short apical hook (Fig 37C); posterior breathing tube less than half body width and heavily punctured (Fig 38A).......... *C*. *ellenae* sp. nov.
9. Anterior fold with spicules on short bases with an apical hook (Fig 37A); posterior breathing tube lightly punctured (Fig 42).......... *C*. *azofeifa* sp. nov.
  - Anterior fold with most spicules spatulate without apical hooks (Fig 37A); posterior tube smooth and shining, not punctured (Fig 14B).......... *C*. *cinctiventre*

## Discussion

### Taxonomy

The ten species dealt with in this paper possess the key characters of the genus *Copestylum*: plumose arista, recurrent apical cross vein and bare anterior anepisternum and posterior anepimeron. All share a similar body size, shiny face, depression at the apex of the scutellum and absence of bristle-like setae at the rear margin of the scutum. Seven have the pile of the scutum orientated mostly posteriorly and T1&2 pale in colour, contrasting with the background colour of the rest of the thorax and abdomen. These are referred to here as the Vagum species group. The remaining three species have metallic colouring, scutum with short pile orientated anteriorly and longer pile orientated posteriorly and T1&2 without a contrasting background colour to the rest of the thorax and abdomen. They are referred to as the Cinctiventre species group.

A group identifying character in the male genitalia is the apex of the hypandrium which has two elements, the superior lobe dorsally and the aedeagal hood ventrally. In Vagum species the aedeagal hood projects ventrally from the hypandrium and the surface of the superior lobe is smooth. In the Cinctiventre group, the aedeagal hood projects dorsally, almost between the hooked arms of the superior lobe which has slight projections, except *C*. *azofeifa* which lacks a dorsal projection to the aedeagal hood. Otherwise, in both groups, male genitalia have useful distinguishing characters at species level with variation found in the shape of the cercus, surstylus and superior lobe. Nonetheless, genitalia were more uniform in the Cinctiventre than the Vagum group, although fewer species were examined. Apart from genitalia, major axes of variation in the Vagum group are the facial vittae, leg colour and the colour of pile coating them and the colour pattern of T3&4. Axes of variation in the Cinctiventre group are the states of facial vittae and tomentose marks and colour of the pleuron.

Early stages also possess the key characters of the genus *Copestylum*: inverted mandibles and anal segment divided into two sections, the basal section of which has two pairs of lappets of unequal length. Larvae of both species groups share the characters of linear rows of spicules on the antero-ventral, metathoracic margin, prolegs and crochets most developed on segments 1–3 or 4, usually absent on abdominal segments 5 and 6 and always absent on segment 7. Vagum group larvae possess the following distinguishing features: dorsum of the prothorax without spicules; anterior breathing tube symmetrical, not pinched more on one margin and with 4–6 spiracles linearly arranged across the apex, prolegs with crochets on abdominal segments 1–4, sometimes extending to abdominal segments 5 and 6 and, bands of spiracles almost reaching the base of the pupal spiracles. Across the Vagum group species studied here, larvae are relatively uniform morphologically with the main axes of variation being minor differences in the size, shape and degree of puncturing of the posterior breathing tube. Cinctiventre group larvae possess the following distinguishing features: spicules extending from the anterior fold on to the dorsum of the prothorax; anterior breathing tube asymmetrical, outer side indented more than the other with 6+ spiracles in a curved line across the apex, prolegs and crochets only on abdominal segments 1–3 and bands of spiracles on about two thirds the length of the pupal spiracles. Across the Cinctiventre species studied here, larvae are more disparate than Vagum group larvae with major variation in the form of the spicules coating the anterior fold, body vestiture and the length and degree of puncturing of the posterior breathing tube.

Adult colour variations are frequent within Vagum group species. The taxonomic significance of such variation is difficult to assess. It may be age-related, marks becoming stronger and darker with age, as is known for example, in the Palaearctic syrphid, *Hammerschmidtia ferruginea* (Fallén, 1817) [13]. It may be related to rearing temperature, as is known in certain Palaearctic syrphines [14]. It may be post-mortem, such as green changing gradually to yellow or orange. Post-mortem change makes taxonomic comparison between recently and older collected specimens difficult, especially if specimens have in addition, faded. Suggesting that colour variation may be inherited, some colour combinations appear confined geographically, for example in *C*. *vagum* and *C*. *cinctriventre* as described above. Locality-associated colour patterns could be trivial, but could also indicate species complexes. Based on gross assessments of morphology presented here, it was not possible to determine the significance of colour varieties and we have treated them conservatively. Following this approach, we synonymise *C*. *pinkusi* under *C*. *cinctiventre* on the basis that the colour differences between them are sexual and no morphological evidence was found in either the adult or early stages to support a *pinkusi* species concept.

### Development sites

**Fig 43:**
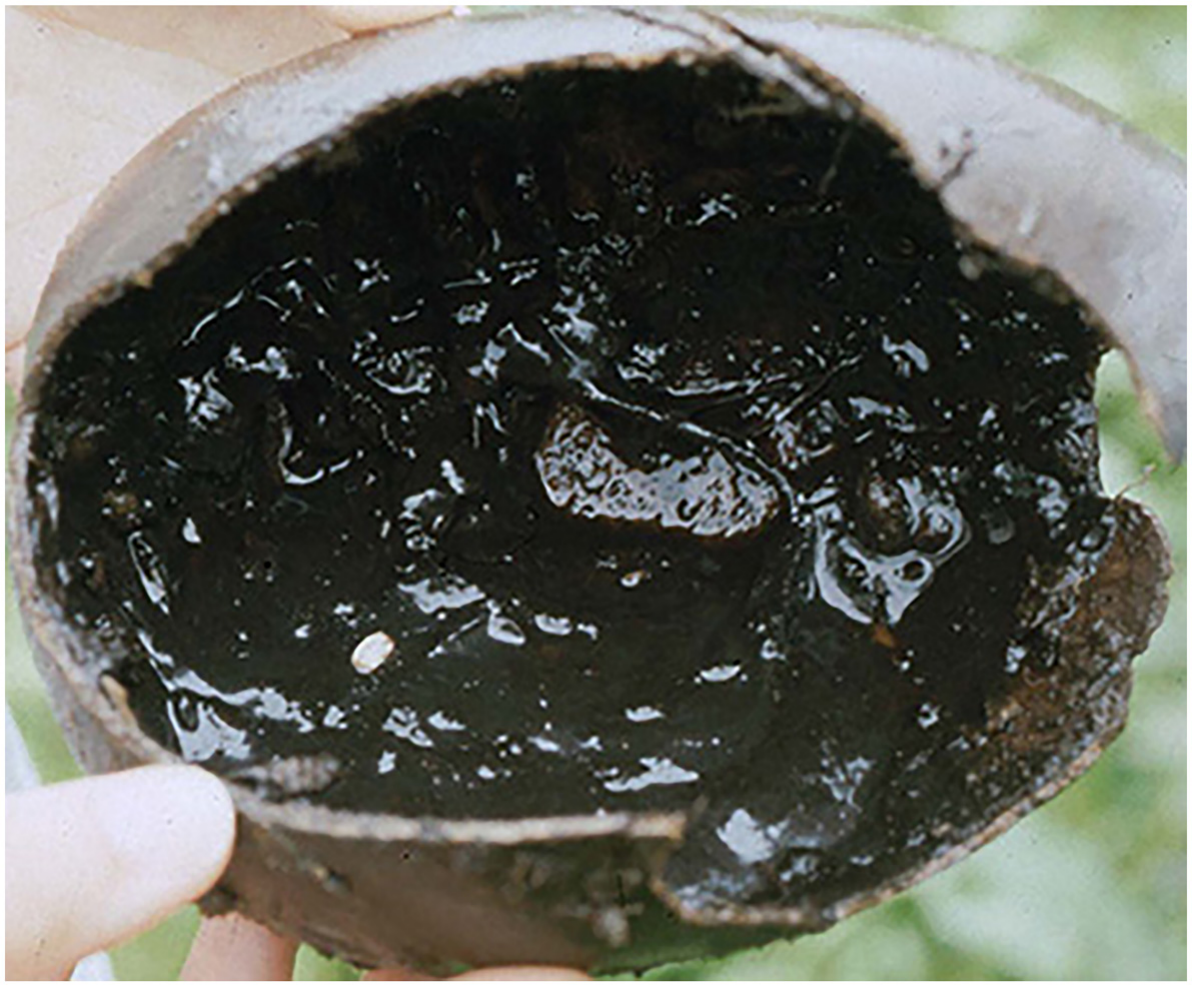
Vagum species group development site. Decaying Sapotaceae fruit, Guanacaste National Park, Costa Rica.

Development sites almost completely separate Vagum and Cinctiventre groups. Vagum species were mainly reared from decaying fruits (Fig 43) and Cinctiventre species from flowers (Figs 35C and 36C). Except that is, for the Vagum group species, *C*. *araceorum* and the Cinctiventre group species, *C*. *azofeifa*, both of which were only reared from Araceae spathes i.e. a spike of flowers enclosed within a bract (Fig 12C).

Larvae of Vagum group species were most frequent in decaying fruits that had fallen to the ground, although occasionally, they were found within decay on fruits still on the tree or shrub. Usually, several larvae were present, suggesting multiple oviposition by more than one female or batched egg laying by an individual. The size of fruits containing larvae suggested that size per se was not a factor limiting larval abundance. A more significant factor appears to be the amount of decay. Decaying fruits were not, however, the only development site used by Vagum group species. *C*. *cyclops* was also reared from decaying material under epidermal tissue of an understorey plant, *C*. *musicanum* and *C*. *tenorium* from wet decay within dead palm stems (Palmaceae), *C*. *tenorium* from a decaying bromeliad (Bromeliaceae) and *C*. *willistoni* from exuding tree sap.

Larvae of the three Cinctiventre group species studied here develop within flowers enclosed either by bracts, such as the *Heliconia* flowers from which *C*. *cinctriventre* was reared and the Araceae spathe from which *C*. *azofeifa* was reared or, a tubular calyx, such as the *Renealmia* flowers from which *C*. *ellenae* was reared. Water was associated with enclosed flowers, either through rainfall being trapped or a by-product of decay processes or both. Wet conditions facilitate the build-up of microbial populations which probably provide opportunities for filter-feeding, saprophagous *Copestylum* larvae. Unlike Vagum group species, Cinctiventre species were not reared from other development sites and they are probably more specialised. Further evidence of specialisation are morphological and behavioural features that appear to match particular conditions of access and food quality characterising development sites, see next section.

In being specialised, Cinctiventre species are probably more vulnerable to downturns and extinctions than Vagum species. This is because under conditions of host plant declines, switching to another development site is not likely. A corollary of this is that they probably make better environmental indicators than Vagum group species, their abundance being more closely tied to that of their host plants. This potential requires, however, improved understanding of their distribution and abundance relative to that of their host plants.

As with previous studies of bromeliads and cacti [2, 5] and with other species surveys of Neotropical Syrphidae [15], searching for development sites and rearing larvae is a particularly effective means of inventory. Given the proportionally limited geographical areas over which we worked, many additional *Copestylum* species developing in fruits and flowers almost certainly await discovery.

### Functional morphology

Adult *Copestylum* colour patterns conform to the expectations of mimicry in open habitats such as deserts and crypsis in shaded habitats, such as the understorey of forests [2, 5]. Species of the two groups considered here were mainly reared in shaded or partially shaded habitats and their colour patterns are as expected, cryptic rather than mimetic. For instance, Vagum group species all possess a conspicuous, pale mark at the base of the abdomen. This appears to be disruptive in function, in that it draws the attention of an observer more than the shape of the body. Disruptive marks are a feature of many cryptic animals [16]. For visually hunting predators such as birds, they complicate formation of an effective search image. Search image formation is possibly further complicated by intraspecific variation in body colours that, as noted above, characterises most Vagum group species examined here. Such variation is not apparent in Cinctiventre group species and they are overall, less colourful.

Adult Cinctiventre species do not have such obvious disruptive marks. Instead, the overall body pattern is dark overlain with pile which varies in colour, size and orientation, especially that covering the scutum and abdominal tergites. Furthermore, the pleuron is patchily covered in dust and the face has contrasting yellow vittae and tomentose maculae. The effect is an ever-changing kaleidoscope of flashing light and dark as the fly or observer move relative to one another. In the shade, reflective colours such as these make animals bearing them difficult to visualise and hence, disguise them from visually hunting predators [16].

In bromeliad and cactus developing *Copestylum*, considerable variation exists in the shape and colours of the face and the morphology of the male genitalia [2, 5]. Similarly, many Vagum and Cinctiventre species examined here, have species-specific facial colours and features of the male genitalia. A possible explanation of such a pattern is that mate selection involves visual cues based on the face and tactile cues from the genitalia. In support of visual cues, in males but not females, facets are larger on the top of the eyes and protected by longer pile than elsewhere. Furthermore, in some Vagum group species, this part of the head is flattened and facets here are particularly large. In one new species described here, this is recognised by the use of the epithet, *cyclops*, which refers to this condition.

Vagum group larvae have relatively uniform and unmodified body shapes and lack specialised spicules. This is unlike many *Copestylum* species developing within decaying bromeliads and cacti whose larvae have diverse body shapes, each apparently matching particular conditions of access and large, hook-like spicules or, the anterior end coated in extensive and variable arrangements of small spicules [2, 5]. Extensive and modified spicules not only protect the larval integument during movement, they also loosen and expose relatively fresh tissue to microbial decay, thereby creating more food and mutualisms between larvae and microbes probably exist. Plant tissues containing such larvae decay at more rapid rates than tissue without them [17]. Vagum group larvae are probably less effective at spreading decay relative to species with specialised spicules. In other words, Vagum group larvae appear to be relatively generalised in form and function, feeding on decay that is easily accessible and of an oily to soft solid consistency.

This is not the case with *C*. *cinctiventre* and *C*. *ellenae*. Unlike development sites used by *C*. *azofeifa* and most Vagum group species, access is limited within *Heliconia* and especially, *Renealmia* flowers. Only one larva per flower was recorded and *Heliconia* flowers are relatively long, while those of *Renealmia* are short. In *Heliconia* flowers, *C*. *cinctiventre* larvae possess the remarkable facility to extend their bodies, 2× or more longer than an unextended larva. These larvae were usually found in extended positions in the narrow space between the flower and the lower margin of the bract with the head pressed up against the base. When removed, they contracted rapidly and lost this shape. *C*. *ellenae* larvae take up almost the entire space within the calyx of *Renealmia* flowers and assumed within them, the opposite state of a short, contracted and broad body that was similarly lost when removed. Hence these larvae are able to manipulate body shape to fit access conditions at development sites.

For larvae of *C*. *cinctiventre* and *C*. *ellenae*, access conditions at development sites not only requires them to manipulate and squeeze their bodies into confined spaces, in front of them, they remove compacted layers of food or biofilm i.e. matrix enclosed, microbial accretions [18]. This is in contrast to *C*. *azofeifa* and Vagum group larvae that characteristically have more space and are immersed in oily to soft-solid food. Unlike *C*. *azofeifa* and Vagum group species, the anterior folds of *C*. *cinctiventre* and *C*. *ellenae* are coated in modified spicules, spatulate in *C*. *cinctiventre* and long-based in *C*. *ellenae*. These different types of spicules are probably alternative solutions for loosening and removing compacted biofilm coating surfaces inside the flower or, they help remove plant tissue which subsequently decays to provide food. If the latter, these larvae may have mutualisms with microbes in a similar way to some bromeliad and cacti developing larvae mentioned above. Amounts of wet decay and available space within Araceae spathes and decaying fruits is, by comparison, much greater and in *C*. *azofeifa* and Vagum group larvae, the spicules on the anterior fold are unspecialised, having the usual, hook-like form of most saprophagous syrphid larvae.

The larva of both *C*. *cinctiventre* and *C*. *ellenae* differ in additional ways relative to *C*. *azofeifa*. The posterior breathing tube of *C*. *ellenae* is the shortest of all those studied here, less than half the length of the breathing tube above the transverse ridge. The larvae of *C*. *ellenae* do not require a long breathing tube because the calyx of *Renealmia* flowers is about as long as the larva and a short breathing tube is sufficient to reach the open air. In contrast, the breathing tube of *C*. *cinctiventre* is the longest of the 3 Cinctiventre species and in *Heliconia* flowers which are themselves long, length is probably required to enable the larva to project the tip above the water for air exchange. Finally, the larva of *C*. *ellenae* has specialised vestiture comprising stunted, spicule-like pile (Fig 40B). Vestiture is probably modified in *C*. *ellenae* to facilitate protection and/or holding on via frictional forces since, within the confined space of a *Renealmia* calyx, it is not just the ventral but also the lateral and dorsal aspects of the body that are in contact with the calyx. To pupate, the larva of *C*. *ellenae* turns round in the calyx and pupates within it which facilitates the adult escaping from the development site. The integument has a reddish tinge anteriorly, reminiscent of the colour of *Renealmia* seeds and in situ, the two can be difficult to tell apart. This might be protective resemblance in the puparium of *C*. *ellenae*. The puparium of *C*. *cinctiventre* was never found in situ and it seems probable that the larva pupates outside the flower.
